# Quality Assessment of a Large Multi-Center Flow Cytometric Dataset of Acute Myeloid Leukemia Patients—A EuroFlow Study

**DOI:** 10.3390/cancers14082011

**Published:** 2022-04-15

**Authors:** Anne E. Bras, Sergio Matarraz, Stefan Nierkens, Paula Fernández, Jan Philippé, Carmen-Mariana Aanei, Fabiana Vieira de Mello, Leire Burgos, Alita J. van der Sluijs-Gelling, Georgiana Emilia Grigore, Jacques J. M. van Dongen, Alberto Orfao, Vincent H. J. van der Velden, on behalf of the EuroFlow Consortium

**Affiliations:** 1 Department of Immunology, Erasmus MC, University Medical Center Rotterdam, Wytemaweg 80, 3015 CN Rotterdam, The Netherlands; anne.bras@catharinaziekenhuis.nl; 2 Cancer Research Center (IBMCC-CSIC), Department of Medicine and Cytometry Service (NUCLEUS), University of Salamanca, CIBERONC and Institute of Biomedical Research of Salamanca (IBSAL), Campus Miguel de Unamuno, Paseo de la Universidad de Coimbra s/n, 37007 Salamanca, Spain; smats@usal.es (S.M.); orfao@usal.es (A.O.); 3 Princess Máxima Center for Pediatric Oncology, Heidelberglaan 25, 3584 CS Utrecht, The Netherlands; s.nierkens-2@prinsesmaximacentrum.nl; 4 Institute for Laboratory Medicine, Kantonsspital Aarau AG, Tellstrasse 25, 5001 Aarau, Switzerland; paula.fernandez@ksa.ch; 5 Department of Diagnostic Sciences, Ghent University, C. Heymanslaan 10, 9000 Ghent, Belgium; jan.philippe@ugent.be; 6 Laboratory of Hematology, University Hospital of Saint-Etienne, Av. Albert Raimond, 42055 Saint-Etienne, France; cmaanei@gmail.com; 7 Cytometry Service, Institute of Pediatrics (IPPMG), Faculty of Medicine, Federal University of Rio de Janeiro (UFRJ), Rua Bruno Lobo 50, Cidade Universitária, Rio de Janeiro 21941-912, RJ, Brazil; fabivmello@gmail.com; 8 Applied Medical Research Center (CIMA), Instituto de Investigacion Sanitaria de Navarra (IDISNA), Clinica Universidad de Navarra, 31008 Pamplona, Spain; lbrodriguez@unav.es; 9 Department of Immunology, Leiden University Medical Center (LUMC), Albinusdreef 2, 2333 ZA Leiden, The Netherlands; a.j.van_der_sluijs-gelling@lumc.nl (A.J.v.d.S.-G.); j.j.m.van_dongen@lumc.nl (J.J.M.v.D.); 10 Cytognos SL, Carretera de Madrid Km. 0 Nave 9, Pol. La Serna, Santa Marta de Tormes, 37900 Salamanca, Spain; ggrigore@cytognos.com; 11 Cancer Research Center (CIC), Department of Medicine, University of Salamanca, 37007 Salamanca, Spain

**Keywords:** quality assessment, AML, flow cytometry, EuroFlow

## Abstract

**Simple Summary:**

Flow cytometry allows detailed characterization of large numbers of cells and plays an important role in the diagnosis of acute myeloid leukemia. To facilitate analysis of flowcytometric data, reference databases of normal bone marrow samples and samples from acute myeloid leukemia patients, together with new software tools, are required. We here report on the building of a large database of acute myeloid leukemia patients (*n* = 1142) and 22 normal samples. We report on the quality assessment procedure used and its validation, discuss potential pitfalls, and provide possible solutions for avoiding such flaws in the construction of other databases. Our data show that obtaining and collecting reproducible flow cytometric data over time and across centers is feasible, but also that strict quality assessment remains crucial, even when standardized protocols for staining and instrument settings are being used in a multicenter setting.

**Abstract:**

Flowcytometric analysis allows for detailed identification and characterization of large numbers of cells in blood, bone marrow, and other body fluids and tissue samples and therefore contributes to the diagnostics of hematological malignancies. Novel data analysis tools allow for multidimensional analysis and comparison of patient samples with reference databases of normal, reactive, and/or leukemia/lymphoma patient samples. Building such reference databases requires strict quality assessment (QA) procedures. Here, we compiled a dataset and developed a QA methodology of the EuroFlow Acute Myeloid Leukemia (AML) database, based on the eight-color EuroFlow AML panel consisting of six different antibody combinations, including four backbone markers. In total, 1142 AML cases and 42 normal bone marrow samples were included in this analysis. QA was performed on 803 AML cases using multidimensional analysis of backbone markers, as well as tube-specific markers, and data were compared using classical analysis employing median and peak expression values. Validation of the QA procedure was performed by re-analysis of >300 cases and by running an independent cohort of 339 AML cases. Initial evaluation of the final cohort confirmed specific immunophenotypic patterns in AML subgroups; the dataset therefore can reliably be used for more detailed exploration of the immunophenotypic variability of AML. Our data show the potential pitfalls and provide possible solutions for constructing large flowcytometric databases. In addition, the provided approach may facilitate the building of other databases and thereby support the development of novel tools for (semi)automated QA and subsequent data analysis.

## 1. Introduction

Flowcytometric analysis allows for rapid acquisition of multiple parameters from large numbers of cells and typically results in complex multidimentional datasets. Current diagnostic flow cytometers can measure at least eight fluorescent parameters (e.g., eight antibodies) and two light scatter characteristics (i.e., forward scatter (FSC) and side scatter (SSC)) simultaneously from millions of single cells in short periods of time [1]. For the evaluation of patients with acute myeloid leukemia (AML), the EuroFlow consortium has developed an eight-color antibody panel, consisting of six tubes with distinct purposes [2]. Each tube contains four so-called backbone (BB) antibodies (i.e., CD34, CD117, CD45, and HLADR), which allow for appropriate identification of AML cells in virtually every patient [2] and consistently across all six tubes. In addition, each tube contains four tube-specific (TS) antibodies, which allow for a more detailed characterization of the AML cells, as well as a more detailed identification of normal cells.

Given the ever-increasing complexity of flow cytometric data (more cells and parameters, mainly due to technological advances), novel tools for data analysis have been and are being developed [3,4,5]. Traditional manual analysis heavily depends on two-dimensional analysis (i.e., gating in two-dimensional dot plots) and cytometrist expertise (i.e., their ability to distinguish normal and leukemia cells based on their characteristic immunophenotypic profiles). In contrast, modern flow cytometric analysis software allows for multidimensional analyses (e.g., applying multidimensional gates) and the use of reference databases for e.g., comparing unknown cases against well-annotated, pre-existing cases [6,7,8,9]. However, building such databases is a daunting task, as strict quality assurance (QA) must be performed, in order to ensure that the enrolled cases are not affected by technical artefacts (e.g., instrument malfunction), deviations from standard sample preparation procedures (e.g., sample preparation mistakes) and/or to intra-laboratory variability (e.g., inter-instrument and inter-cytometrist variation) [1]. In other words, strict QA must be performed to ensure that the variation among cases truly and solely reflects biology.

Although various general-purpose flow cytometric QA strategies have been proposed previously [10,11,12], in our experience, these QA strategies cannot assure the level of quality required for diagnostic reference databases. Furthermore, to our best knowledge, no in-depth or hands-on information is publicly available about building diagnostic reference databases. Compared to other hematological malignancies, automating the diagnostic workflow for AML will likely be more complex, as AML is much more heterogeneous and complex in nature. Therefore, QA strategies that work for AML will likely also work for other diseases. We here report on the pitfalls and setbacks as encountered in building the EuroFlow AML database and provide an in-depth insight into our multidimensional QA strategy, based on BB and TS markers, applied to 1142 AML patient samples and 42 normal bone marrow (BM) samples. Despite standardized sample preparations and instrument settings (strict EuroFlow Standard Operating Procedures (SOP)), significant technical differences were still identified. Better understanding of such deviations will help to better organize the collection of datasets by avoiding such possible flaws upfront. Our approach to building the EuroFlow AML database may facilitate the building of other flow cytometric databases and may support or inspire the development of novel (semi)automated QA strategies.

## 2. Materials and Methods

### 2.1. Patients and Data

In total, flow cytometric data obtained from 1142 unique AML samples were included in this analysis. Data were collected in eight centers between 2010 and 2019; the institutional review board of each participating center approved this study. Participating centers were asked to upload anonymized data from patients fulfilling the following criteria: (1) primary diagnosis of AML based on local routine diagnostics, (2) classified according to the World Health Organization (WHO) classification, and (3) BM or peripheral blood (PB) evaluated using the EuroFlow AML panel (tube 1–6) according to EuroFlow sample preparation protocols and instrument settings [2,13,14].

### 2.2. Normal Bone Marrow Samples

Normal BM samples were obtained from (1) healthy donors, (2) patients without hematological or immunological diseases, (3) leukemia or lymphoma patients in long-term complete remission and negative for minimal residual disease, and (4) newly diagnosed lymphoma patients submitted for BM staging by flow cytometry and without evidence of disease. All samples were evaluated using the EuroFlow AML panel (tube 1–6) according to EuroFlow sample preparation and instrument settings SOP.

### 2.3. Data Collection and Evaluation

The overall workflow is schematically shown in Supplementary Data S1. For each AML case, the participating center was asked to upload the six anonymized FCS files (raw data from the six tubes of the EuroFlow AML panel). In addition, the laboratories performed a minimal analysis (removal of debris/doublets and identification of lymphocytes in the merged FCS file), purely based on the BB markers (allowing for a uniform analysis across each tube). The resulting CYT file (i.e., the Infinicyt analysis file) was uploaded to the secured server, as well (Supplementary Data S2). Naming conventions and analysis strategies were fully standardized (Supplementary Data S2). In addition, a spreadsheet with annotations (such as age, gender, WHO classification and instrument) had to be completed and submitted. Finally, each CYT file was checked by an independent reviewer (from another center) to make sure all cells were gated correctly.

Our QA strategy was based on mature lymphocytes, since lymphocytes (1) were present in virtually every sample, (2) could easily be distinguished from myeloid cells (based on the BB markers), and (3) were assumed to be unaffected in AML patients (as they originated from the lymphoid lineage).

## 3. Results and Discussion

### 3.1. Data Collection

In total, 803 unique AML cases were initially contributed by eight EuroFlow centers (Supplementary Data S2), and these were enrolled in the subsequent QA procedure. A detailed flowchart of the various stages and the resulting exclusions and/or flaggings is shown in Supplementary Data S1.

### 3.2. QA Stage 1: Initial Checks

In the first stage, various (automated) checks were performed (see Supplementary Data S3 for details). First, whether duplicate FCS files across cases were present (i.e., cases with identical data but with different identifiers) was evaluated through MD5 checksums. Four pairs of cases were identified that shared identical FCS files; these eight cases were flagged. Second, cases with more FCS files than expected were identified (e.g., tube 1 was acquired twice for unknown reasons); two of such cases were identified and flagged. Third, completeness of the FCS and CYT files was checked. For thirty-five cases, only the first four or five tubes were provided (i.e., tubes 5 and/or 6 were never acquired); for nine cases, any other FCS file was missing (e.g., tube 1 was missing); and for ten cases, no CYT file was provided (i.e., no analysis was performed by the originating center). These 54 cases were flagged. Fourth, whether the data within the CYT file matched the data within the corresponding FCS files was checked. In two cases, a mismatch was identified; these were flagged. In seven cases, the CYT file was incomplete (i.e., it only contained a subset of the FCS files). Fifth, whether the FCS files contained all required markers was checked. In thirteen cases, one (or more) markers were missing (presumably due to being out of stock and/or pipetting mistakes); these cases were flagged. Sixth, whether compensation matrices were present was evaluated; six cases had no compensation matrix, and these were flagged. Seventh and last, the number of lymphocytes per tube was evaluated. Since the lymphocytes were used for QA purposes, cases with less than 450 lymphocytes (an arbitrary cut-off) in any tube (*n* = 10) and cases with a large difference (>30%) in the number of lymphocytes among tubes (*n* = 2) were flagged.

Thus, in total 104 cases (13%) were flagged based on the aforementioned initial checks. The vast majority of these flaggings (73/104; 70%) were caused by human mistakes (i.e., problems related to analyzing and/or providing the appropriate files). In other words, it should be emphasized that the majority of flagged cases (so far) could potentially be fixed by correcting these human mistakes (as shown later). However, for this study, only the cases that passed all checks without any corrections and/or interventions (699/803; 87%) were enrolled into the following QA stage (sanitizations).

### 3.3. QA Stage 2: Sanitization of FCS Files

In the second stage, the FCS files were sanitized. Even though the EuroFlow protocols impose a strict naming convention, various deviations were identified, ranging from minor deviations (e.g., “HLA.DR” instead of “HLADR”) to major deviations (e.g., “[R]660/20” instead of “APC,” thus referencing channels by laser/filter instead of fluorochrome) (detailed examples in Supplementary Data S4). Even though the EuroFlow protocols strictly define the number of channels to be exported, various additional channels were found (e.g., SSC-W and FSC-W). Therefore, the FCS files were sanitized by forcing the EuroFlow naming convention (i.e., correct name for channels and markers) and forcing the EuroFlow structure (i.e., the required channels in the correct order). It should be emphasized that these modifications do not affect the data; however, they greatly simplify subsequent analysis. In the end, for each case (699/699; 100%), the files were successfully sanitized (i.e., made uniform).

### 3.4. QA Stage 3: Evaluation Based on Backbone Markers

**Figure 1:**
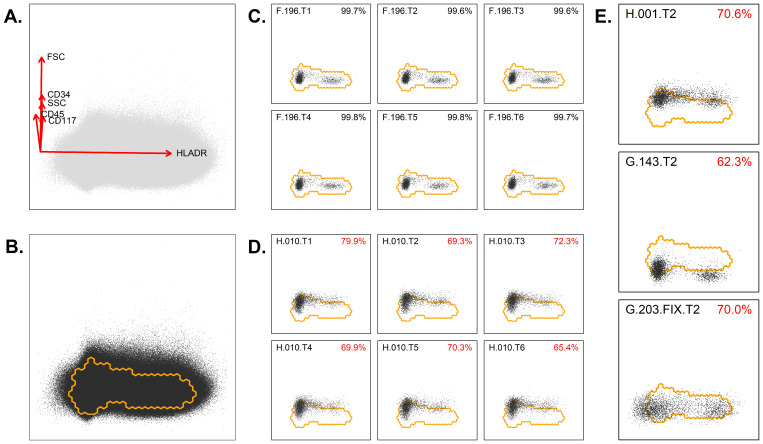
Evaluation of the backbone markers using PCA. (**A**) Lymphocytes were extracted from each FCS file (each tube from each patient), merged in a single FCS file and visualized in an automated population separator (APS) or PC1 vs. PC2 plot. The PCA of the merged lymphocytes was mainly composed of HLADR on the x-axis (PC1) and of CD34, CD117, CD45, and scatter characteristics on the y-axis (PC2). (**B**) The densest region, including 95% of events, was defined and marked as the lymphocyte reference region. (**C**) Example of a patient in whom the BB markers are appropriately located within the reference region for all six tubes. (**D**) Example of a patient in whom the BB markers were not appropriately located within the reference region for all six tubes. (**E**) Examples of three cases where part of the lymphocytes were outside the reference regions. Based on the principal components, as indicated in panel A, it can be deduced which marker is abnormally expressed. Upper panel: increased expression of CD45, CD117, or CD34. Middle panel: too low expression of CD45, CD117, or CD34, which might be related to compensation settings. Lower panel: abnormal expression for HLADR. The percentages indicate the number of cells within the reference region. Codes in upper left of plots refer to center, case number, and tube (T) number (e.g., F.196.T1 refers to center F, case 196, tube 1).

**Figure 2:**
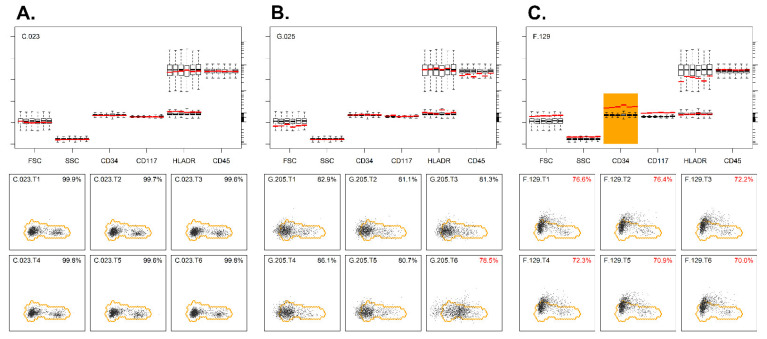
Evaluation of the backbone markers using PCA. (**A**) Example of a sample where the lymphocytes of all six tubes were well located within the reference region (lower panel). Median values of these lymphocytes (in red lines) were close to the median values of the reference cases (upper panel). (**B**) Example of a sample where lymphocytes of five out of six tubes were well located within the reference region (lower panel). The lymphocytes from tube 6 were, however, partly outside the reference region, and the direction of deviation was highly suggestive of abnormal HLADR expression. The median HLADR expressions of the patient’s lymphocytes (red lines) in tube 6 were clearly higher than the median values of the reference cases (upper panel). (**C**) Example of a sample where the lymphocytes of all six tubes are located outside the reference region (lower panel). Median CD34 expressions of the patient’s lymphocytes (red lines) were clearly above the median values of the reference cases, whereas the other BB markers showed normal expression (upper panel). The percentages indicate the number of cells within the reference region. Codes in the upper left part of plots refer to center, case number, and tube (T) number. Median values out of reference are highlighted in orange.

The checks, as performed in the third stage, were purely based on the BB markers, thereby allowing each tube to be handled in an identical way. First, for each tube, from each case, the lymphocytes (as identified via manual analysis) were exported to a separate FCS file, with the BB markers for up to 2000 events (total of 699 × 6 = 4194 files). Second, only cases with stable BB markers (across each tube) and bi-modal HLADR expression (within each tube) were considered suitable to establish the lymphocyte reference region (details and examples in Supplementary Data S5). These strict criteria resulted in a set of 384 cases; for these cases, the FCS files were merged into one FCS file (with BB data for a total of 4,394,241 lymphocytes). Third, dimensionality reduction was performed on the merged FCS file via Principal Component Analysis (PCA), and within the resulting PC1 vs. PC2 plane, the smallest region with 95% of the lymphocytes was identified (Figure 1A). This region was considered the lymphocyte reference region (thus, normal lymphocytes should mostly reside within this region). The parameters contributing to the PCA axes are given, as well (Figure 1B). Fourth, for each tube, from each case, the lymphocytes were projected in the same PC1 vs. PC2 plane, and the percentage of lymphocytes within the aforementioned lymphocyte reference region was calculated (Figure 1C,D) (details in Supplementary Data S6). Fifth, an overview was created, and cases with one (or more) tubes significantly out of reference (i.e., more than 20% of their lymphocytes out of reference) were flagged (Supplementary Data S7). Thus, in total, 125/699 cases were flagged (due to significant deviations from the reference region), and 574/699 were considered within reference (Figure 1E). Notably, for some cases (*n* = 46), all tubes were out of reference (e.g., due to an alternative BB fluorochrome being used), while for some other cases (*n* = 46) only one tube was out of reference (e.g., due to an abnormality or nonconformity within one tube). In the remaining cases (*n* = 33), several but not all tubes showed abnormalities (Supplementary Data S8). Interestingly, the deviations as identified via our median/peak-based approach (i.e., more or less traditional approach) were also found by our PCA-based approach (Figure 2 and Supplementary Data S8). Furthermore, our PCA-based approach also identified various abnormalities that were missed by the median/peak-based approach (Supplementary Data S8).

### 3.5. QA Stage 4: Evaluation Based on Tube-Specific (TS) Markers

**Figure 3:**
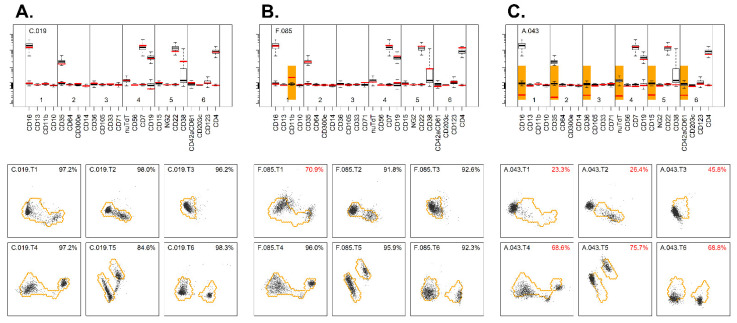
Evaluation of the tube-specific markers using PCA. (**A**) Example of a sample where expression of TS markers on lymphocytes was well within the reference for each tube (lower panel). Median expressions of TS markers of the patient’s lymphocytes (red lines) were close to the median values of the reference cases (upper panel). (**B**) Example of a sample where the expressions of TS markers on the lymphocytes of all but one tube were well located within the reference region (lower panel). The lymphocytes in tube 1 were, however, partly outside the reference region, and the direction is suggestive for abnormalities in CD11b expression. The median value of the patient’s lymphocytes (red lines) for CD11b in tube 1 were clearly higher than the median values of the reference cases (upper panel). (**C**) Example of a sample where the expressions of TS markers on the lymphocytes of all six tubes were located outside the reference region for one specific fluorescence channel (FITC) (lower panel). The median expressions of the FITC-conjugated markers (red lines) were clearly below the median expressions of the reference cases, which may indicate incorrect compensation settings (upper panel).

In the fourth stage, a similar analysis was performed, but now based on the TS markers. Consequently, the tubes could not be handled identically (as before), but they needed to be grouped by the EuroFlow AML tube number prior to merging (thereby matching the TS makers). First, for each tube, from each case, the lymphocytes were exported to a separate FCS file, resulting in six FCS files per case (one for each tube from the AML panel; each containing four unique TS markers), resulting in 574 × 6 = 3444 FCS files. Second, the FCS files were grouped by the AML tube number and merged into one FCS file (i.e., first tube merged for 574 cases, second tube merged for 574 cases, etc.) (Supplementary Data S9). Third, for each merged FCS file, dimensionality reduction was performed via PCA, and the smallest region with 95% of the lymphocytes was identified (Supplementary Data S10). Thus, for each EuroFlow AML tube, a specific reference was established. Fourth, each tube was checked against the appropriate reference (Figure 3), and cases with more than 20% of the lymphocytes out of reference were flagged (Supplementary Data S11). Fifth, an overview was created, and cases with one (or more) tubes out of reference were flagged. In total, 146 cases with too many lymphocytes outside the reference region (in at least one tube) were flagged, and 428/574 (75%) passed. A review of these cases revealed that for the majority of cases (*n* = 88; 60%), only one tube was out of reference (Supplementary Data S12). As before, the PCA-based approach and the mean/peak-based approach were highly concordant (Figure 3), although some minor differences in cases being flagged could be found (Supplementary Data S12). Thus, 428 cases out of the initial 803 cases (53%) passed every QA stage, whereas the remaining cases were flagged as being in need of additional evaluations and/or adjustments (see Section 3.6).

Obviously, using lymphocytes as controls for each marker is not optimal, as various markers of the AML panel are not expressed on lymphocytes. However, lymphocytes essentially formed the most consistent (i.e., always present), distinct (i.e., unambiguous identification based on the BB markers), and secure (i.e., belonging to the non-affected lineage, the lymphoid lineage) internal control at hand. Other candidates (e.g., eosinophils and neutrophils) belonged to the affected lineage (i.e., myeloid lineage) and could not in all cases reliably be separated from the leukemic cells (based on the BB markers). Therefore, we believe that lymphocytes are well suited for QA analysis of these cases. It should be emphasized that highly similar reference regions were obtained in case the reference regions were based on maximally 30 cases per center (thereby ruling out the potential overrepresentation of centers that submitted large numbers of cases).

### 3.6. Detailed Analysis of Flagged Cases

Despite the highly standardized sample preparations and instrument settings (EuroFlow SOP), important technical differences were still noted in a considerable number of cases, which were flagged because of lymphocytes being out of the reference region. To better understand these deviations, we evaluated possible causes in more detail (Supplementary Data S13). Issues with the BB markers were predominantly observed in centers G and H. Center H used CD45-V500 and generally had a too-high mean fluorescence intensity (MFI) for this marker on the lymphocytes. It should be noted that EuroFlow solely recommends CD45-V500c as an alternative reagent (for the reference reagent CD45-PO), since only CD45-V500c is known to result in similar MFI values. Thus, using CD45-V500 was not in line with the SOP and directly resulted in cases to be flagged. In center G, using CD45-PO and, later, CD45-OC515, several cases were flagged due to CD45 being out of reference. Since these deviations were consistently found in subsequent cases, this most likely reflects differences in antibody lots or temporary changes in the instrument set-up. Obviously, appropriate acceptance testing of new antibody lots and rigorous checks after instrument changes are crucial for producing reproducible and consistent data (over time and between centers). Furthermore, several cases from center G were flagged because tube 4 was out of reference; this particularly concerned an increased FSC and slightly higher background intensities of CD34 and CD117 on the lymphocytes. We could not identify any particular cause of these differences and consider these to be minor issues that will not or will only very limitedly impact any subsequent analysis.

With respect to the cases flagged for the TS markers, consistent compensation issues were detected for center A, resulting in abnormal FITC values in all tubes (more details in Section 3.7). Most flagged cases in other centers were flagged based on tube 4 or 6, which are the tubes containing typical lymphoid markers (such as CD7, CD19, CD56, and CD4) and therefore may be expected to be the most sensitive for detecting staining abnormalities on lymphocytes. For tube 4, a higher expression of TdT was also observed in some samples; in most centers, lymphocytes present in these samples still were clearly TdT-negative (MFI < 1000) but in center H MFI levels frequently exceeded 1000. Center H used the reference reagent in the right volume and followed the EuroFlow SOP for staining; FITC signals in the other tubes were normal, so it remains unclear what caused these higher background levels. Center F had relatively more abnormalities in tube 6, with slightly increased background levels of CD123 on lymphocytes. Re-evaluation indicated that this mainly was due to undercompensation of CD4-APCH7 and/or degradation of CD4-APCH7. In some other cases from the same center, CD42a.CD61 was out of reference (slightly negative values instead of around zero) despite appropriate compensation. These cases had significantly higher thrombocyte counts in blood as compared to cases with normal CD42a.CD61 staining; this likely is due to software calculations in which the fluorescence values of events that are below the (FSC) threshold are used to remove the background signal of the acquired events (above the threshold). If many thrombocytes are present (below the threshold), their positive CD42a.CD61 signal will be used for correcting the lymphocyte values, resulting in signals below zero.

Our data show that if people concisely follow the SOP, uniform and reproducible data can generally be obtained, but special attention should be paid to compensation settings (with regular control of appropriate settings using single stained or normal samples). Furthermore, despite standardization, several deviations were identified; some of these can be fixed afterwards (e.g., compensation, nomenclature), but others are irreversible (e.g., use of other reagents, missing markers). Similar issues can be expected in building databases for other diseases than AML and in other multicenter flowcytometric studies (especially studies over time, across centers, and across instruments). Our data may raise more awareness for problems that occur during these kinds of long-term data collection efforts and may help others to prevent similar problems in the construction of such datasets.

### 3.7. Fixing of Sub-Optimal or Incomplete Cases

For part of the 375 flagged cases, relatively easy fixes could be performed (Supplementary Data S14). For 43 cases, one obvious compensation issue was identified (i.e., one value in the matrix was set wrong); after correction (applying the median compensation value of the non-flagged cases from the same center), 37/43 cases passed every QA stage. For the ten cases without analysis (no CYT file) and the seven cases with an incomplete analysis (incomplete CYT file), analysis was performed (new CYT file created), and 7/17 cases subsequently passed all QA stages. Presumably, the relatively high percentages of cases failing the QA may be explained by the fact that the contributing center already noticed that the data were suboptimal and therefore did not include a (complete) CYT file. Thus, by performing easy and obvious fixes, already, 44 out of 375 cases could be un-flagged, resulting in a total of 472 suitable cases. Presumably, several of the remaining flagged cases may still be useful, but these cases need more detailed review (i.e., anything other than correcting obvious human mistakes).

### 3.8. Final Cohort

As shown in Supplementary Data S15, the data from these 472 cases showed highly comparable staining patterns on lymphocytes, also across the eight participating centers. The only exception was CD38, which showed different expression patterns between the participating centers. This can, however, be explained since maturation within the naive mature B-cell subset is characterized by a decrease in CD38 expression and an increase in the number of passed replication cycles [15]. Indeed, lower CD38 expression levels were observed in the centers that mainly included adult patients, whereas CD38 expression was generally higher in centers including mainly pediatric patients (Supplementary Data S15).

### 3.9. QA Process Validation

In order to validate the QA procedure, 339 cases were randomly picked from the initial cohort (*n* = 803), and their raw FCS files were re-analyzed from scratch (i.e., their lymphocytes were manually gated again, and the resulting CYT files went through the aforementioned QA stages, using the previously defined reference regions). Conclusions were identical for all cases (i.e., in both runs, the same 259 cases passed all QA stages successfully, and the same 80 cases were flagged) (details in Supplementary Data S16). This shows that the presented QA procedure is robust and reproducible.

As a second technical validation, we used 339 new cases, more recently acquired using the EuroFlow AML panel, from which FCS files and CYT files (but no annotations) were provided by the participating centers. These cases were also processed according to the aforementioned QA procedure. All cases passed the initial checks, 315 cases (93%) passed the BB checks, and 225 cases (67%) also passed the TS checks. The percentage of complete/optimal cases (67%) is higher than the 54% from the initial cohort. This is likely due to the increased experience of the involved EuroFlow centers, sharing experiences and problems during regular meetings, improvements in the SOP, and improved adherence to the SOP.

It should be noted that the entire QA procedure was performed in an R statistical environment. However, nowadays, fairly comparable QA procedures can be performed in flow cytometry software, such as Infinicyt. For example, within Infinicyt, reference images (essentially reference regions) can be made based on the two standard deviations of the lymphocyte populations, and the percentage of cells outside such reference images can be obtained. The pipeline as used for this work relies on non-public dependencies (from EuroFlow) and was mostly created with the AML panel in mind; therefore, this pipeline cannot be used as a general-purpose pipeline. Nevertheless, the same principle holds true for any other panel.

### 3.10. Annotations

Obviously, annotations are crucial for detailed evaluations (e.g., correlations between immunophenotypes and WHO classification). Even though centers were requested to exclusively upload cases with a complete set of annotations, unfortunately, some cases were uploaded without annotations. Of the 472 cases that passed the QA procedure (428 directly and 44 after minor fixes), ultimately, no complete annotations were available for 34 cases (7%). Most of these cases were clearly AML cases, but their final WHO classification could not be established due to missing data (e.g., for external patients referenced to the participating centers). In addition, 21 cases (4%) appeared to be MDS-excess of blasts or MPAL, and 16 cases (3%) turned out to be relapses. These 71 cases were excluded from the final dataset, resulting in 401 cases at diagnosis with high-quality flowcytometric data and annotations available. The characteristics of these 401 patients are shown in Table 1.

### 3.11. Data Analysis of AML Cohort: Immunophenotypic Profiles

**Figure 4:**
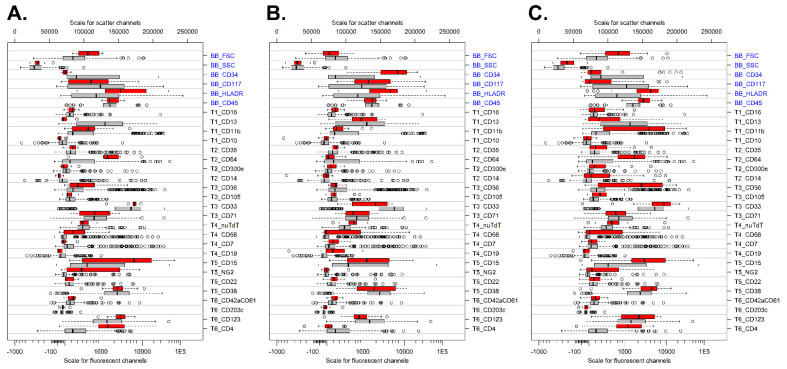
Immunophenotypic profile of specific WHO categories. (**A**) Comparison of t(9;11) cases (red; *n* = 9) versus all other AML cases (gray; *n* = 392). (**B**) Patients with t(8;21)-positive AML (red; *n* = 16) versus all other AML cases (gray; *n* = 385). (**C**) Patients with monoblastic/monocytic AML (red; *n* = 30) versus all other AML cases (gray; *n* = 371). For each marker, the median fluorescent intensity is shown. The boxes themselves represent the interquartile range (first up to third quartile), and the vertical bars within the boxes represent the median. Data were sorted from top to bottom based on the delta median for the red group (selected cases) and the gray group (all other AML cases). Backbone (BB) markers are shown in blue, and T1-T6 refer to markers present in tube 1 to tube 6.

To confirm the correctness of the established cohort (*n* = 401), we evaluated several WHO categories for markers previously reported to be differently expressed [16]. Comparison of t(9;11) cases versus all other AML cases showed strong expressions of CD15, CD4, CD64, NG2, and HLADR and low expressions of CD34 and CD13 in t(9;11) cases (Figure 4A). Patients with t(8;21)+ AML showed high expressions of CD34, HLA-DR, and CD15 but lower expression of CD33 as compared to other AML subtypes (Figure 4B). CD19 was generally weakly expressed by the t(8;21) cases, which may be related to the applied clone and/or fluorochrome (APC-H7) [16]. Finally, patients with monoblastic/monocytic AML had increased expressions of CD11b, CD64, CD36, HLADR, CD4, and CD15 but reduced expressions of CD34, CD13, and CD117 as compared to other AML subtypes (Figure 4C). Overall, these data show that the dataset created reliably detects immunophenotypic patterns in AML and therefore can further be used for exploring the immunophenotypic variability of AML.

### 3.12. Inclusion of Normal Bone Marrow Samples

In addition to AML cases, we also included normal BM samples stained and acquired using the same EuroFlow sample preparation and instrument settings SOP. These cases provide a frame of reference for normal myeloid development and will aid in the identification of cells with an abnormal immunophenotype. Data from 42 normal BM samples were uploaded to the EuroFlow server by five different centers and processed with the same QA as described above for the AML cases. One case was excluded due to the absence of HLADR in one of the tubes, seven cases were flagged based on the BB markers, and twelve were flagged based on the TS markers. In the end, 22 cases passed the QA without any intervention. Six of the flagged cases could easily be fixed by adjusting one compensation parameter (similarly to the AML patients). Therefore, finally, 28 normal BM samples (67%) were included in our dataset (details in Supplementary Data S17). This analysis also confirms that lymphocytes in AML patients are similar to normal lymphocytes and thus can reliably be used for QA purposes.

### 3.13. Strictness of the QA

During the QA, almost half of the initially submitted cases (375/803) were flagged. About one-third of the flagged cases were flagged in the initial phase: 35 cases were uploaded incompletely (e.g., missing files, missing analysis, missing compensation, etc.), and 66 cases had technical issues (e.g., duplicates files, missing markers, etc.). Obviously, parts of these cases should not have been uploaded in the first place, as they did not fulfil the prerequisites. Other cases were excluded due to obvious human error (e.g., duplicate or mismatched data), again proving that such checks are crucial and that data handling should be automated as much as possible. The remaining two-thirds of flagged cases were due to lymphocytes being partly out of reference, either based on the BB or TS markers. It should be stressed that these cases should not be excluded but that they are flagged for further evaluation. The observed deviations clearly indicate differences compared to the average of the cohort, but this does not necessarily imply that these cases cannot be used altogether. For example, higher background levels of CD34 on lymphocytes may indicate issues with the staining but may not have any major impact on the analysis of the leukemic cells.

The strictness of this QA can be easily adjusted, for example, by changing the reference region size (e.g., from 95 to 90%) and/or by changing the cutoff for flagging cases (e.g., from 20 to 10%). Unfortunately, there is no golden standard to define what deviation is acceptable or not; however, the here-reported QA procedure essentially identifies those cases that differ most from the cohort average (as seen in the PC1 versus PC2 graphical representation). In other words, cases are excluded by relative measures (i.e., being on the extremes of the cohort) and not by absolute measures (i.e., failing to meet any fixed quality criteria). Interestingly, now that the AML cohort has been established using our QA procedure (i.e., the cases that most deviated from the cohort average were excluded), the AML cohort can be easily extended based on identical quality criteria (i.e., just by comparing new cases against the already-established reference). In other words, during the first establishment, cases were excluded based on relative measures (i.e., deviation from the cohort average), and now cases can be included by absolute measures (i.e., the fixed reference, which is not influenced by new cases).

The strictness of the QA procedure can be adjusted by changing the cutoff percentages (as used in the third and fourth QA stages) and mostly depends on two factors: firstly, the quality of the cohort itself (e.g., low-quality cohorts require more exclusions, while high-quality cohorts require less exclusions); this setting can be optimized by finding the “natural break” in the cohort (Supplementary Data S7) and, secondly, the subsequent evaluations that one has in mind (e.g., tSNE relies on continuous measures and therefore requires higher-quality input data as compared to simple nominal/binary evaluation).

### 3.14. Applicability of the QA Procedure

In this study, we used an AML cohort for constructing a database. The fact that the AML panel consisted of six tubes, as well as the heterogeneity both within and between AML patients, gave extra complexity to the QA process. One could argue that the recent development of spectral flow cytometers will significantly reduce the number of tubes needed and that an approach with merged tubes will become outdated. However, for building databases for use in a clinical setting, strict QA will remain critical, and QA approaches, such as applied for AML patients, can also be used for other cohorts with just one or a few tubes. If single tubes with a high number of antibodies (e.g., >20) are being used, one could first perform a bulk cleanup based on a limited number of well-known antibodies (comparable to our BB markers), followed by a more detailed QA of the remaining markers.

## 4. Conclusions

Our data show that obtaining and collecting reproducible flow cytometric data over time and across centers is feasible but also that strict QA remains crucial, even when standard SOPs for staining and instrument settings are being used in a multicenter setting. This work provides a concrete and unique QA strategy for multi-tube flow cytometric assays that rely on the BB/TS paradigm but can also be used for other high-dimensional (single-tube) flow cytometric datasets. This approach can be added to the existing procedures, and it can be complemented with other QA tools, such as the R package FlowAI (evaluating flow rate abnormalities out of dynamic range expression signals and parameter stability) [17]; the R package FlowCore (e.g., exclusion of extreme values in scatter parameter and doublets) [18]; and/or PeacoQC (e.g., checking signal stability over time and selecting the high-quality intervals and discarding the inferior quality measurements) [4].

## Figures and Tables

**Table 1:** Patient characteristics (final cohort, *n* = 401).

Parameter	Final Cohort (*n* = 401)
Gender (M/F)	217/184
Age (years; median [range])	53 [0–93]
WBC (x10^9^/L; median [range])	15 [0–441]
WHO classification	
Recurrent genetic abnormalities	
•t(8;21)	16
•Inv(16)/t(16;16)	21
•t(15;17)	23
•t(9;11)	9
•t(6;9)	2
•inv(3)/t(3;3)	2
•NPM1	109 ^a^
•Biallelic CEBPA	13 ^a^
•RUNX1	4
Myelodysplasia-related changes	41
Therapy-related	21
Not otherwise specified	137
•Minimal differentiation	14
•Without maturation	25
•With maturation	32
•Myelomonocytic	22
•Monoblastic/monocytic	30
•Pure erythroid	7
•Megakaryoblastic	7
Associated with Down Syndrome	5
•TAM/DS-ML	5

^a^ Two patients were both NPM1+ and CEBPA+.

## Data Availability

For original data, please contact the corresponding author.

## Supplementary Materials

The following supporting information can be downloaded at https://www.mdpi.com/article/10.3390/cancers14082011/s1, Supplementary Data S1: Workflow Overview; Supplementary Data S2: Data Collection; Supplementary Data S3: Initial Checks; Supplementary Data S4: Sanitization; Supplementary Data S5: Establish BB Reference Data; Supplementary Data S6: Establish BB Reference Region; Supplementary Data S7: Check cases against BB Reference Region; Supplementary Data S8: Review of marked cases (BB); Supplementary Data S9: Establish TS Reference Data; Supplementary Data S10: Establish TS Reference Region; Supplementary Data S11: Check cases against TS Reference Region; Supplementary Data S12: Review of marked cases (TS); Supplementary Data S13: Details of technically flagged cases; Supplementary Data S14: Fixes; Supplementary Data S15: Final Results; Supplementary Data S16: Pipeline Validation; Supplementary Data S17: Normal BM samples.

## References

[1] Pedreira C.E., da Costa E.S., Lecrevise Q., Grigore G., Fluxa R., Verde J., Hernandez J., van Dongen J.J.M., Orfao A. (2019). EuroFlow. From big flow cytometry datasets to smart diagnostic strategies: The EuroFlow approach. J. Immunol. Methods.

[2] Van Dongen J.J., Lhermitte L., Bottcher S., Almeida J., van der Velden V.H., Flores-Montero J., Rawstron A., Asnafi V., Lecrevisse Q., Lucio P. (2012). EuroFlow antibody panels for standardized n-dimensional flow cytometric immunophenotyping of normal, reactive and malignant leukocytes. Leukemia.

[3] Duetz C., Van Gassen S., Westers T.M., van Spronsen M.F., Bachas C., Saeys Y., van de Loosdrecht A.A. (2021). Computational flow cytometry as a diagnostic tool in suspected-myelodysplastic syndromes. Cytom. Part A.

[4] Quintelier K., Couckuyt A., Emmaneel A., Aerts J., Saeys Y., Van Gassen S. (2021). Analyzing high-dimensional cytometry data using FlowSOM. Nat. Protoc..

[5] Van Gassen S., Callebaut B., Van Helden M.J., Lambrecht B.N., Demeester P., Dhaene T., Saeys Y. (2015). FlowSOM: Using self-organizing maps for visualization and interpretation of cytometry data. Cytom. A.

[6] Flores-Montero J., Grigore G., Fluxá R., Hernández J., Fernandez P., Almeida J., Muñoz N., Böttcher S., Sedek L., van der Velden V. (2019). EuroFlow Lymphoid Screening Tube (LST) data base for automated identification of blood lymphocyte subsets. J. Immunol. Methods.

[7] Flores-Montero J., Sanoja-Flores L., Paiva B., Puig N., Garcia-Sanchez O., Bottcher S., van der Velden V.H., Perez-Moran J.J., Vidriales M.B., Garcia-Sanz R. (2017). Next Generation Flow for highly sensitive and standardized detection of minimal re-sidual disease in multiple myeloma. Leukemia.

[8] Lhermitte L., Barreau S., Morf D., Fernandez P., Grigore G., Barrena S., de Bie M., Flores-Montero J., Brüggemann M., Mejstrikova E. (2021). Automated identification of leukocyte subsets improves standardization of database-guided expert-supervised diagnostic orientation in acute leukemia: A EuroFlow study. Mod. Pathol..

[9] Lhermitte L., Mejstrikova E., van der Sluijs-Gelling A.J., Grigore G.E., Sedek L., Bras A.E., Gaipa G., da Costa E.S., Novakova M., Sonneveld E. (2018). Automated database-guided expert-supervised orientation for immunophenotypic diagnosis and classification of acute leukemia. Leukemia.

[10] Johansson U., Bloxham D., Couzens S., Jesson J., Morilla R., Erber W., Macey M. (2014). British Committee for Standards in Haematology Guidelines on the use of multicolour flow cytometry in the diagnosis of haematological neoplasms. Br. J. Haematol..

[11] Greig B. (2019). Quality Control of Immunophenotyping.

[12] Wood B.L., Arroz M., Barnett D., DiGiuseppe J., Greig B., Kussick S.J., Oldaker T., Shenkin M., Stone E., Wallace P. (2007). 2006 Bethesda International Consensus recommendations on the immunophenotypic analysis of hematolymphoid neoplasia by flow cytometry: Optimal reagents and reporting for the flow cytometric diagnosis of hematopoietic neoplasia. Cytom. Part B Clin. Cytom..

[13] Kalina T., Flores-Montero J., Lecrevisse Q., Pedreira C.E., Van Der Velden V.H.J., Novakova M., Mejstrikova E., Hrusak O., Böttcher S., Karsch D. (2015). Quality assessment program for EuroFlow protocols: Summary results of four-year (2010–2013) quality assurance rounds. Cytom. A.

[14] Kalina T., Flores-Montero J., van der Velden V.H.J., Martin-Ayuso M., Böttcher S., Ritgen M., Almeida J., Lhermitte L., Asnafi V., Mendonça A. (2012). EuroFlow standardization of flow cytometer instrument settings and immunophenotyping protocols. Leukemia.

[15] Theunissen P.M.J., Branden A.V.D., Van Der Sluijs-Gelling A., De Haas V., Beishuizen A., Van Dongen J., Van Der Velden V.H.J. (2017). Understanding the reconstitution of the B-cell compartment in bone marrow and blood after treatment for B-cell precursor acute lymphoblastic leukaemia. Br. J. Haematol..

[16] Hrušák O., Porwit-MacDonald A. (2002). Antigen expression patterns reflecting genotype of acute leukemias. Leukemia.

[17] Monaco G., Chen H., Poidinger M., Chen J., de Magalhaes J.P., Larbi A. (2016). flowAI: Automatic and interactive anomaly discern-ing tools for flow cytometry data. Bioinformatics.

[18] Hahne F., LeMeur N., Brinkman R.R., Ellis B., Haaland P., Sarkar D., Spidlen J., Strain E., Gentleman R. (2009). flowCore: A Biocon-ductor package for high throughput flow cytometry. BMC Bioinform..

