# Supplementary material for: Quality Assessment of a Large Multi-Center Flow Cytometric Dataset of Acute Myeloid Leukemia Patients—A EuroFlow Study
Source: Cancers (Basel). 2022 Apr 15;14(8):2011. doi: 10.3390/cancers14082011 (PMC9033003; doi:10.3390/cancers14082011)
Supplement: Supplementary file 1 [file cancers-14-02011-s001.zip › cancers-1681549-supplementary.pdf]

Supplementary Data S1 – Workflow Overview

Overview of the Quality Assurance (QA) procedure:

**QA steps in chronological order**		**Count**	**Supplement**
Data Collection			
Initiation:	Request laboratories to upload cases	NA	02
Input :	Cases as uploaded by the laboratories	+ 803	02
Initial Checks			
Flagging :	Duplicate FCS files across cases	- 8	03
Flagging:	Ambiguous FCS files within cases	- 2	03
Flagging:	Incomplete panel (only subset)	- 35	03
Flagging:	Incomplete panel (tube missing)	- 9	03
Flagging:	Analysis missing (no CYT file)	- 10	03
Flagging:	Analysis mismatch (FCS vs. CYT files)	- 2	03
Flagging:	Incomplete merge (incomplete CYT file)	- 7	03
Flagging:	Missing Markers	- 13	03
Flagging:	Missing compensation	- 6	03
Flagging:	Too few lymphocytes	- 12	03
Sanitization of FCS files			
Synchronize :	Channel ordination	NA	04
Synchronize:	Naming conventions	NA	04
Evaluation based on backbone (BB) markers			
Establish :	Reference for BB markers	NA	05
Establish :	Reference region for BB markers	NA	06
Flagging:	Cases out-of-reference	- 125	07
Review :	Flagged cases	NA	08
Review :	Remainder of cases	NA	
Evaluation based on tube-specific (TS) markers			
Establish :	Reference for TS markers	NA	09
Establish :	Reference region for TS markers	NA	10
Flagging:	Cases out-of-reference	- 146	11
Review :	Flagged cases	NA	12+13
Finalization			
Apply :	Fixes of sub-optimal or incomplete cases	+ 44	14
Output :	Final cohort	428	15
Perform :	QA process validation		16
Normal BM samples			17

Supplementary Data S2 – Data Collection

EuroFlow laboratories were requested to contribute consecutive AML cases, meeting the following criteria:

- Availability of WHO classification (and various other annotations)
- Availability of immunophenotypic data, meeting the following criteria:
    - Bone marrow (BM) or peripheral blood (PB) acquired at initial diagnosis;
    - Staining and acquisition according to the EuroFlow protocols;
    - EuroFlow AML panel, at least tube 1 up to 6, optionally tube 7 (See Table below);
    - Minimal analysis, based on BB markers (at least gating of lymphocytes and exclusion of debris/doublets) using a common profile (see below).

Table S2. 1. Composition of the EuroFlow AML/MDS panel.

Tube	FITC	PE	PerCP-Cy5.5	PE-Cy7	APC	APC-H7	PB	PO
1	CD16	CD13			CD11b	CD10		
2	CD35	CD64			CD300e	CD14		
3	CD36	CD105			CD33	CD71		
4	nuTdT	CD56	CD34	CD117	CD7	CD19	HLADR	CD45
5	CD15	NG2			CD22	CD38		
6	CD42a+CD61	CD203c			CD123	CD4		
7	CD41	CD25			CD42b	CD9		

The following analysis profile (Infinicyte .inp file) was provided:

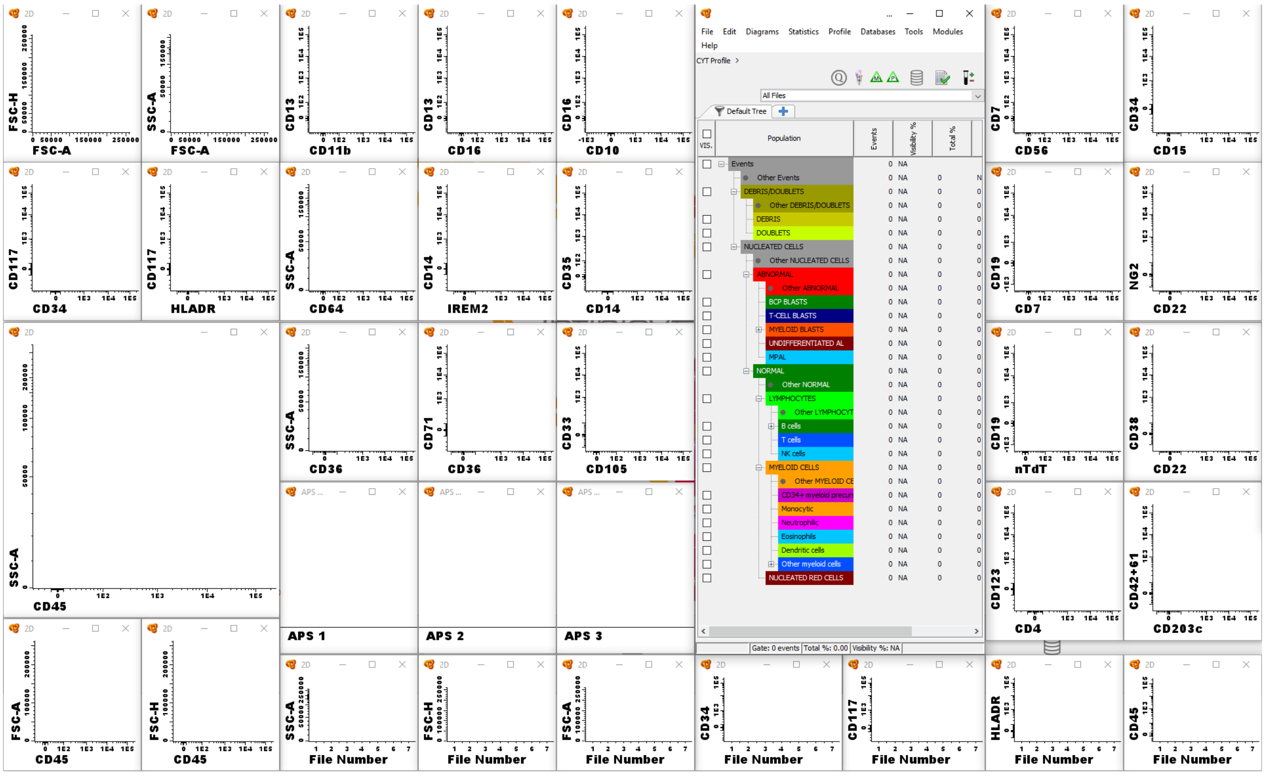

Plot arrangement (essentially “by BB markers” and “by tube”):

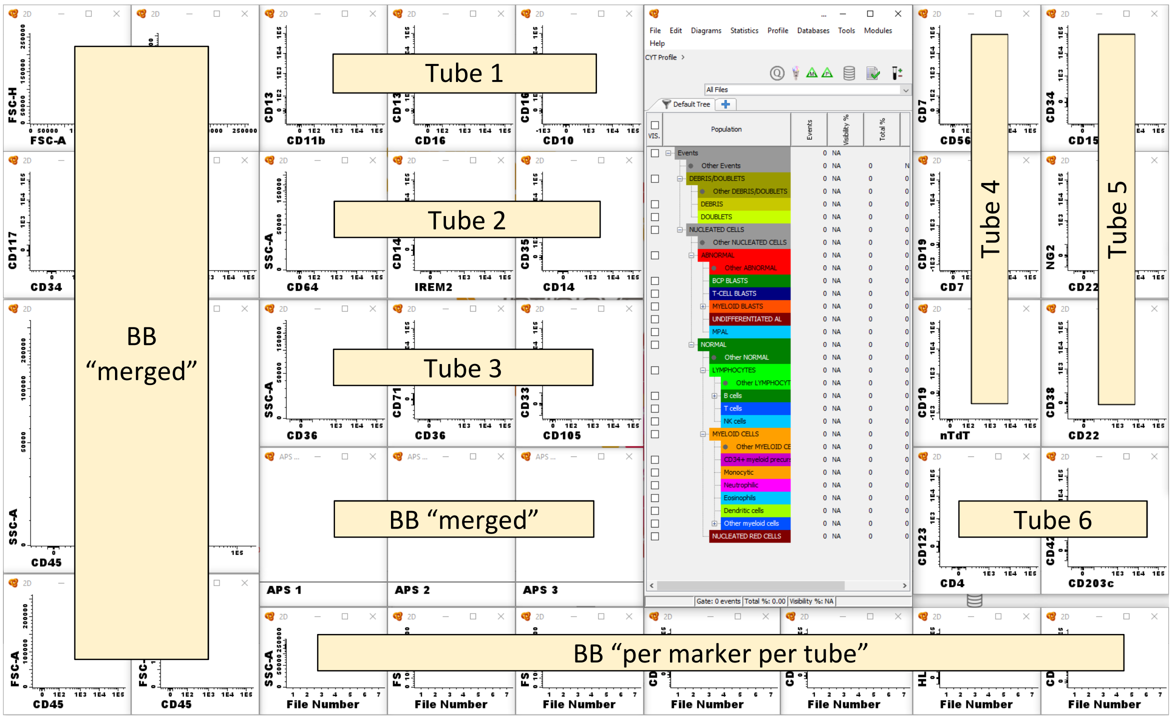

Plot axis (markers on X and Y axis):

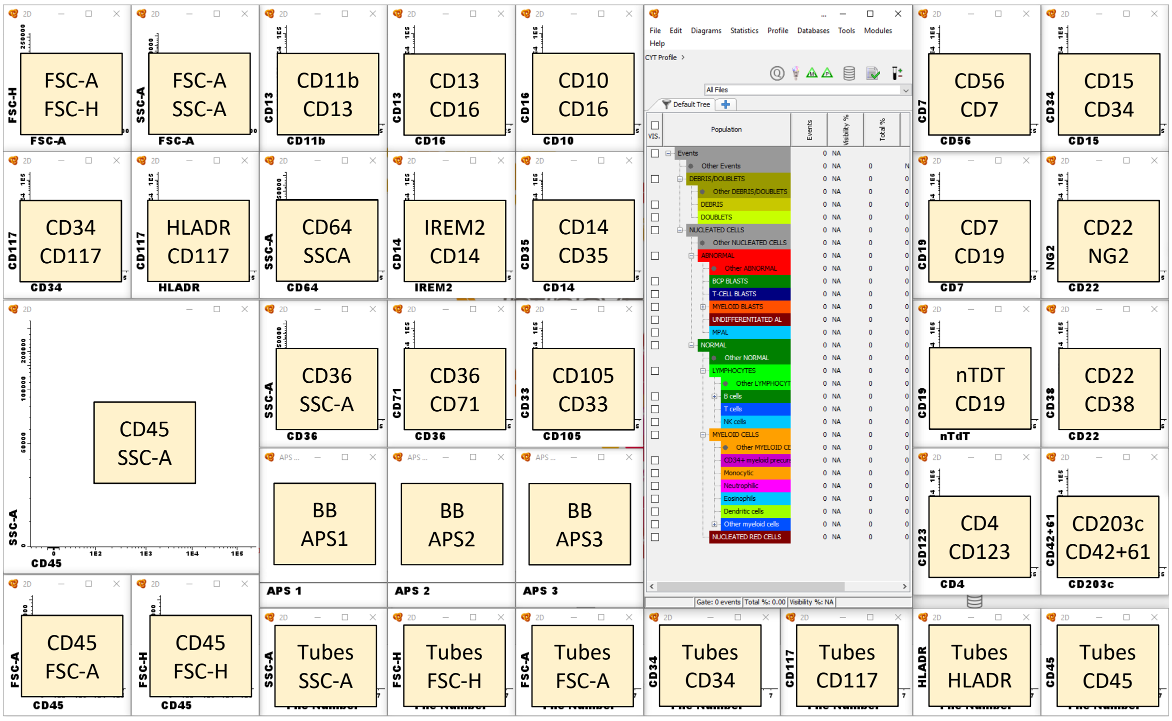

Laboratories gated (at least) the lymphocytes:

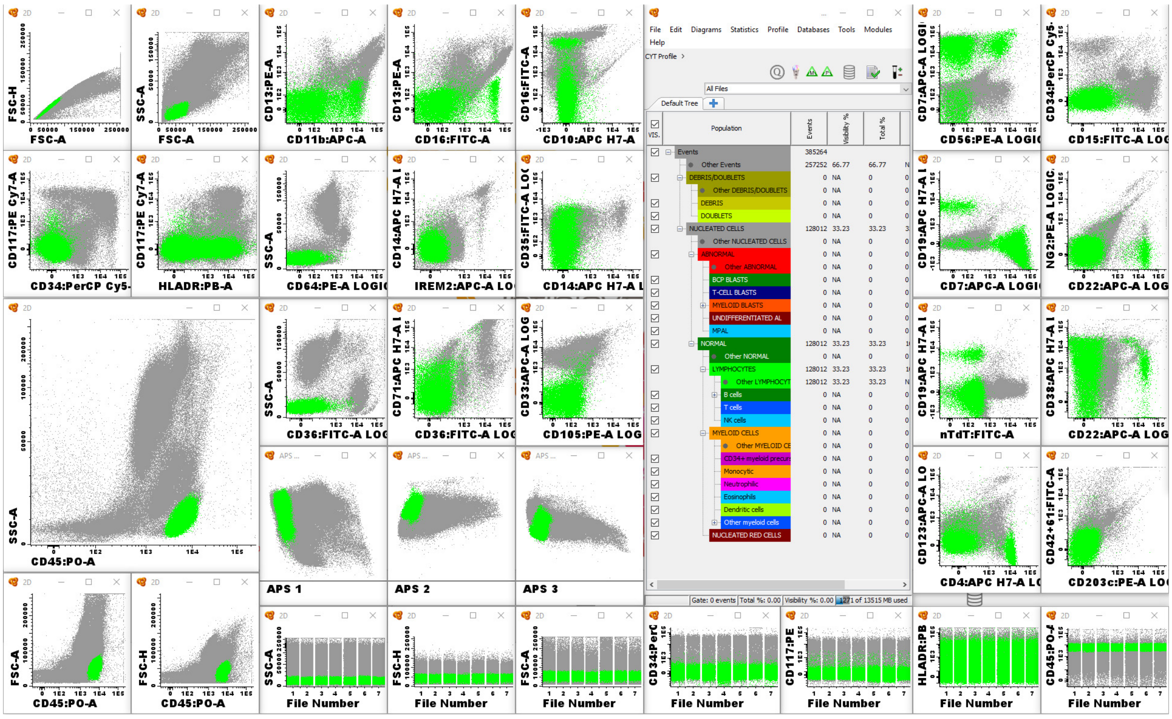

Optionally, laboratories (optionally) gated the myeloid populations (normal cells and AML cells):

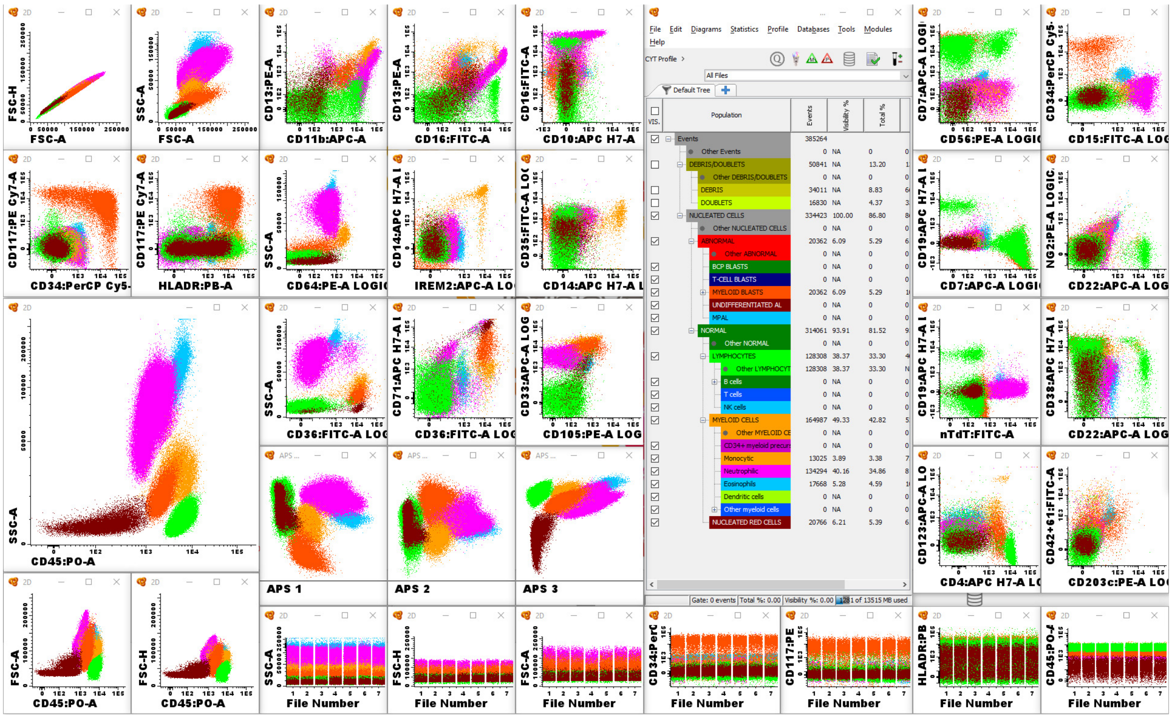

Nevertheless, for the QA procedure, only the lymphocyte gating information was used.

The lymphocytes (and optionally myeloid populations) were only gated based on the merged BB plots:

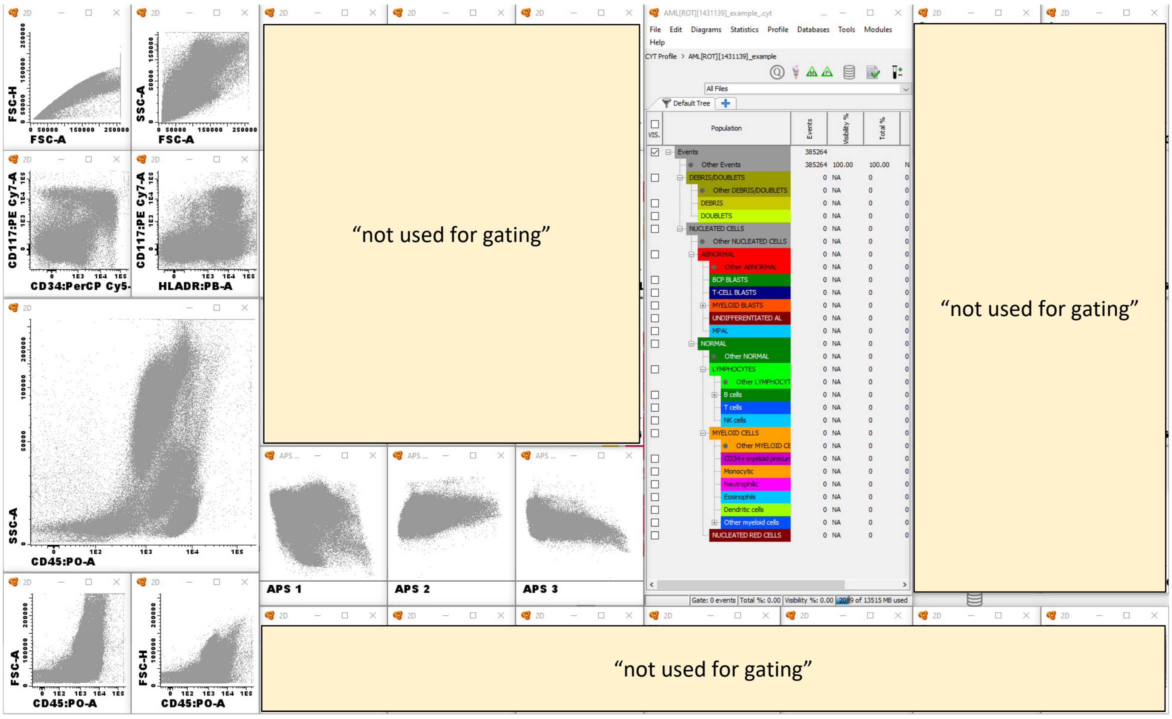

By gating based on the merged BB plots, by definition, the same gates were applied to each tube. For example, by drawing the purple gate within the merged BB plot for CD45 versus SSC-A, essentially, the same gate was applied to tube 1 up to 6 from the AML/MDS panel (individual tubes shown in blue):

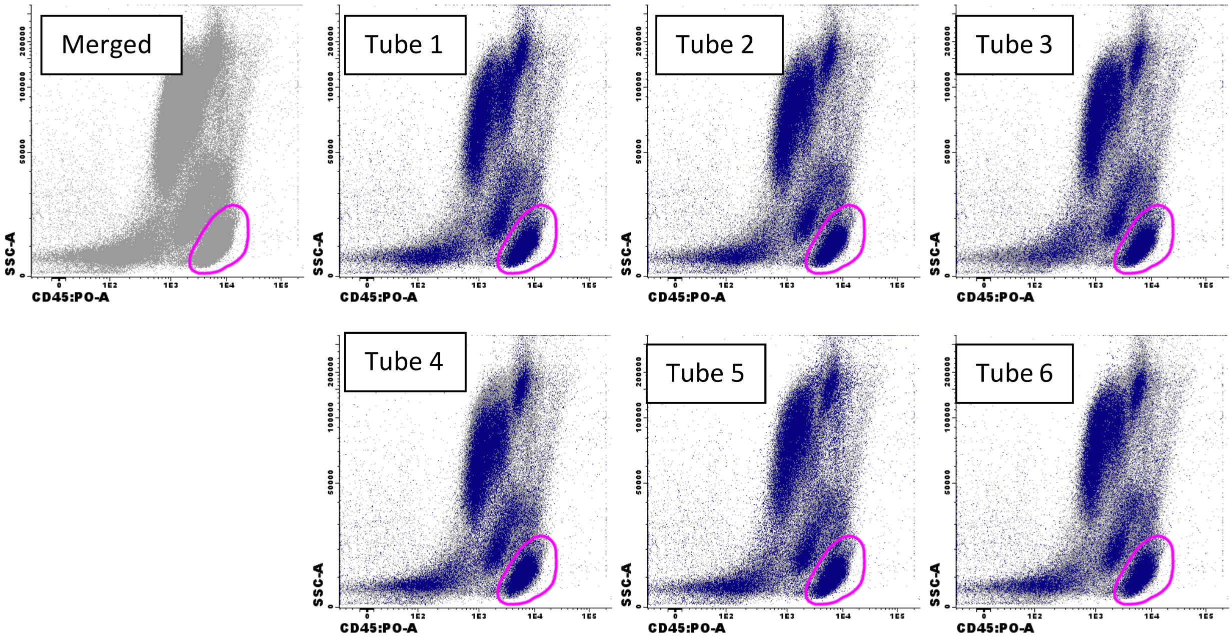

Except for one case (without lymphocytes), lymphocytes could always be gated without using arbitrary gates, based on the BB makers (FSC-A, SSC-A, CD34, CD117, HLADR and CD45). In other words, just given the BB makers, the lymphocytes (shown in green) always featured a distinct immunophenotype (as compared to the other cells) and always could be gated with ease. For example, cases with full separation based on CD45 versus SSC-A:

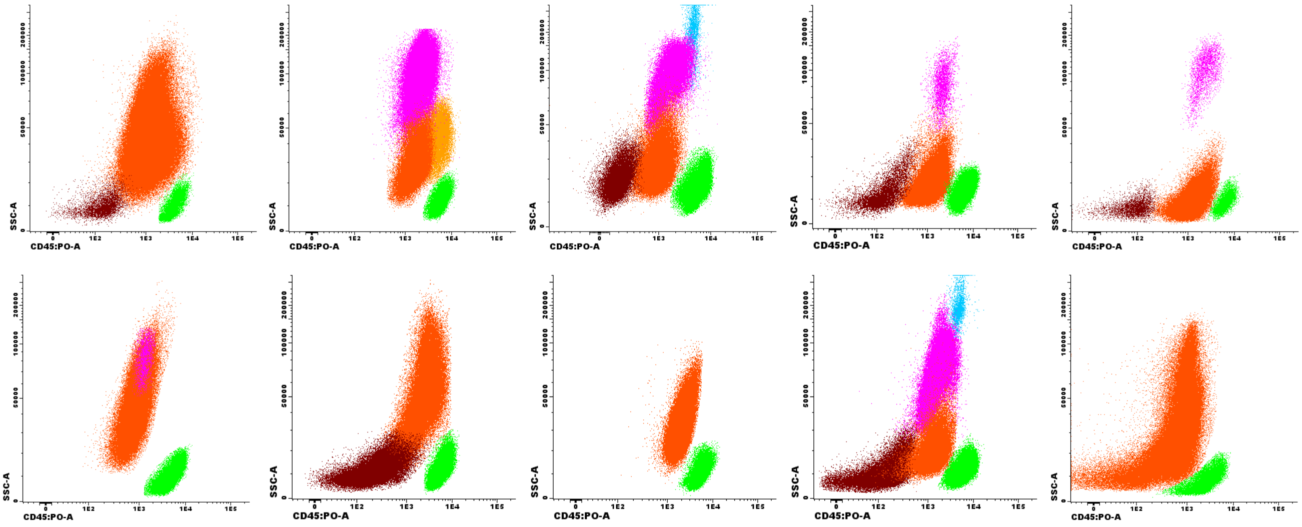

For example, cases with full separation based on FSC-A versus SSC-A:

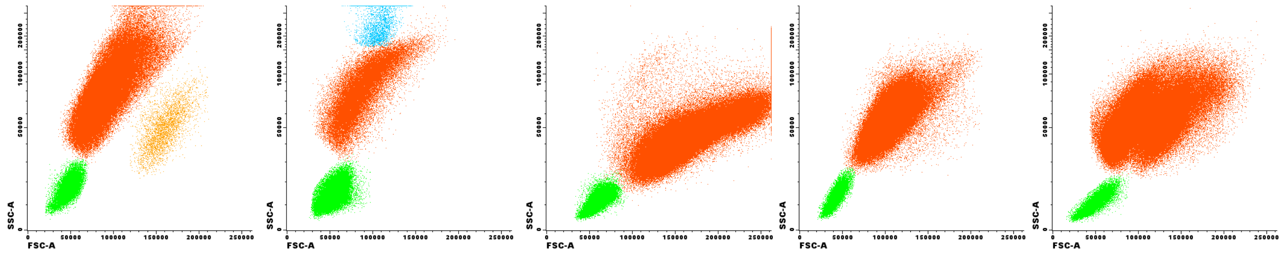

For each case, the FCS files (as produced by the flow cytometer) and the CYT file (containing the manual analysis) were provided by the participating laboratories, according to the following naming convention:

- AML[<LAB>][<CASE NUMBER>].CYT = Infinicyt analysis for each tube (in merged fashion)
- AML[<LAB>][<CASE NUMBER>][TUBE1].FCS = Original FCS for tube 1
- AML[<LAB>][<CASE NUMBER>][TUBE2].FCS = Original FCS for tube 2
- AML[<LAB>][<CASE NUMBER>][TUBE3].FCS = Original FCS for tube 3
- AML[<LAB>][<CASE NUMBER>][TUBE4].FCS = Original FCS for tube 4
- AML[<LAB>][<CASE NUMBER>][TUBE5].FCS = Original FCS for tube 5
- AML[<LAB>][<CASE NUMBER>][TUBE6].FCS = Original FCS for tube 6
- AML[<LAB>][<CASE NUMBER>][TUBE7].FCS = Original FCS for tube 7

Within this study, each laboratory is referred to by a letter (“A” being lab one, “B” being lab two, etc), and each case is referred to by a sequential number (per center) padded to a length of three (“001”, “002”, “003”, etc.).

In total 803 cases were uploaded by 8 centers:

- Center A = 44 cases = A.001 up to A.044
- Center B = 79 cases = B.001 up to B.079
- Center C = 76 cases = C.001 up to C.376
- Center E = 31 cases = E.001 up to E.031
- Center F = 346 cases = F.001 up to F.346
- Center G = 211 cases = G.001 up to G.211
- Center H = 16 cases = H.001 up to H.016

Supplementary Data S3 – Initial Checks

Based on the various automated checks as discussed in detail below, in total 101 out of 803 cases were excluded (summarized in Table S3.1 at end of this Supplementary Data S3).

File Hashing

For each FCS and CYT file, from each case, the MD5 hash was calculated (a digital fingerprint). For example:

- MD5("tube 1 for A.001") = 8F98EAE8E804FB130F8096124BA34330
- MD5("tube 2 for A.001") = 18A4CC46A71D56F1EE37880C68F19E37
- MD5("tube 3 for A.001") = A0EA8FD72D8E57B4566A7FC6BCB27173
- MD5("tube 1 for A.002") = 800254B92A7C39E3A1415F4CFC954797
- MD5("tube 2 for A.001") = C415A87DFE667C1F52447BBE484073CB
- MD5("tube 3 for A.001") = 14D3DCC227A0E33FB56D3FF5E58A141B

Duplicate FCS files across cases

In case any duplicates were found, all corresponding/involved cases were excluded. For example:

- MD5("tube 1 for G.057") = EDCCD200853DFDC0C371404DDB744C07
- MD5("tube 1 for G.124") = EDCCD200853DFDC0C371404DDB744C07

Thus, G.057 and G.124 featured identical FCS files, and therefore both cases were excluded. In total, four of such pairs were identified (G.057/G.124, G.146/G.195, G.187/G.207 and G.196/G.208), resulting in eight exclusions.

Ambiguous FCS files within cases

Normally, for each case, one FCS file should be provided for each tube from the AML/MDS panel. However, for some cases, multiple FCS files were provided for a certain tube from the AML/MDS panel. For example:

- MD5(" first occurrence of tube 2 for G.039") = 5A249CA211A9468491D6B61AD7BA000B
- MD5("second occurrence of tube 2 for G.039") = 2934C9DABA2E57B0C8D97BAB4B89B93B

Thus, for G.039, two different FCS files were marked as being the second tube from the AML/MDS panel, resulting in significant ambiguity. In total, two cases with such ambiguities were excluded (G.029 and G.039).

Incomplete panel (only subset)

Only cases with tube 1 up to 6 from the AML/MDS panel were considered suitable (for now). For 35 cases, only the first 4 or 5 tubes were provided (e.g. the first 4 or 5 tubes were sufficient for routine diagnostic evaluation) and therefore these cases were excluded (C.015, C.021, C.027, C.028, C.029, C.030, C.037, C.038, C.043, C.044, C.045, C.046, C.048, C.050, C.051, E.001, E.002, E.003, E.004, E.007, E.008, E.009, E.010, E.011, E.016, G.033, G.056, G.145, G.184, G.194, G.197, G.199, G.203, G.209 and H.008).

Incomplete panel (tube missing)

For nine cases, no sequential set of AMD/MDS tubes was provided (i.e. one certain AML/MDS tube was missing). For example, for A.044, tube 4 was missing, while the other tubes (1, 2, 3, 5 and 6) were present. These cases were excluded (A.044, C.026, C.047, E.005, E.006, E.012, G.099, G.137 and G.185).

Analysis missing (no CYT file)

For 10 cases, no CYT file was provided along with the original FCS files. In other words, for these cases no analysis was available (i.e. no gating information for the lymphocytes), and therefore these cases were excluded (G.016, G.043, G.044, G.046, G.048, G.049, G.050, G.052, G.053 and G.149).

Analysis mismatch (FCS vs. CYT files)

For two cases, the CYT file was not based on the original FCS files, but on another set of FCS files. In other words, the analysis was based on another set of FCS files. These cases were excluded (A.005 and E.013).

Incomplete merge (incomplete CYT file)

For seven cases, the number of raw FCS files did not match the number of analyzed FCS files within the CYT file. For example, for A.012, the CYT file contained manual analysis results for tube 1 up to 5 from the AML/MDS panel, while raw FCS files were available for tube 1 up to 6 from the AML/MDS panel. These cases were excluded (A.012, G.062, G.071, G.073, G.074, G.189 and G.198).

Missing Markers

For 13 cases, one or more markers were lacking, presumably due to being out of stock at time of acquisition. In most cases, lack of marker was explicitly mentioned in the FCS file (e.g. “empty” instead of “CD16” as marker-name). In some cases, no marker-name was given, and for these the submitting center was asked to confirm whether the marker was. All cases with one or more markers missing were excluded (B.020/G.066/G.068/G.103/G.182/G.200 lacked CD10, G.010/G.183 lacked CD42aCD61, C.034 lacked CD64, G.045 lacked CD123, G.116 lacked CD203 and G.001/G.084 lacked multiple markers).

Missing compensation

For six cases, the original SPILL matrices (as produced by the cytometer) were missing ($SPILL keyword not included in the FCS files) and compensation was therefore based on manually defined SPILL matrices (presumably taken from other comparable cases). These cases were excluded (C.018, C.041, F.113, F.305, G.152 and G.153).

Too few lymphocytes

For ten cases, less than 450 lymphocytes events (arbitrarily chosen as cut-off) were present in at least one of the tubes. These cases were excluded (B.032=265, F.075=440, F.182=382, F.228=438, G.018, G.076=0, G.080=0, G.096=0, G.107=0, G.123=0). In addition, two cases (A.011, F.201) showed large variation in the percentage of lymphocytes between the six tubes (difference >30%) and were excluded as well (mean difference ± SD of all cases: 1.9% ± 2.9%).

Table S3.1. Overview of exclusions after initial checks

**Reason for exclusion**	**Number of cases**
Duplicate FCS files across cases	8
Ambiguous FCS files within cases	2
Incomplete panel (only subset)	35
Incomplete panel (tube missing)	9
Analysis missing (no CYT file)	10
Analysis mismatch (FCS vs. CYT files)	2
Incomplete merge (incomplete CYT file)	7
Missing Markers	13
Missing compensation	6
Too few lymphocytes (<450)	12
**Total exclusions after initial check**	**104**

Supplementary Data S4 – Sanitization

The EuroFlow protocols impose strict naming conventions, nevertheless, some deviations were identified, which were either accidental (due to typos such as “D16” instead of “CD16”, “CC35” instead of “CD35”, and “IREN2” instead of “IREM2”) or intentional (such as “CD300e” instead of “IREM2” and “7'1” instead of “NG2”).

Overview of the deviations:

- 16 alternatives for "CD42a+CD61", namely "CD42a+CD61", "CD42a + CD61", "CD42+61", "CD42a-CD61", "CD42A-CD61", "CD42+CD61", "CD42A+CD61", "CD42-CD61", "CD42-A+CD61", "CD42 A+CD61", "CD42aCD61", "CD42-61", "CD42A Y CD61", "CD42+CD 61", "CD42 Y CD61" and "CD61 + CD42a".
- 14 alternatives for "nuTdT", namely "TdT", "nTdT", "nTDT", "cTDT", "(nuTdT)", "CITOTDT", "CITO TDT", "CITIO TDT", "TDT CIT0", "CITO-TDT", "CITO TDT", "TDT CITO", "TDT-CITO" and "NuTdT".
- 5 alternatives for "IREM2", namely "CD300e", "IREM 2", "IREM", "IREM-2" and "IREN2".
  4 synonyms for "CD11b", namely "CD11B", "CD11 B", "CD11-B" and "CD11B".
- 3 synonyms for "CD203c", namely "CD203", "CD203C" and "CD 203",
- 3 synonyms for "HLADR", namely "Anti-HLA-DR", "HLA DR" and "HLA-DR".
- 3 synonyms for "NG2", namely "7-1", "7'1" and "7*1" (clone name).

Besides the minimally required channels (“FSC-A”, “FSC-H”, SSC-A”, four BB markers and four TS markers), some files also included additional channels (e.g. “TIME” or “SSC-H”) or the “-H” or “-W” equivalents for fluorescent channels (while the EuroFlow protocols only specify the “-A” equivalents for fluorescent channels). For each file, only the minimally required channels were exported to the new FCS files, and the names were corrected as well. Thus, for each tube from each case that passed the initial checks (n=699, details in **Supplementary Data S3**), a sanitized FCS file (i.e. uniformly formatted FCS file) was created, suitable for further processing.

Supplementary Data S5 – Establish BB Reference Data

For each tube, from each case, a matrix was obtained, with the BB markers as columns, and the lymphocytes as rows. Subsequently, the matrices were merged in a weighted manner, in terms of the absolute number of events, and in terms of BB stability. The resulting matrix was used as BB reference data. Details:

Merge Procedure - Schematic Overview

First, the sanitized FCS files (**Supplementary Data S4**) were read, and the TS markers (shown in red) were removed (e.g. CD16, CD13, CD11b and CD10 in case of tube one, and CD35, CD64, IREM2 and CD14 in case of tube two):

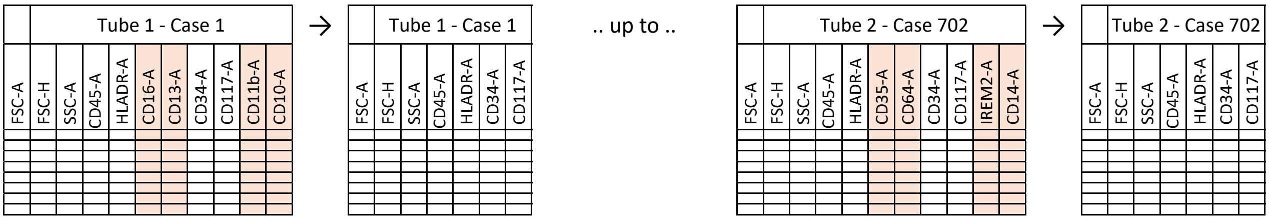

Second, the non-lymphocyte events (shown in red) were removed, thereby only leaving the lymphocyte events, thus the events identified as lymphocytes during manual analysis (**Supplementary Data S2**):

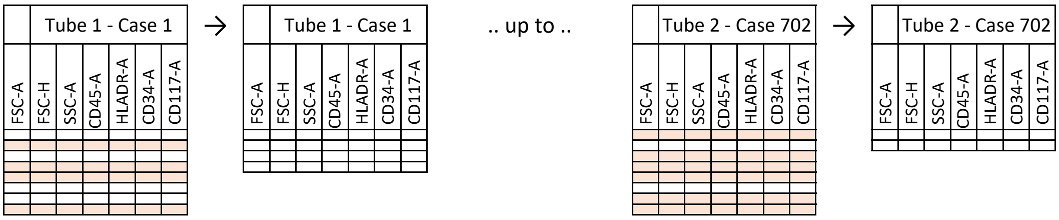

Third, the number of events (i.e. lymphocytes) was limited to a certain threshold (e.g. at most 2000 lymphocytes, see below), thus any event exceeding the threshold (shown in grey) was removed:

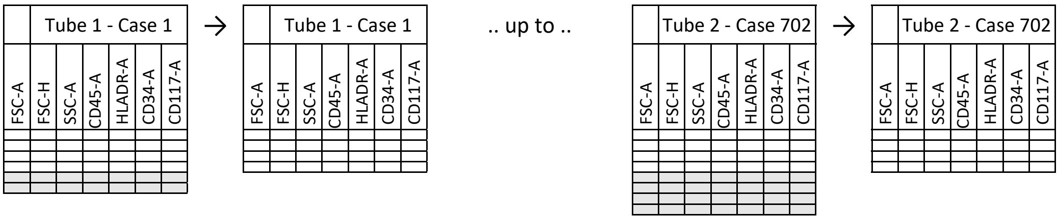

Finally, the resulting matrixes were merged in a row-by-row fashion:

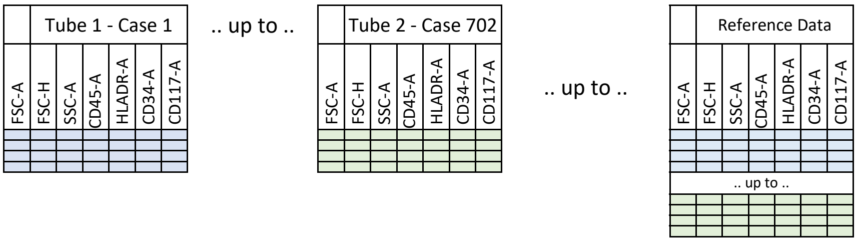

Optimizations

Two aspects of the merge procedure were optimized, namely threshold selection (i.e. keeping the highest number of lymphocytes per tube, while staying within practical memory limits, while avoiding overrepresentation of tubes with lots of lymphocytes) and case selection (i.e. cases with obvious BB abnormalities were not used to establish the BB reference data, and overrepresentation/underrepresentation of laboratories was taken in to account).

Optimizations - Event count

Given 699 cases (i.e. cases that passed initial checks) and 6 tubes per case (i.e. AML/MDS panel tube 1 up to 6), potentially up to 4.194 tubes could contribute to the BB reference data. Only 201 out of 4.194 tubes (4.7% of tubes, as shown by the horizontal red line) featured less than 2000 lymphocytes (as shown by the vertical red line). Obviously, these tubes always featured at least 450 lymphocytes, as cases with less than 450 lymphocytes were already excluded (**Supplementary Data S3**). By choosing a limit of 2000 events per tube, the vast majority of tubes contributed equally (in terms of the number of lymphocytes), and the maximum number of events (8.4240.00) was still within reasonable limits, in terms of memory usage. Empirical cumulative distribution function:

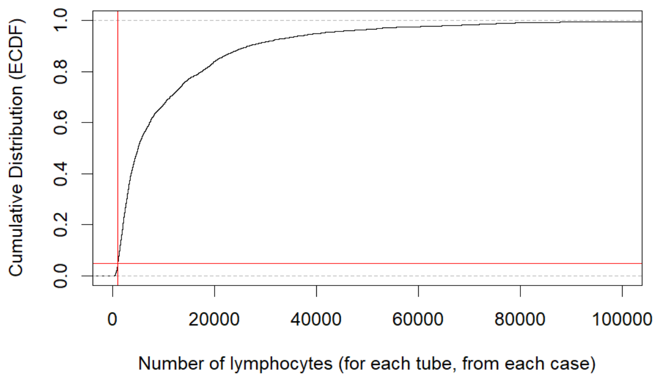

Optimizations - Case Selection

Only cases with lymphocytes that met the following criteria were considered for inclusion in the BB reference:

- FSC, SSC, CD34, CD117 and CD45, as measured by median, must be stable across each tube.
- HLADR must be bimodal in nature, with stable peak intensities across each tube.

Normal mature lymphocytes feature bimodal patterns for HLADR, and unimodal expression for FSC, SSC, CD34, CD117 and CD45. Mature lymphocytes are CD34 negative, CD117 negative, CD45 positive, and characterized by low FSC and SSC signals. However, the ratio of HLADR positive and HLADR negative lymphocytes is not fixed, as this depends on various factors, including the ratio of B/T cells, and the degree of activation. Therefore, stability of HLADR cannot be evaluated based on median values (as median is uninformative in case of bimodality), but must be evaluated based on peak intensities (i.e. intensity level where the negative and positive peak are located).

Optimizations - Case Selection - Peak Detection

First, for each tube, from each case, the nature of HLADR expression was determined, in terms of being unimodal or bimodal. For the vast majority of cases, this distinction was clear (each plot visualized one case; cases bimodal in nature at the top; cases unimodal in nature at the bottom; HLADR on the X axis; tube number at Y axis):

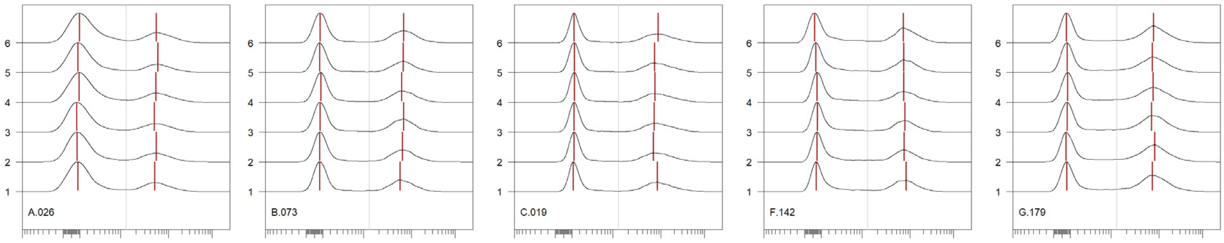

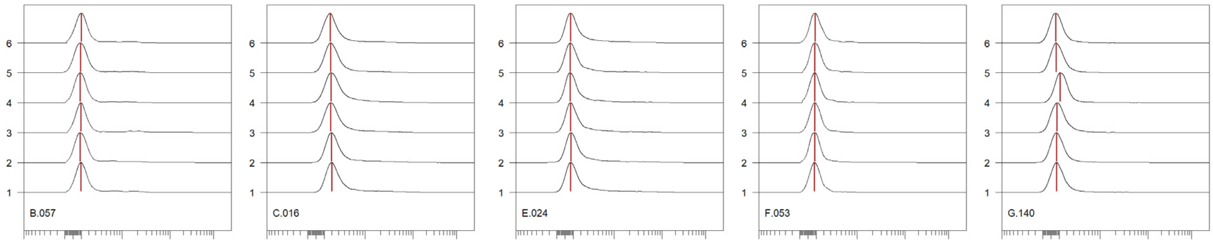

For some cases, the secondary peak was small, but nevertheless consistently found across tubes:

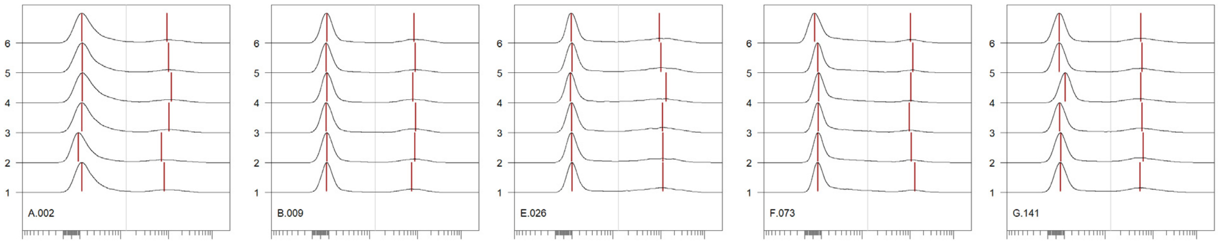

For some cases the distinction between unimodality and bimodality was less clear (minimal secondary peak):

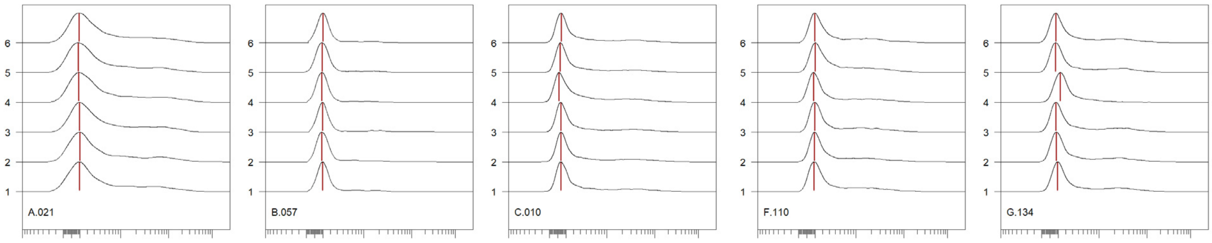

By using peak detection, irregularities in HLADR across tubes could be easily identified:

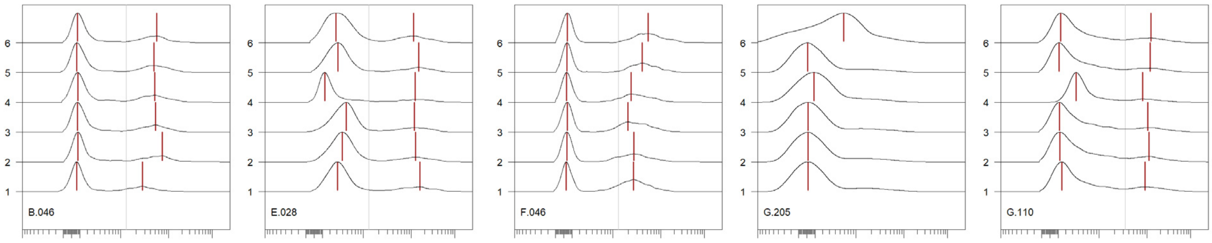

For each case, expression of each BB marker (on lymphocytes) is visualized for each tube, by using a plot as shown on the left (for each marker, tube 1 up to 6 is shown from left to right). For HLADR, the red lines visualize the peaks as detected before (shown on the right). For the other BB markers, the red lines visualize the median.

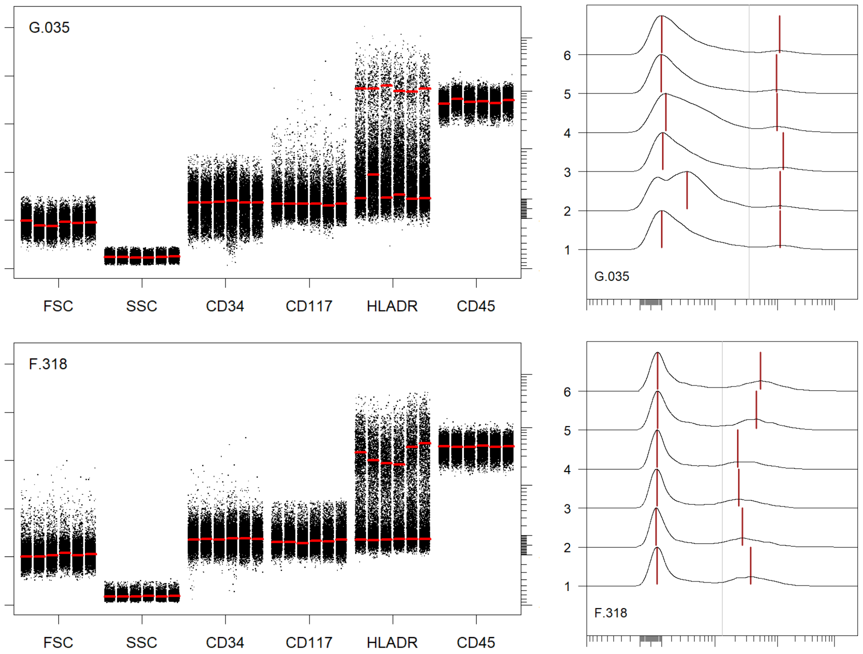

Optimizations - Peak intensity vs. Median intensity

Peak intensities were used in case of bi-modality (i.e. normal lymphocytes feature bi-modal HLADR expression, thus the negative and positive fraction were visualized based on their peak intensities), while median intensities were used in case of uni-modality (i.e. normal lymphocytes feature uni-modal FSC, SSC, CD34, CD117 and CD45 expression). Median has the benefit of being sensitive to skew (e.g. helps detecting any deviation from normality), which is very useful in case normality is expected (e.g. in case of FSC, SSC, CD34, CD117 and CD45). However, median has two major disadvantages when it comes to bi-modal distributions, namely being rather uninformative (in case the median is calculated for the entire bi-modal distribution) or being highly ambiguous (in case calculated for both distribution separately, which involves manual gating, in order to split the negative and positive fraction). In other words, using median intensities (in case of uni-modality) and peak intensities (in case of bi-modality) seems optimal in context of flow cytometric QC procedures (most informative and no ambiguity).

Optimizations - Case Selection - Overall Stability

For each marker, cases with significant differences in expression of the BB markers across tubes were identified (for each tube, the median expression was calculated (shown in red) and in case the lowest and highest value of the 6 tubes were more than 0.06 apart (on the normalized 0 to 1 scale) that marker was deemed to be too variable.

Out of 699 cases, 109 showed at least some degree of variability across tubes. Examples of cases with one or more BB markers variably expressed in one or more tubes: are shown below:

- Cases with variable primary HLADR peaks (left) or variable secondary HLADR peaks (right) across tubes:

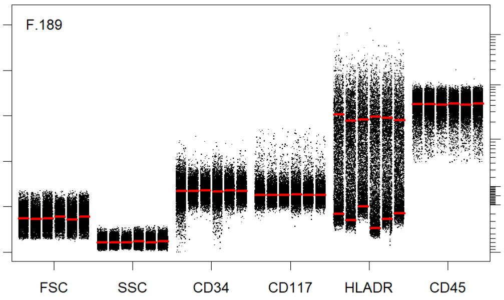

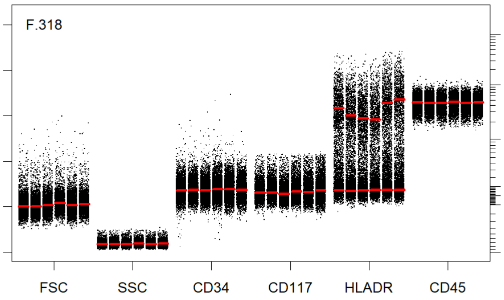

- Cases with variable median FSC values (left) or variable median CD45 values (right) across tubes:

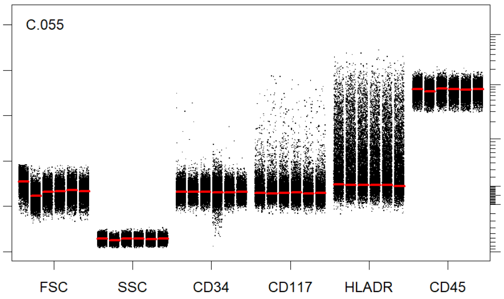

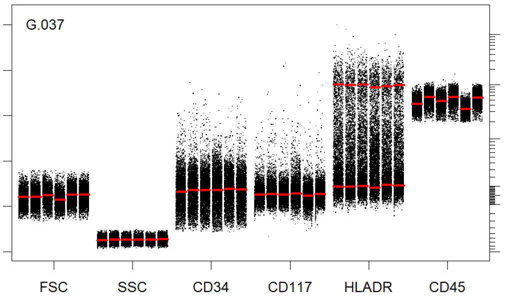

- Cases with variable median CD34 values (left) or variable median CD117 values (right) across tubes:

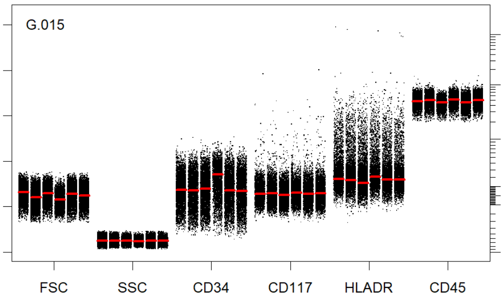

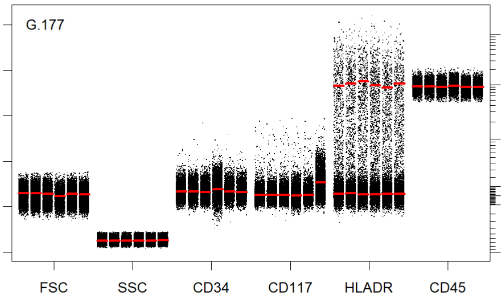

- Cases where multiple markers from one tube were affected (e.g. FSC, CD34 and HLADR from tube 4):

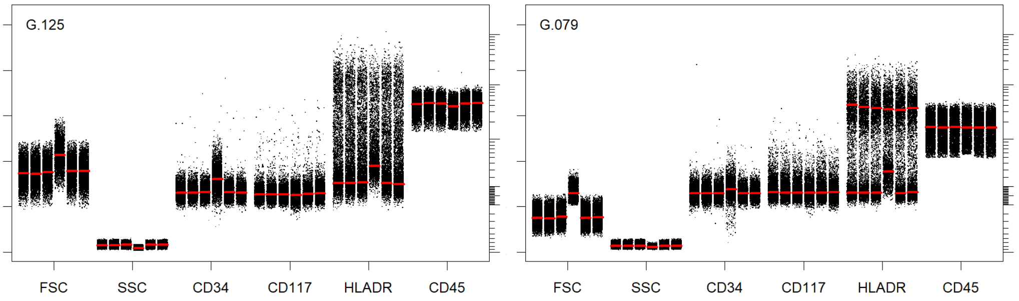

- Cases where multiple markers from multiple tubes were affected:

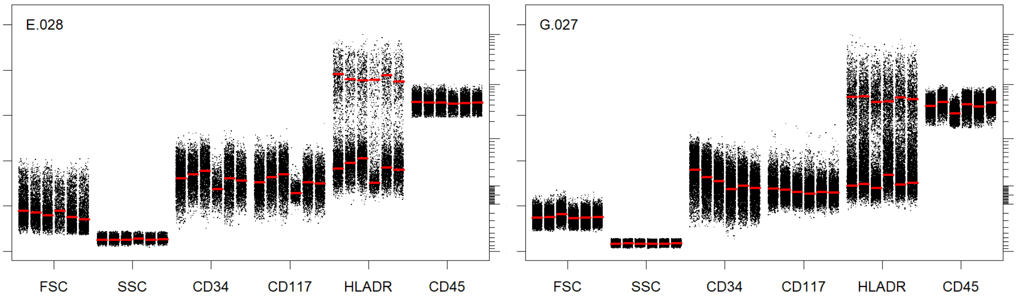

In total 209 cases featured highly stable peaks/medians and <u>unimodal</u> HLADR expression:

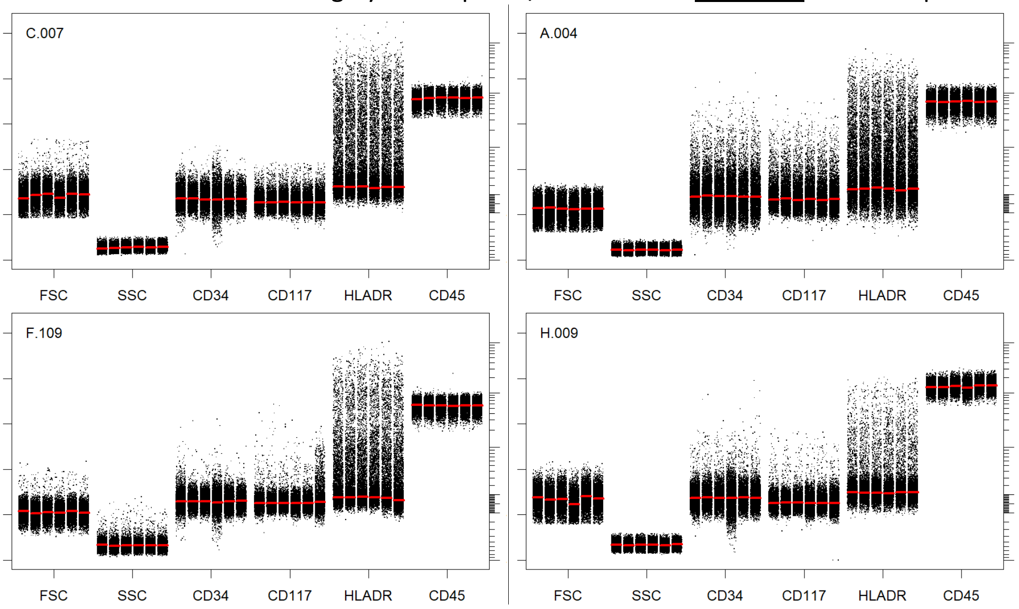

In total 384 cases featured highly stable peaks/medians and bimodal HLADR expression:

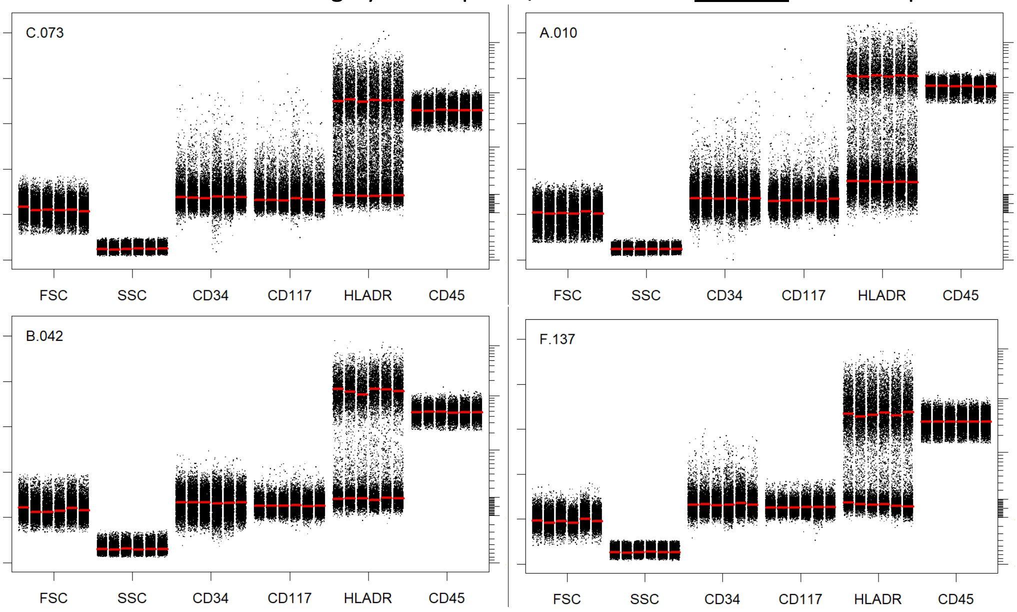

Optimizations - Case Selection - Conclusion

In conclusion, the BB reference data was based on 384 cases with stable BB marker expression (across tubes) and bimodal HLADR expression. From each tube, from the aforementioned 384 cases, up to 2000 lymphocytes were taken and merged together (resulting in a matrix with BB expression for 4.394.241 lymphocytes).

Supplementary Data S6 – Establish BB Reference Region

Previously, the BB reference data was established by aggregating lymphocytes across tubes and cases (**Supplementary Data S5**).

Principle - Establishing BB Reference Region

Here, the BB reference region will be established, based on the BB reference data. First, the fluorescent channels were transformed (using a logicle transformation with default settings: w = 0.8, t = 512^2, m = 4.5, a = 0). Second, principal component analysis (PCA) was performed, to obtain the first and second principal component (PC1 and PC2 respectively). Third, the densest region within the PC1 versus PC2 space was identified via hexbinning (i.e. the smallest region which constituted to 95 percent of the data points). In the end, essentially, the eigenvectors as obtained during PCA (thus the information needed to calculate PC1 and PC2 from the raw data) together with the polygon as obtained during hexbinning (thus the coordinates of the yellow line as shown below) were considered to be the BB reference region. Schematic overview:

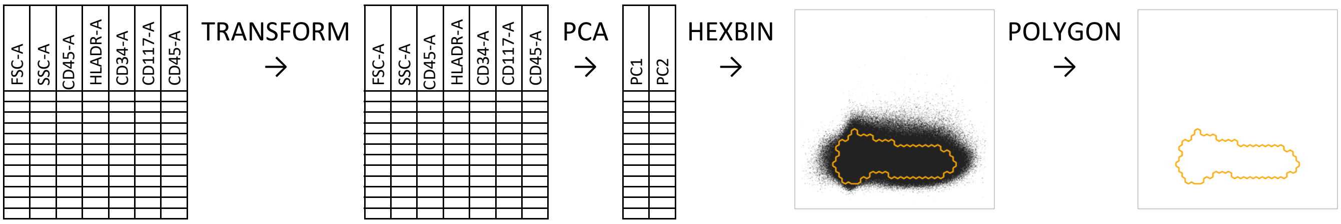

Principle – Using BB Reference Region

Any case can be checked against the established BB reference region, by using the eigenvectors and polygon. First, the data from the case in question must be transformed (the transformation must be identical to the transformation in the aforementioned procedure). Second, based on the eigenvectors, the PC1* and PC2* must be calculated (marked by asterisk, because these are not the true PC1 and PC2 of the case in question, but the PC1 and PC2 as if the case in question would've been plotted on top of PCA in the aforementioned procedure). Third, the polygon is added and the percentage of data points within the polygon is calculated. Overview:

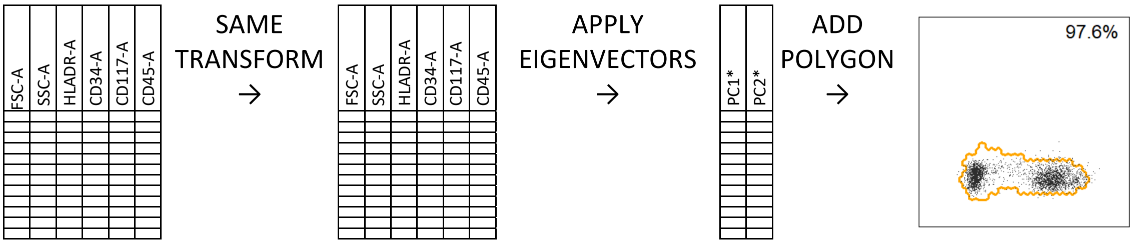

Result

Given the previously established BB reference data (**Supplementary Data S5**) and a density threshold of 95 percent, the following BB reference region was established, based on the aforementioned method (left panel shows the PCA, center panel shows the hex-binning result, right panel shows the final polygon):

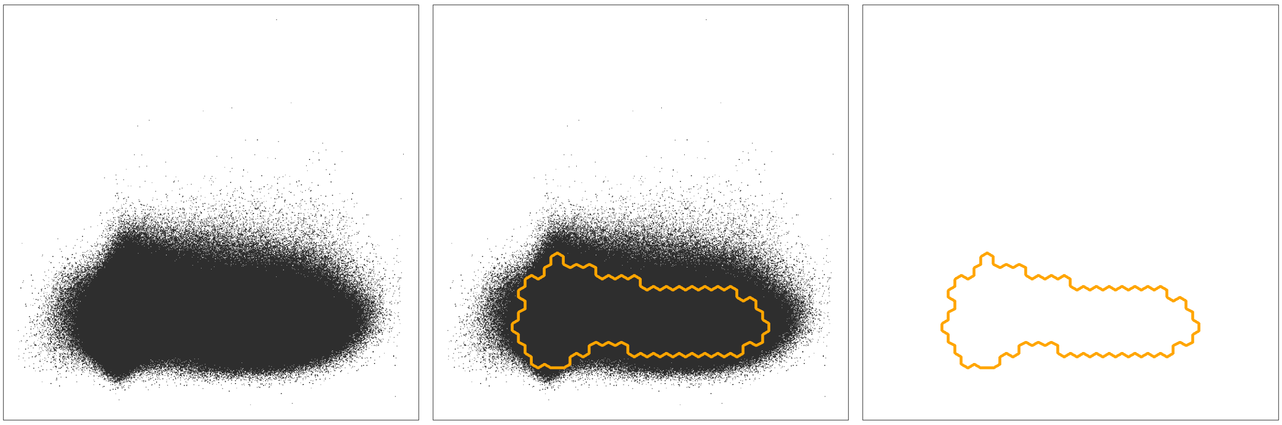

For validation purposes, the transformed BB reference data was read into Infinicyt, and a PCA analysis was performed (visualized in the “APS” window). The results are identical:

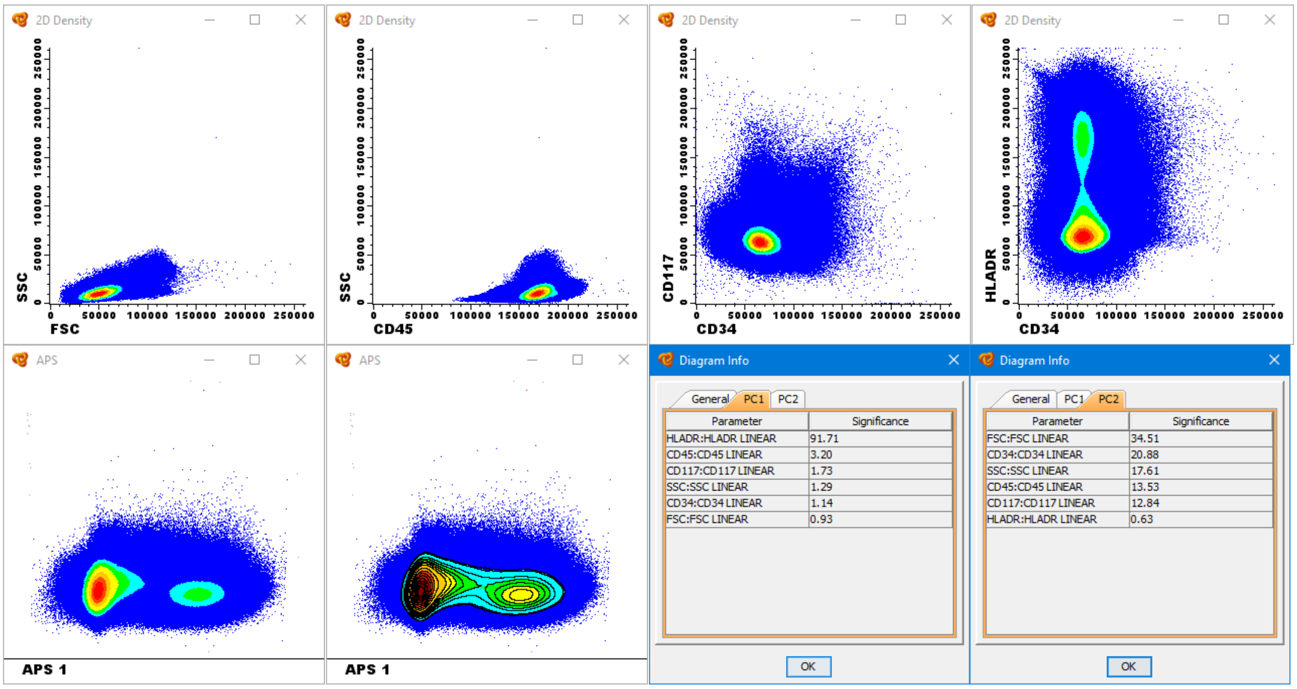

Result – Loadings Of Variables

PCA results can be visualized via bi-plots, which essentially visualize the loadings of the variables (in this case the "contribution of BB markers to PC1 and PC2") on top of the scores (in this case "PC1 vs. PC2 for lymphocytes"). The bi-plot based on the BB reference data (left) and the corresponding normalized loadings (right):

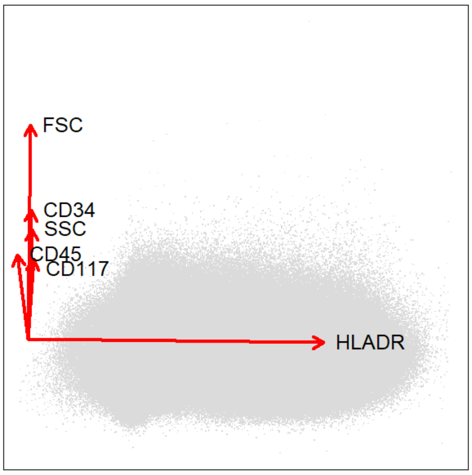

	PC1	PC2
**FSC**	0.9 %	34.5 %
**SSC**	1.3 %	17.7 %
**CD34**	1.1 %	20.8 %
**CD117**	1.8 %	12.8 %
**HLADR**	91.6 %	0.6 %
**CD45**	3.3 %	13.6 %

Given the bi-plot above, obviously "PC1 reflects HLADR" while "PC2 reflects a mixture of the other markers", which simplifies the interpretation of BB reference plots. For example, cases with average BB patterns fall within the reference region (top left panel), cases with relative high expression for any of "the PC2 contributing markers" (i.e. CD117, CD45, SSC, CD34 and FSC) shift upwards (top center panel), cases with relative low expression for any of these markers shift downwards (top right panel) and cases with abnormal HLADR expression patterns can be identified as well (bottom three panels):

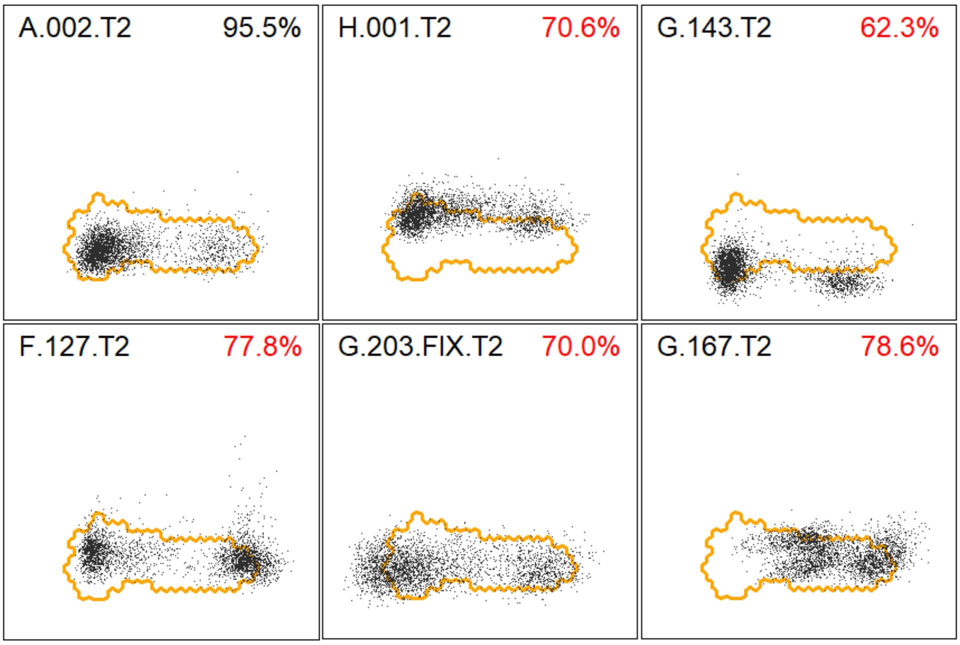

Optimization

Obviously, the threshold of choice heavily influences the polygon (in terms of area and shape). As compared to the threshold of choice (i.e. 95 percent), lower thresholds result in smaller polygons (e.g. 91 and 93 percent), while higher thresholds result in larger polygons (e.g. 97 and 99 percent):

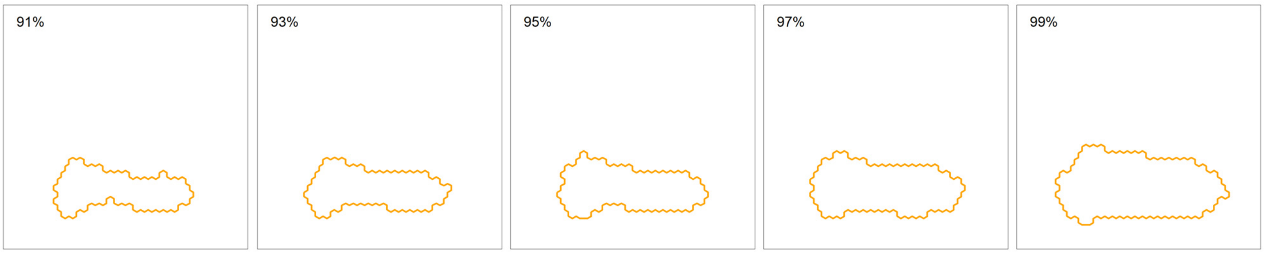

Interestingly, the polygon area plateaus for density thresholds within the 94 up to 96 percent range (small plateau as shown in grey) and increases rapidly for thresholds above 96 percent (right side of grey area):

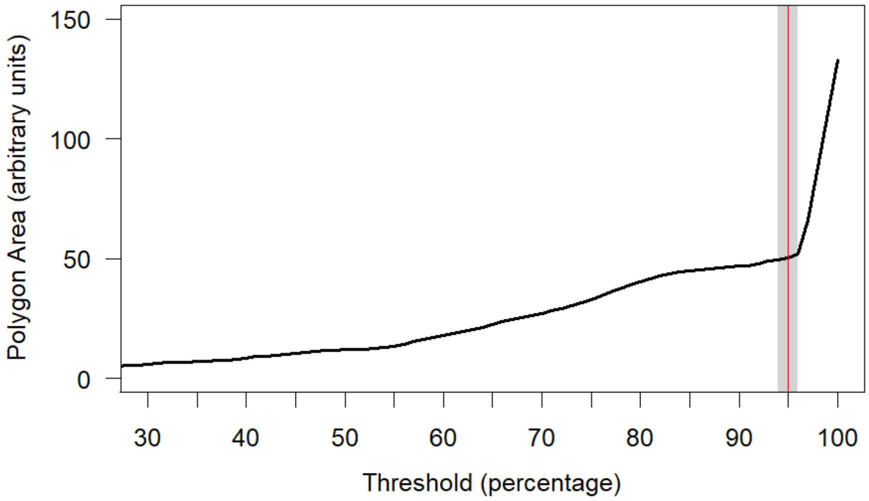

Thus, thresholds within the 94 up to 96 range (shown in grey) result in near identical polygons (i.e. small threshold changes barely affect the polygon area) while the opposite holds true for thresholds above this range (i.e. small threshold changes heavily affect the polygon). Altogether, a threshold of 95 percent (show in red) was chosen, being nearly identical to any threshold within the 94 up to 96 percent range (in terms of polygon size and shape), and being located right before the obvious change in trend.

Supplementary Data S7 – Check cases against BB Reference Region

For each case, each tube was checked against the established BB reference region (**Supplementary Data S4**). Thus, for each tube, the percentage of lymphocytes inside the BB reference region was calculated, resulting in the following matrix (16 out of 702 cases shown, where "BB.AML.1" to "BB.AML.6" gives the percentage for tube 1 to 6 respectively):

CASE	BB.AML.1	BB.AML.2	BB.AML.3	BB.AML.4	BB.AML.5	BB.AML.6	MEDIAN
F.196*	99.7	99.6	99.6	99.8	99.8	99.7	99.7
B.015	99.2	99.5	99.5	99.0	99.4	99.2	99.3
C.052*	98.3	98.4	97.9	98.6	97.7	97.8	98.1
G.160	98.2	97.9	98.5	98.3	98.2	98.1	98.2
A.017*	95.3	95.2	95.1	95.7	92.8	94.5	95.1
F.281	94.3	96.1	95.1	95.0	94.6	96.2	95.0
B.063*	90.8	90.2	88.7	87.4	90.6	91.2	90.4
G.165	89.5	90.7	91.0	88.5	89.3	91.9	90.1
F.213*	84.1	86.4	86.9	84.7	84.1	85.4	85.0
A.036	84.0	86.0	85.9	81.5	85.8	85.0	85.4
A.037*	76.9	74.9	76.6	76.1	72.2	73.4	75.5
G.190	70.7	75.5	76.5	75.1	77.8	73.7	75.3
H.010*	79.9	69.3	72.3	69.9	70.3	65.4	70.1
H.001	72.8	70.6	70.0	71.3	78.8	56.6	70.9
F.189*	56.8	57.2	71.4	54.1	56.8	66.0	57.0
G.083	57.9	55.8	54.8	58.2	56.1	61.1	57.0

In this study, tubes with less than 80 percent of their lymphocytes within the BB reference region were considered "out-of-reference" (indicated by red color). Cases with one or more tubes "out of reference" were marked, resulting in 125 out of 699 cases being marked (details in **Supplementary Data S8**). For various cases (as indicated by asterisk), the calculation is visualized on the next page (tube 1 up to 6 indicated by T1 up to T6 respectively). On the page thereafter, the 80 percent cutoff is explained in detail.

F.196 (median 99.7 percent)

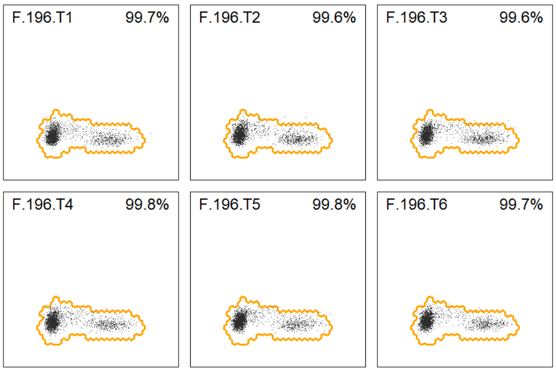

C.052 (median 98.1 percent)

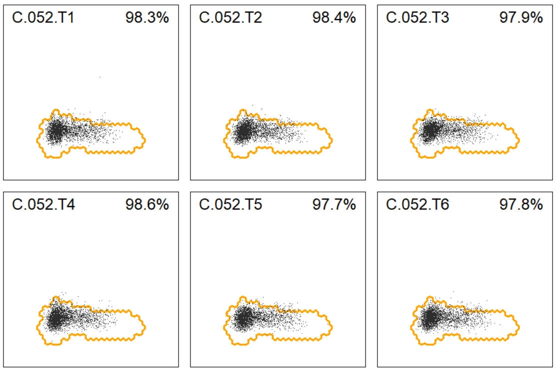

A.017 (median 95.1 percent)

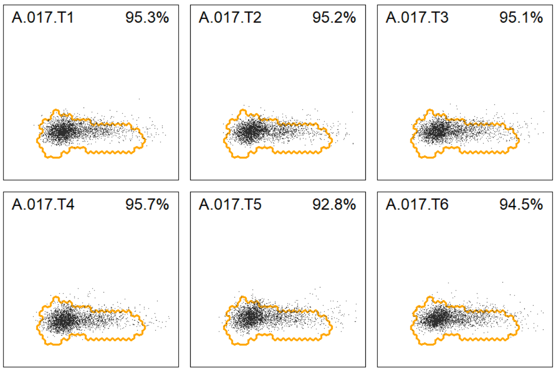

B.063 (median 90.4 percent)

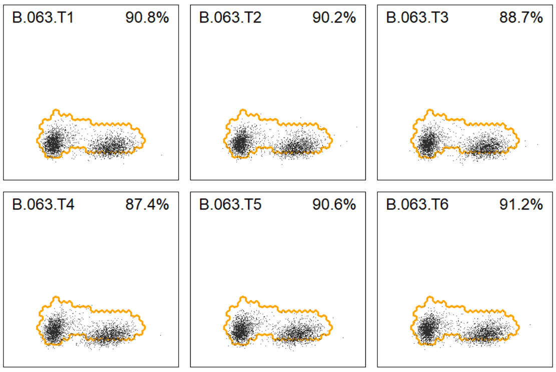

F.213 (median 85.0 percent)

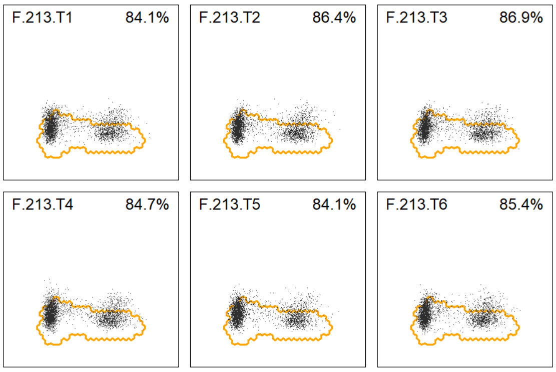

A.037 (median 75.5 percent)

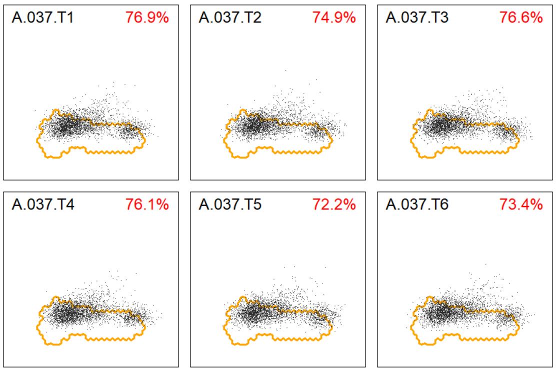

H.010 (median 70.1 percent)

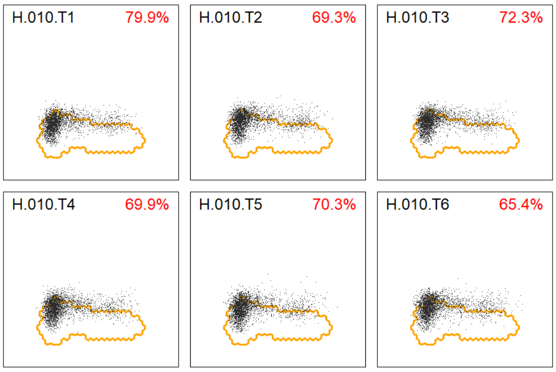

F.189 (median 57.0 percent)

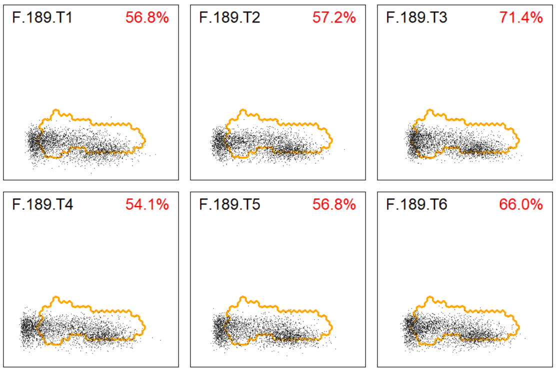

Optimization

The number of cases selected for exclusion is directly influenced by the cutoff of choice. For example, the following case was excluded due to tube 4 being out of reference (given the 80 percent cutoff), but would not have been excluded in case a slightly lower cutoff was chosen (i.e. anything below 78.7 percent)

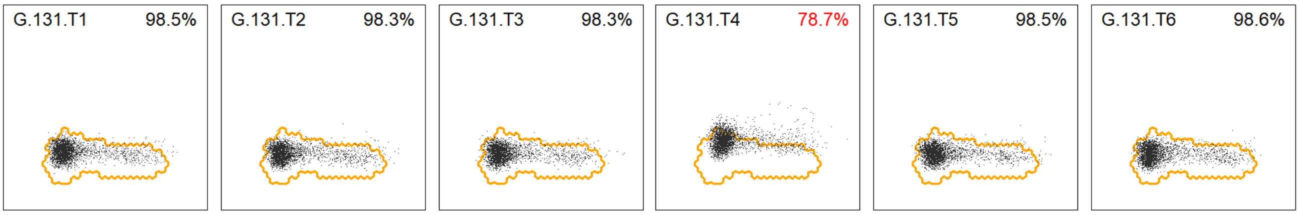

Interestingly, the relation between cutoff and exclusions reveals a small plateau in the 79 up to 80 range, and grows rapidly thereafter:

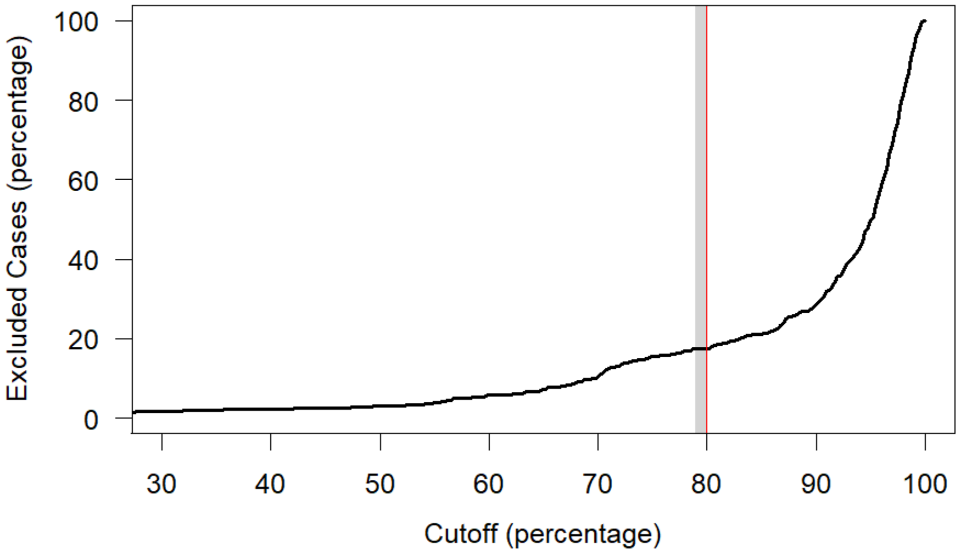

Thus, any cutoff within the 79 up to 80 range results in the same number of cases being marked, while any cutoff above this range should be handled with care, as arbitrary small changes directly influence the number of cases being flagged (e.g. by increasing the cutoff from 80 to 90 results in many additional cases being flagged). Therefore, choosing a cutoff of 80 percent seems reasonable (give the “natural break” in the cohort).

Supplementary Data S8 – Review of marked cases (BB)

By checking against the BB reference region (i.e. "the PCA based approach"), in total 125 cases were marked (details in **Supplementary Data S7**). Whether these cases deviated in any meaningful way from the cohort average, was evaluated by comparing "the PCA based approach" against the more classical "median/peak based approach".

Visualization

Previously, for each case, the expression of each BB marker (on lymphocytes) was visualized, for each tube, in side-by-side fashion (details in **Supplementary Data S5**). Exemplary figure for case C.073 with the median/peak intensities in red:

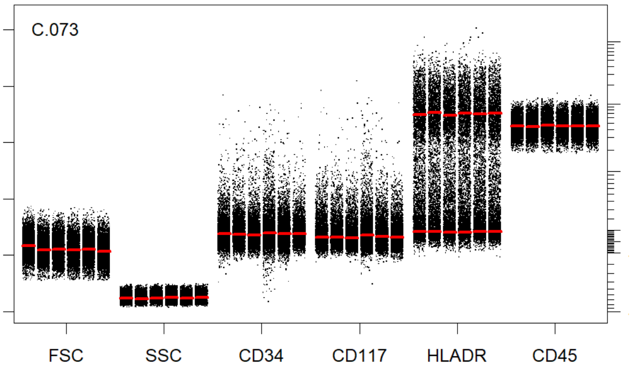

These median/peak intensities (as shown in red in the next figure) were aggregated (for each case, each tube, each parameter) and visualized (top left panel, where each grey bar represents one case). Based on these values, boxplots were created (top right panel). Subsequently, for each case (e.g. C.073), the median/peak intensities were plotted on top of these figures with aggregated data, allowing an intuitive visual review (bottom panels):

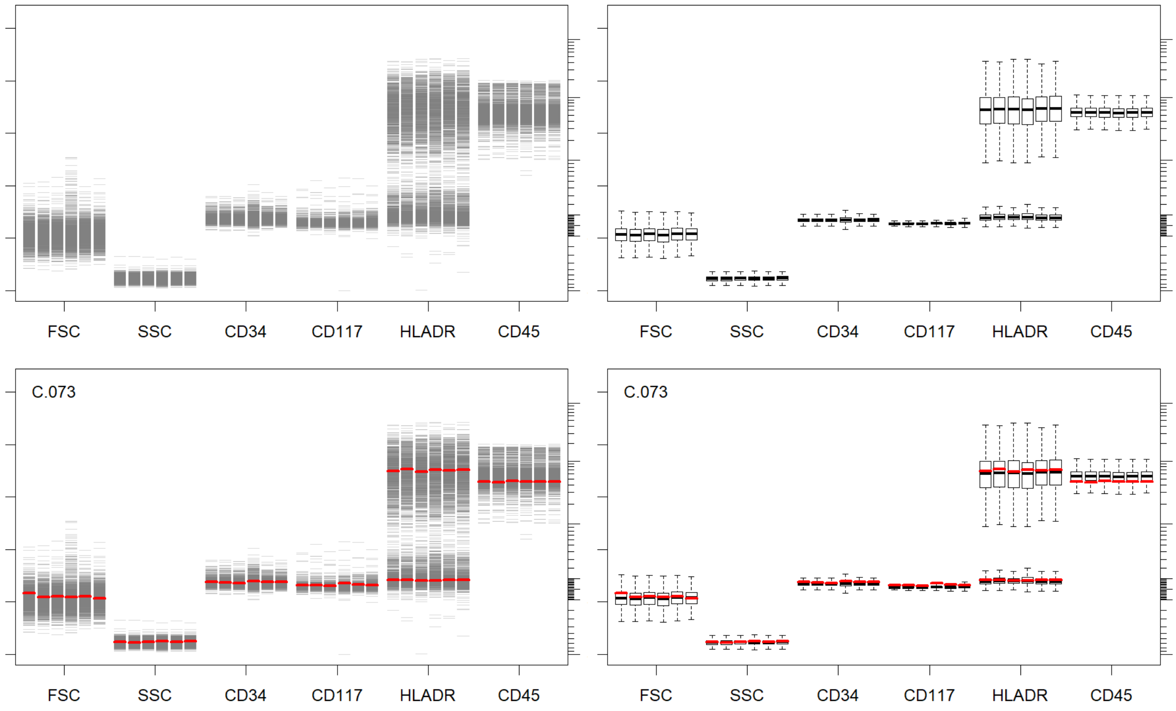

Review

For each case, the plot with median/peak intensities (on top of the aggregated data) was combined with the previously generated BB reference plot (details in **Supplementary Data S7**). This way, the “the PCA based approach” can be easily compared against the “the median/peak-based approach”.

Cases within BB reference region

Six exemplary cases, with the vast majority of their lymphocytes (>80%) within the BB reference region:

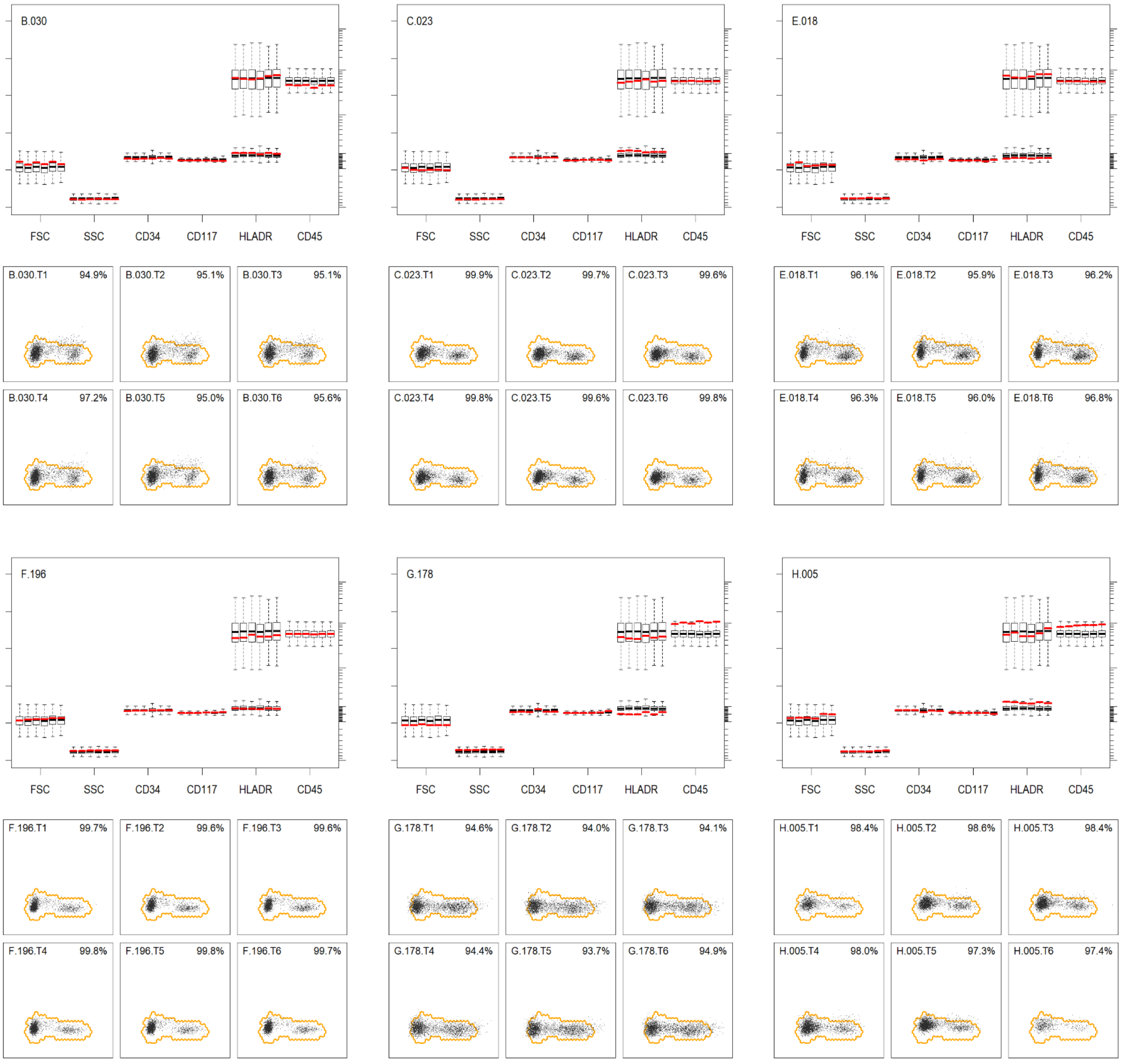

Cases outside BB reference region

Based on the previously established exclusion criteria (one or more tubes with less than 80 percent of lymphocytes within reference, details in **Supplementary Data S7**), in total 125 out of 699 cases were marked. These 125 cases could be categorized in various groups:

- One marker within one tube being out-of-reference (n=13);
- One marker within every tube being out-of-reference (n=19);
- Multiple marker within every tube being out-of-reference (n=27);
- Multiple markers within one tube being out of reference (e.g. FSC, CD34 and HLADR in the fourth tube) (n=33);
- Any other subset of markers and tubes being out-of-reference (n=33).

Examples of each category are shown below:

- One marker within one tube being out-of-reference (n=13):

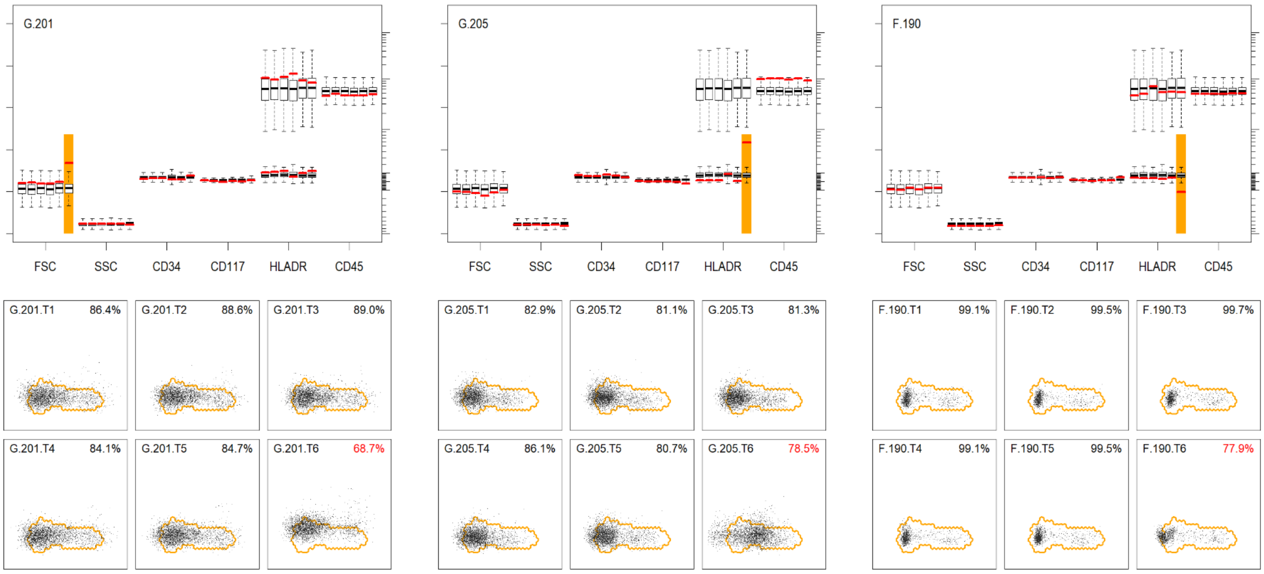

- One marker within every tube being out-of-reference (n=19):

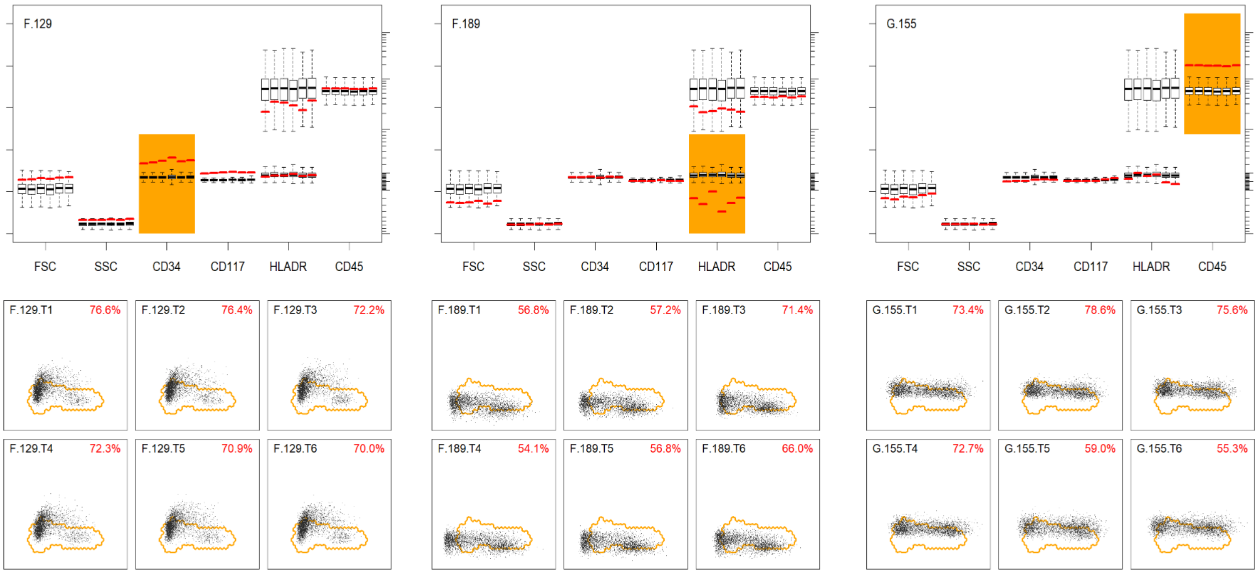

- Multiple marker within every tube being out-of-reference (n=27):

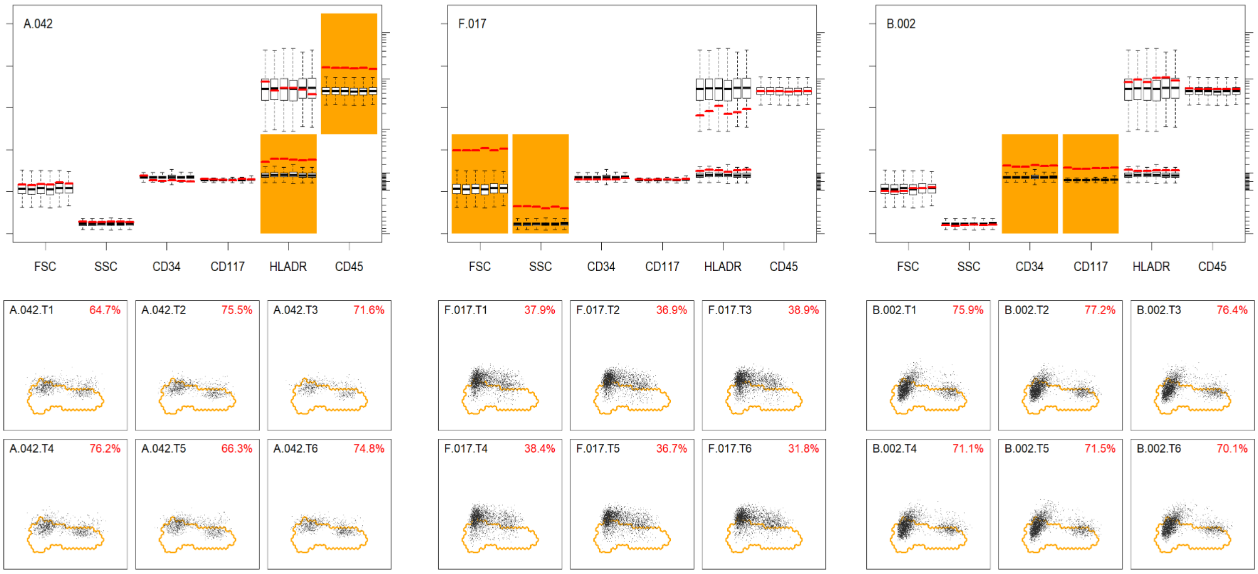

- Multiple markers within one tube being out of reference (e.g. FSC, CD34 and HLADR in the fourth tube) (n=33):

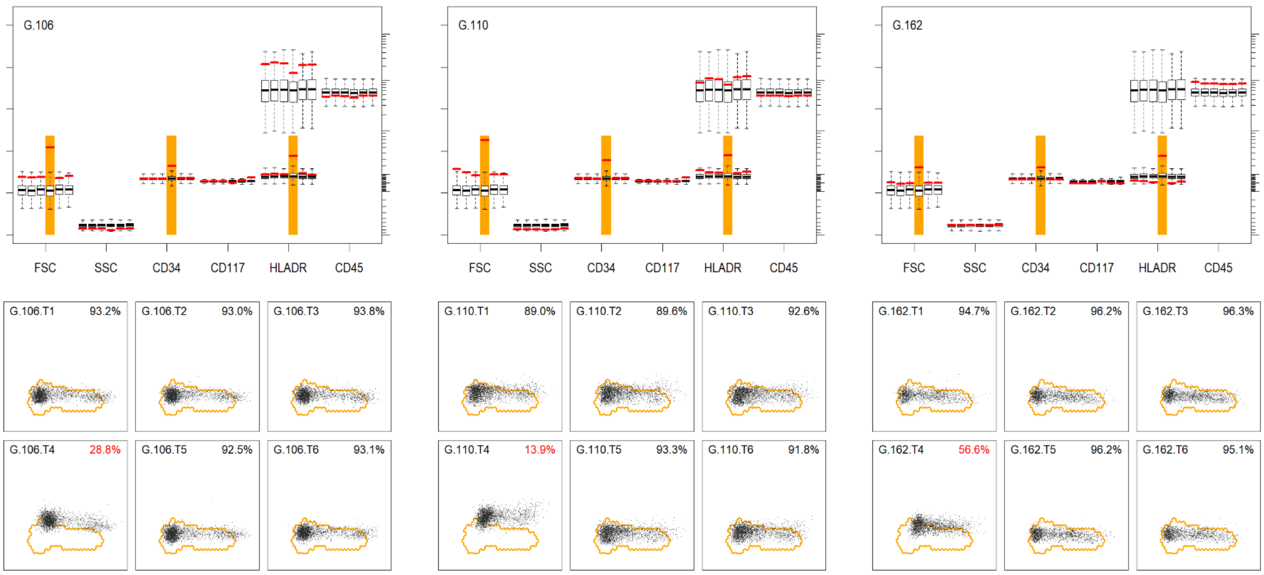

- Any other subset of markers and tubes being out-of-reference (n=33):

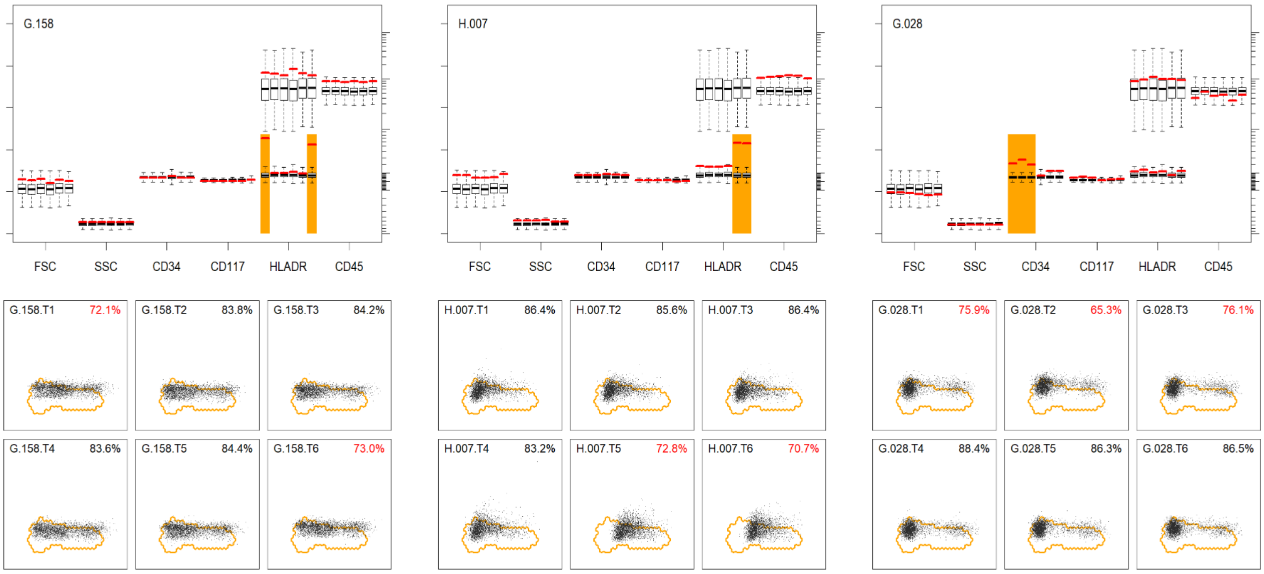

Consistency

Notably, “the PCA based approach” and the “the median/peak based approach” showed very strong correlations, in terms of being able to detect the same abnormalities (i.e. certain markers/tubes being out-of-reference).

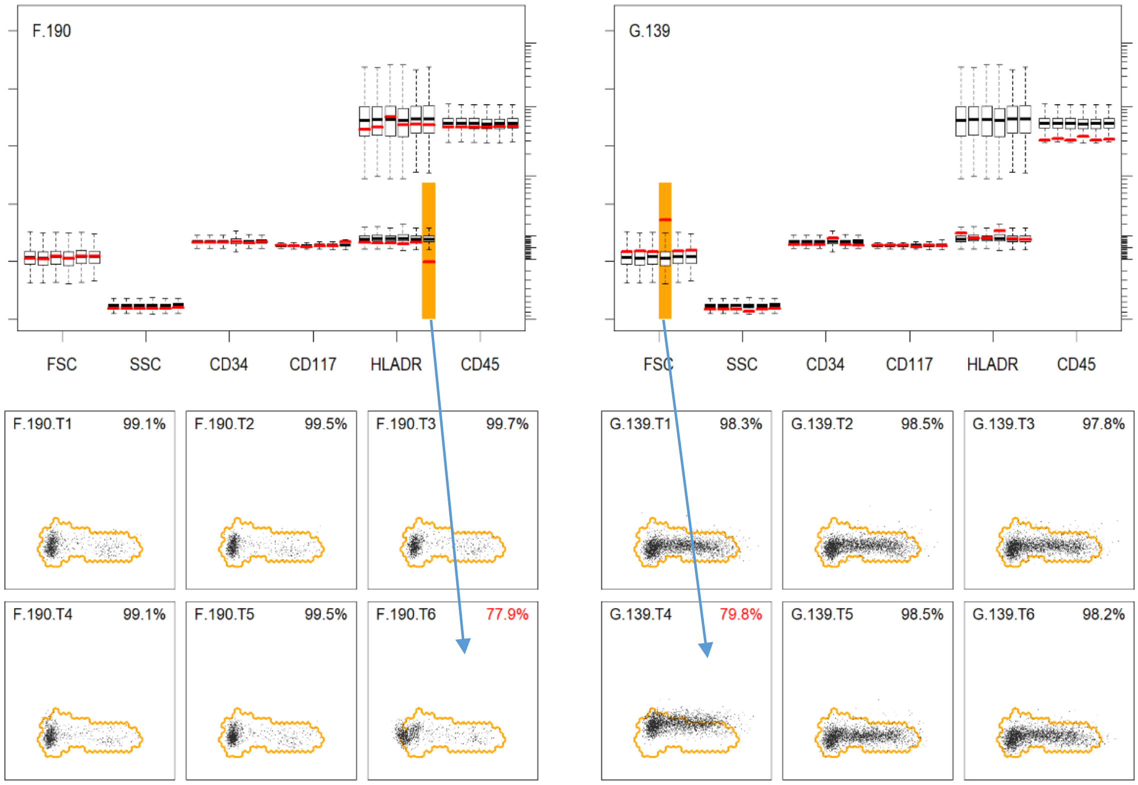

Explanation

Notably, by reviewing the PCA plots, one can easily identify negative deviations (e.g. HLADR on the left side) or positive deviations (e.g. FSC on the right side). In other words, the PCA plots preserve directionality.

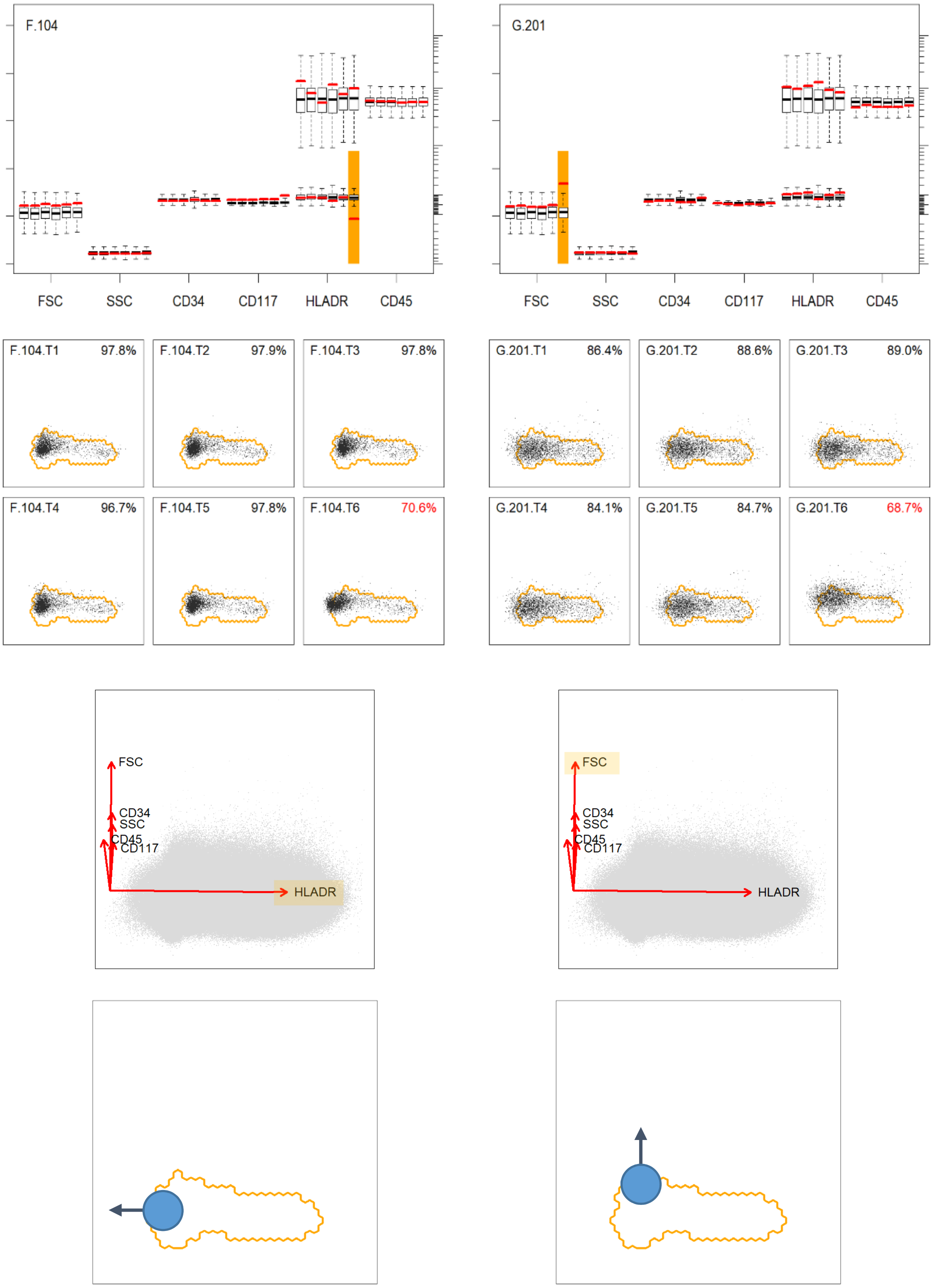

Supplementary Data S9 – Establish TS Reference Data

For each tube from the AML/MDS panel, a reference was created, based on the TS markers for that specific tube. Only cases with stable BB markers (i.e. cases that passed the BB reference region check, details in **Supplementary Data S7**) were used to establish this reference. For example, for the first tube from the AML/MDS panel, the reference was created by obtaining a matrix from each case (with the TS makers of interest as columns and the lymphocytes as rows) and merging these matrixes in a weighted manner (in terms of the absolute number of events). This process was repeated for each tube from the AML/MDS panel (i.e. with the corresponding TS markers).

Merge Procedure - Schematic Overview - Based On AML/MDS Tube 1

First, the sanitized FCS files with AML tube 1 data (**Supplementary Data S4**) were read, and the BB markers (shown in red) were removed (FSC-A, FSC-H, SSC-A, HLADR-A, CD34-A and CD117-A):

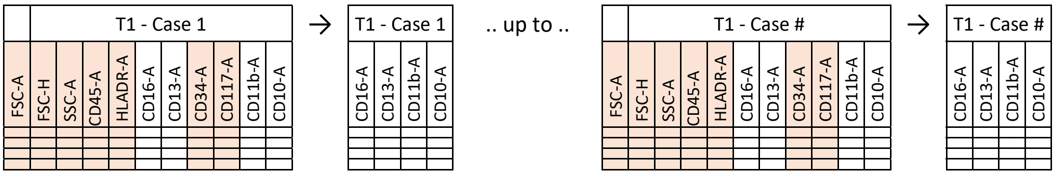

Second, the non-lymphocyte events (shown in red) were removed, thereby only leaving the lymphocyte events, thus the events identified as lymphocytes during manual analysis (**Supplementary Data S2**):

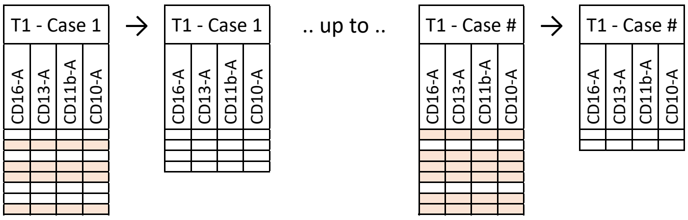

Third, the number of events (i.e. lymphocytes) was limited to a certain threshold (e.g. at most 2000 lymphocytes), thus any event exceeding the threshold (shown in grey) was removed:

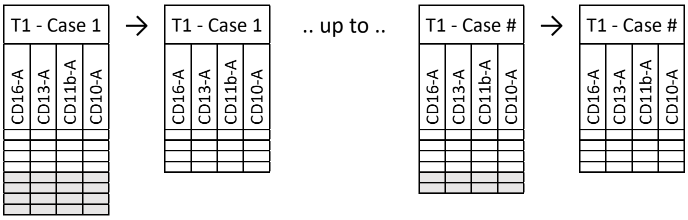

Finally, the resulting matrixes were merged in a row-by-row fashion:

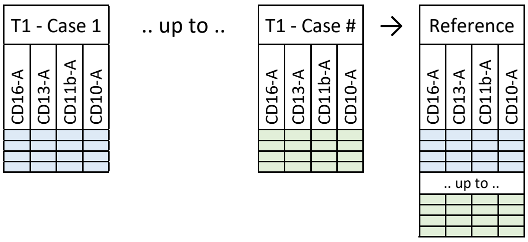

Optimizations

Similar to the BB procedure (**Supplementary Data S5**), the threshold was optimized (i.e. keeping the highest number of lymphocytes per tube, while staying within practical memory limits, while avoiding overrepresentation of tubes with lots of lymphocytes). This also resulted in a threshold of 2000 events.

Supplementary Data S10 – Establish TS Reference Region

Previously, the TS reference data was established by aggregating lymphocytes across tubes and cases (**Supplementary Data S9**). Here, the TS reference regions will be established based on the TS reference data. This procedure was comparable to the BB reference region procedure (**Supplementary Data S6**), with the only difference being the markers (i.e. BB vs. TS markers). It should be emphasized that, while one BB reference region could be used for every AML/MDS tube (since every tube contains the same BB markers, by definition), one TS reference region was needed for every AML/MDS tube (since the TS markers vary across tubes, by definition).

Principle - Establishing and using the TS reference region

Since this procedure is comparable to the BB reference region procedure, the same technical description applies (details in **Supplementary Data S6**). For clarity, the same schematic overview is provided, but now for the TS reference region as established for the second tube from the AML/MDS panel:

From TS reference data (left) to TS reference region (right):

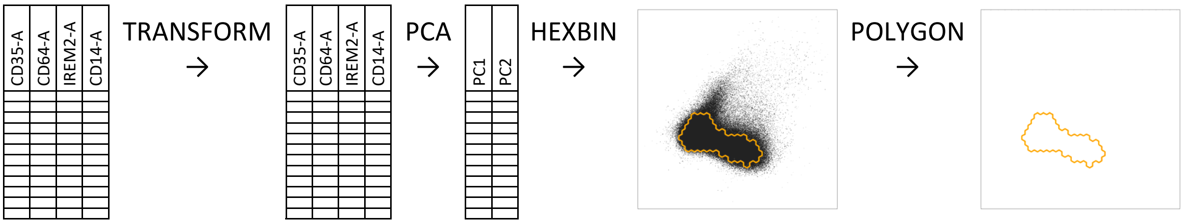

Checking an individual case (left) against the established TS reference region (right):

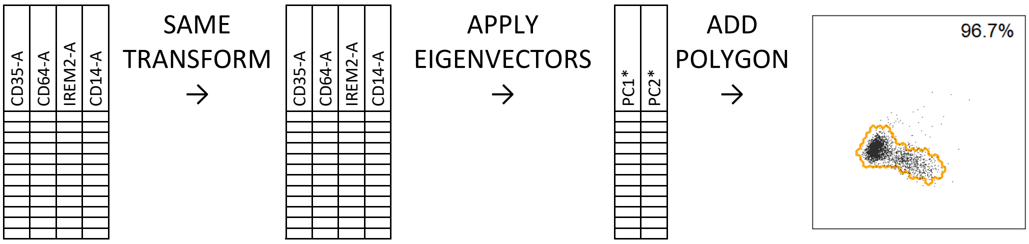

Results

Given the previously established TS reference data (**Supplementary Data S9**), the following TS reference regions were established, by relying on the same 95 percent density threshold as used to establish the BB reference region (**Supplementary Data S9**):

Tube 1

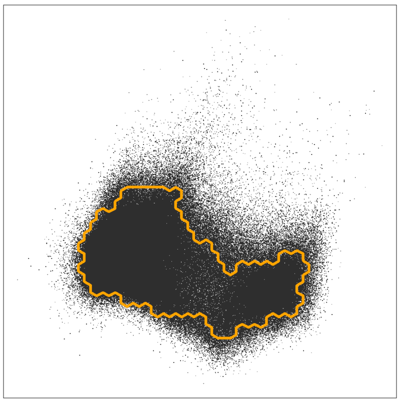

Tube 2

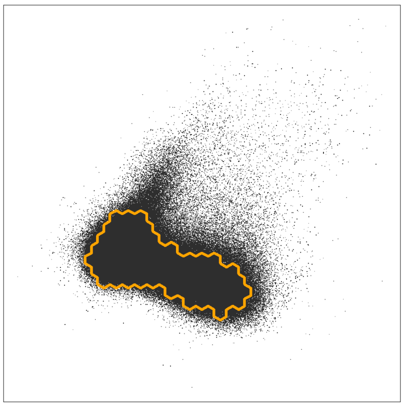

Tube 3

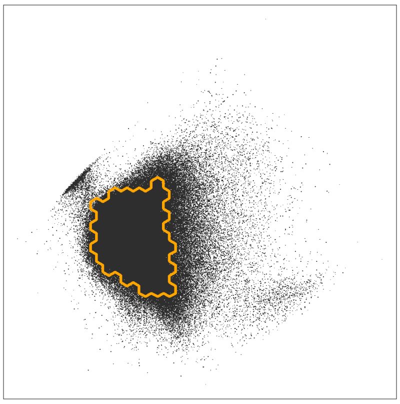

Tube 4

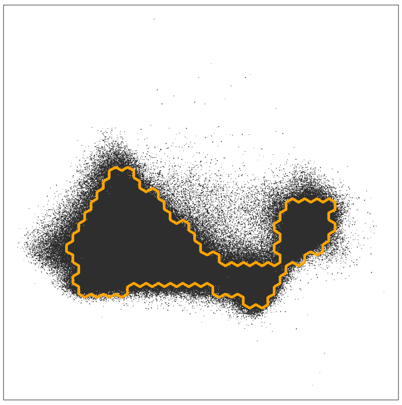

Tube 5

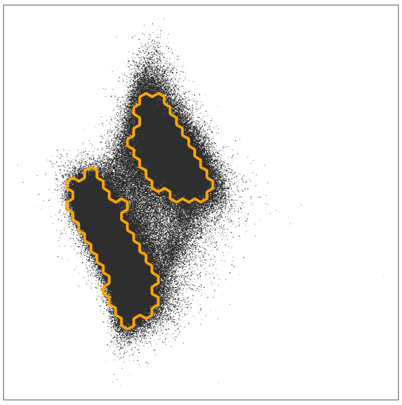

Tube 6

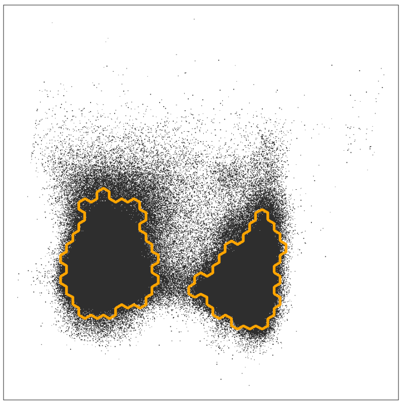

Result – Loadings of variables

Like previously, the contributions of individual markers were visualized in biplots (details in **Supplementary Data S6**):

Tube 1

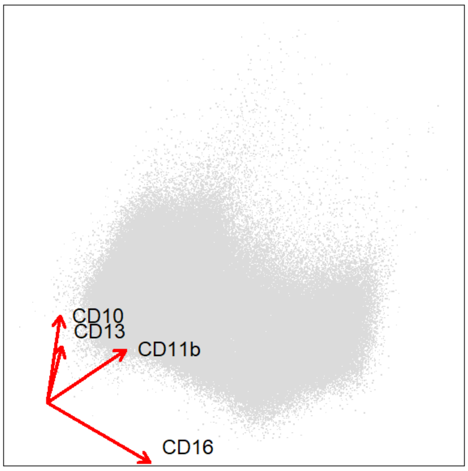

Tube 2

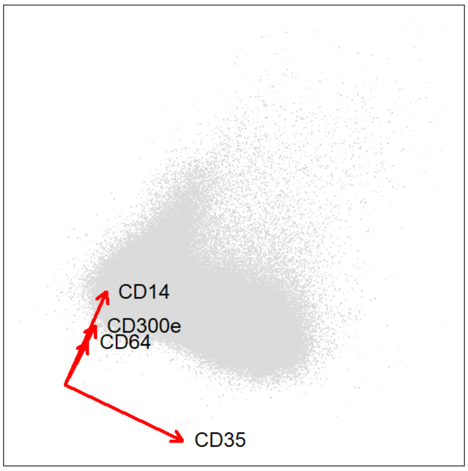

Tube 3

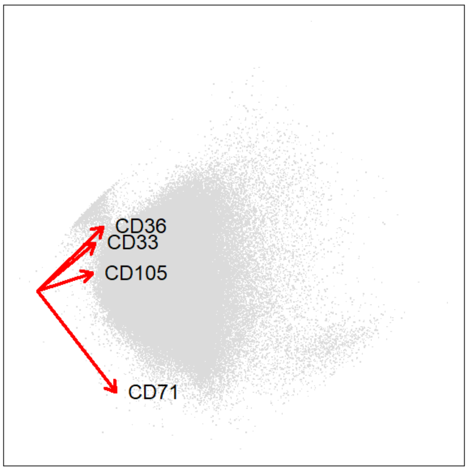

Tube 4

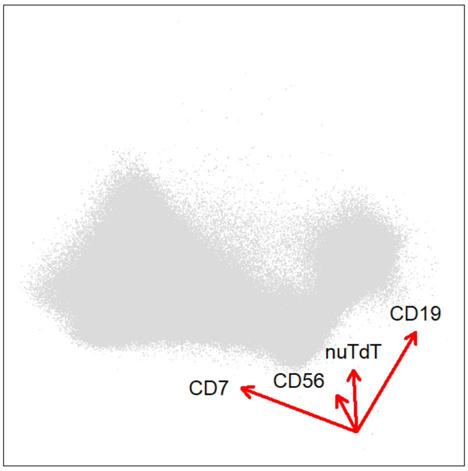

Tube 5

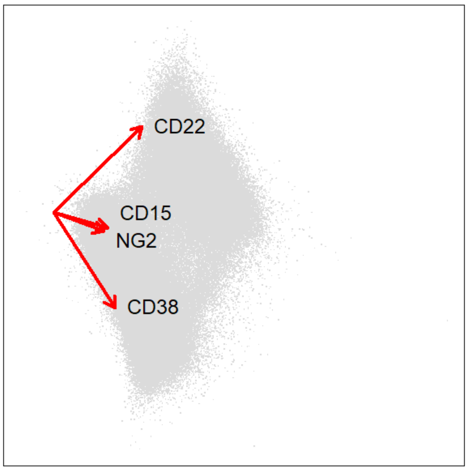

Tube 6

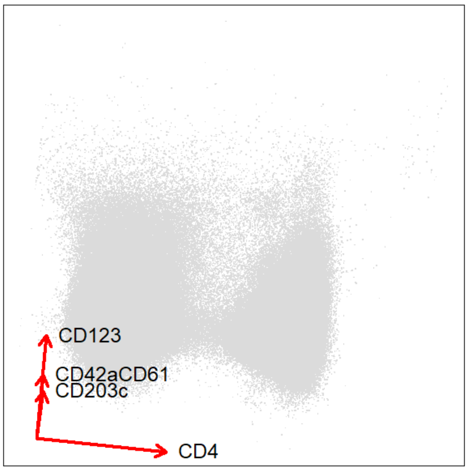

Result – Loadings of variables

Like previously, the contributions of individual markers were visualized in biplots (details in **Supplementary Data S6**):

Tube 1	PC1	PC2
CD16	49.1 %	23.3 %
CD13	7.0 %	22.1 %
CD11b	37.6 %	20.8 %
CD10	6.3 %	33.8 %

Tube 2	PC1	PC2
CD35	55.6 %	22.4 %
CD64	10.7 %	17.0 %
CD300e	14.1 %	23.5 %
CD14	19.6 %	37.1 %

Tube 3	PC1	PC2
CD36	25.5 %	27.8 %
CD105	21.4 %	7.5 %
CD33	22.6 %	20.7 %
CD71	30.5 %	43.9 %

Tube 4	PC1	PC2
nuTdT	1.6 %	25.6 %
CD56	9.8 %	15.2 %
CD7	58.4 %	18.0 %
CD19	30.2 %	41.2 %

Tube 5	PC1	PC2
CD15	21.3 %	7.1 %
NG2	19.8 %	8.6 %
CD22	34.8 %	40.0 %
CD38	24.1 %	44.3 %

Tube 6	PC1	PC2
CD42aCD61	4.4 %	28.0 %
CD203c	4.5 %	20.8 %
CD123	6.3 %	45.3 %
CD4	84.9 %	5.9 %

Result – Loadings of variables

Each marker contributed significantly (at least 15% to either of the components, and at least 12% cumulatively).

Supplementary Data S11 – Check cases against TS Reference Region

For each case, each tube was checked against the appropriate TS reference region (**Supplementary Data S10**). Thus, for each tube, the percentage of lymphocytes inside the TS reference region was calculated, resulting in the following matrix (16 out of 699 cases shown, where “TS.AML.1” to “TS.AML.6” gives the percentage for tube 1 to 6 respectively):

CASE	TS.AML.1	TS.AML.2	TS.AML.3	TS.AML.4	TS.AML.5	TS.AML.6	MEDIAN
F.062*	0.99	0.98	0.99	0.99	0.96	0.99	98.7
G.166	0.99	0.99	0.98	0.94	0.99	1.00	98.8
E.026*	0.99	0.98	0.97	0.97	0.98	0.99	98.0
C.055	0.99	0.97	0.98	0.98	0.98	0.99	98.1
B.043*	0.91	0.90	0.96	0.95	0.95	0.96	95.12
A.001	0.96	0.96	0.92	0.93	0.95	0.95	95.1
G.204*	0.92	0.91	0.90	0.90	0.87	0.89	90.0
F.271	0.91	0.87	0.95	0.91	0.82	0.92	90.7
F.244*	0.94	0.89	0.84	0.89	0.85	0.89	88.9
A.002	0.94	0.90	0.84	0.92	0.83	0.85	87.5
G.170*	0.75	0.73	0.57	0.68	0.99	0.90	73.7
A.019	0.52	0.09	0.67	0.76	0.79	0.75	70.9
F.115*	0.66	0.81	0.67	0.59	0.72	0.37	66.27
G.014	0.53	0.11	0.65	0.76	0.97	0.63	63.97
A.015	0.15	0.15	0.47	0.75	0.76	0.46	46.52
A.030	0.35	0.22	0.43	0.72	0.74	0.56	49.35

In this study, tubes with less than 80 percent of their lymphocytes within the TS reference region were considered “out-of-reference” (indicated by red color). Cases with one or more tubes “out of reference” were marked, resulting in 146 out of 574 cases being flagged (details in **Supplementary Data S12**). For various cases (as indicated by asterisk), the calculation is visualized on the next page (tube 1 up to 6 indicated by T1 up to T6 respectively).

F.062 (median 98.7 percent)

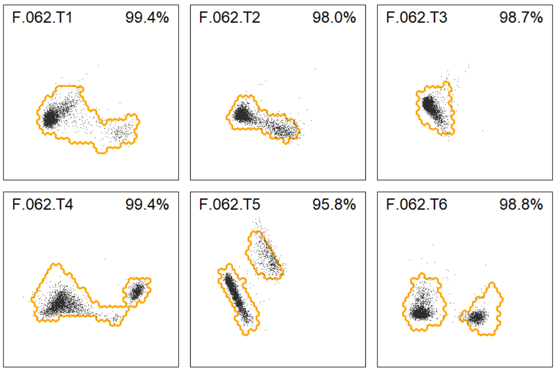

E.026 (median 98.0 percent)

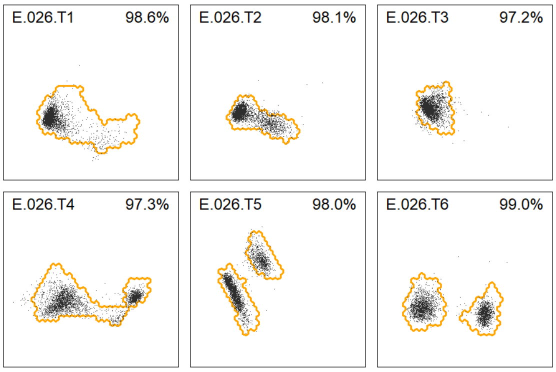

B.043 (median 95.12 percent)

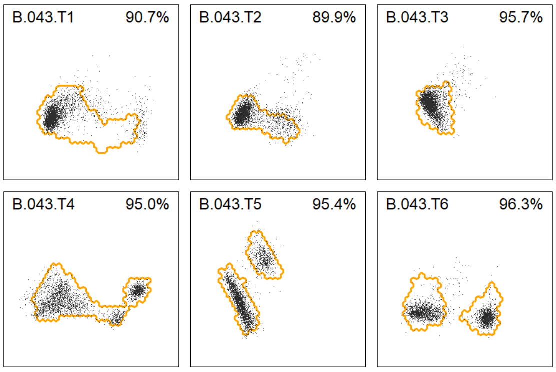

G.204 (median 90.0 percent)

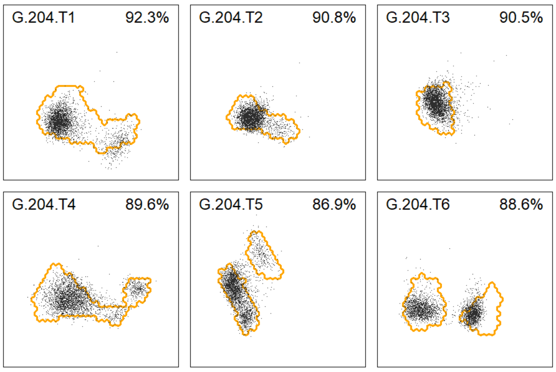

F.244 (median 88.9 percent)

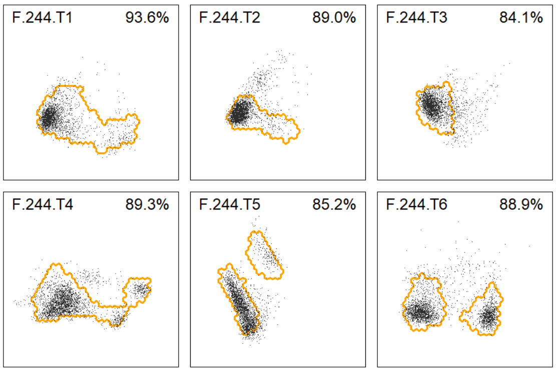

G.170 (median 73.7 percent)

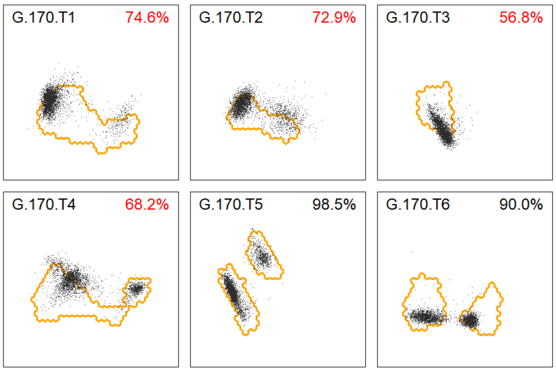

F.115 (median 66.27 percent)

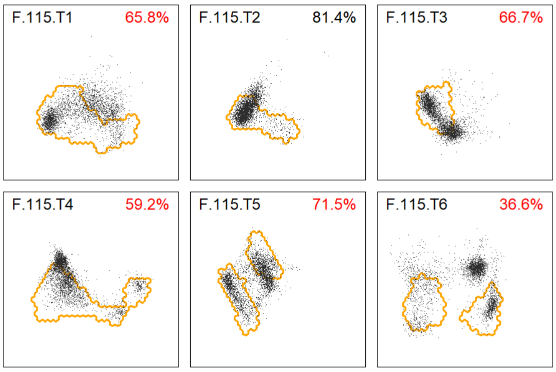

A.015 (median 46.52 percent)

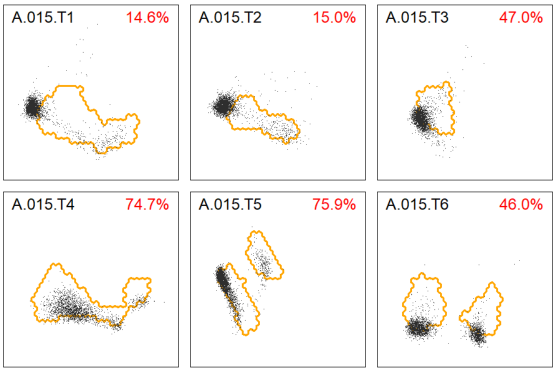

Supplementary Data S12 – Review of marked cases (TS)

By checking against the TS reference region (i.e. "the PCA based approach"), in total 146 cases were marked (details in **Supplementary Data S9, 10 & 11**). Whether these cases deviated in any meaningful way from the cohort average, was evaluated by comparing "the PCA based approach" against "the median/peak based approach". This review procedure was similar to the review procedure for cases that were excluded based on the BB reference region (details in **Supplementary Data S8**), with the only difference being the markers (i.e. BB versus TS markers)

Visualization

During the BB evaluations, median intensities were used in case of uni-modality (e.g. CD34, CD45 and CD117) while peak intensities where used in case of bi-modality (e.g. HLADR), for various reasons (details in **Supplementary Data S5**). During the TS evaluations, the same strategy was applied. Twenty-four TS markers were evaluated, of which six (CD4, CD7, CD16, CD19, CD22 and CD35) were considered to be bi-modal (assuming normal lymphocytes). For these six markers, the peaks were detected (examples below). For the other markers, the median was used.

CD4 *(for G.157 and H.011)*

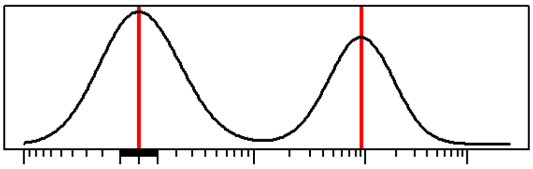

CD7 *(for A.016 and F.167)*

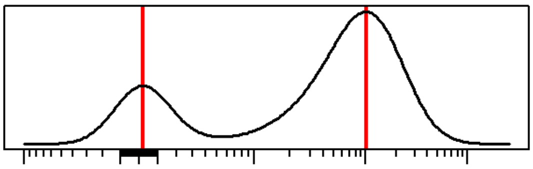

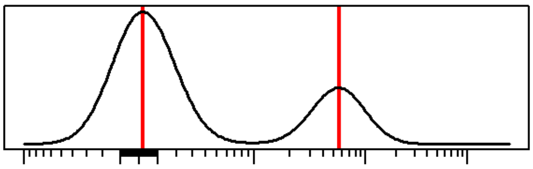

CD16 *(for F.027 and E.022)*

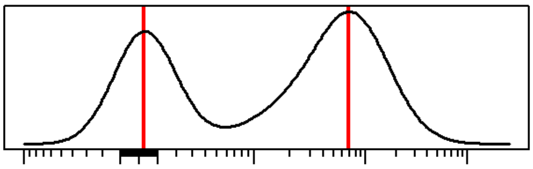

CD19 *(for A.016 and F.167)*

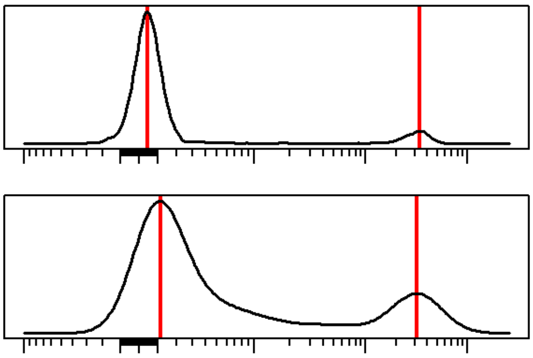

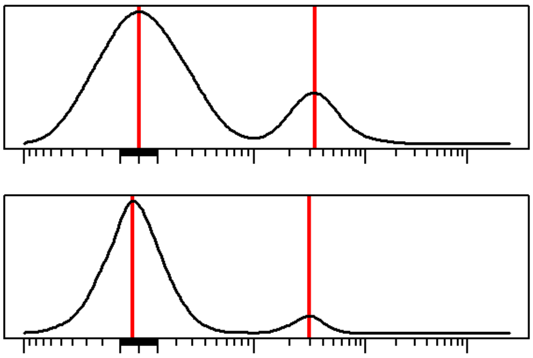

CD22 *(for H.005 and E.022)*

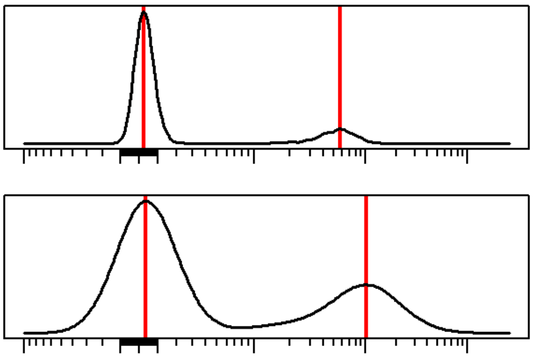

CD35 *(for A.31 and F.198)*

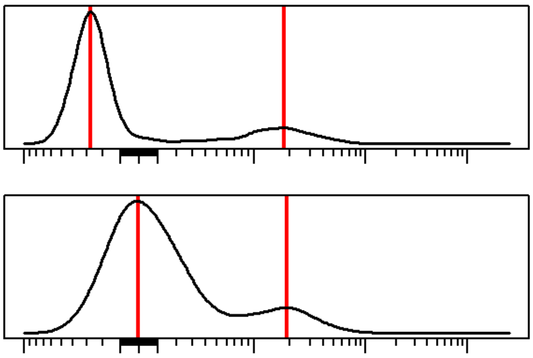

Visualization

The aggregated median/peak intensities (for each case, each tube, each TS parameter):

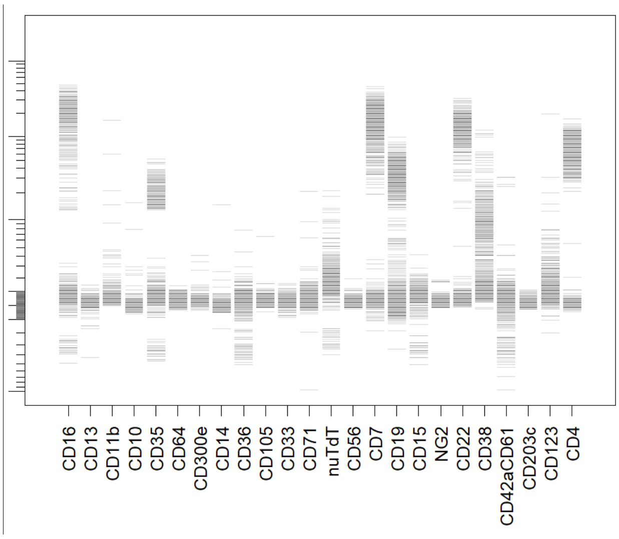

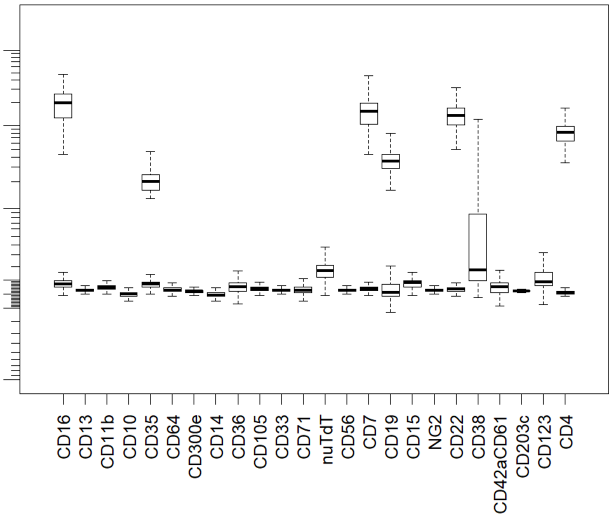

Review

For each case, the plot with median/peak intensities (on top of the aggregated data) was combined with the previously generated TS reference plot (details in **Supplementary Data S7**). This way, the “the PCA based approach” can be easily compared against the “the median/peak based approach”.

Cases within TS reference region

Six exemplary cases, with the vast majority of their lymphocytes within the TS reference region:

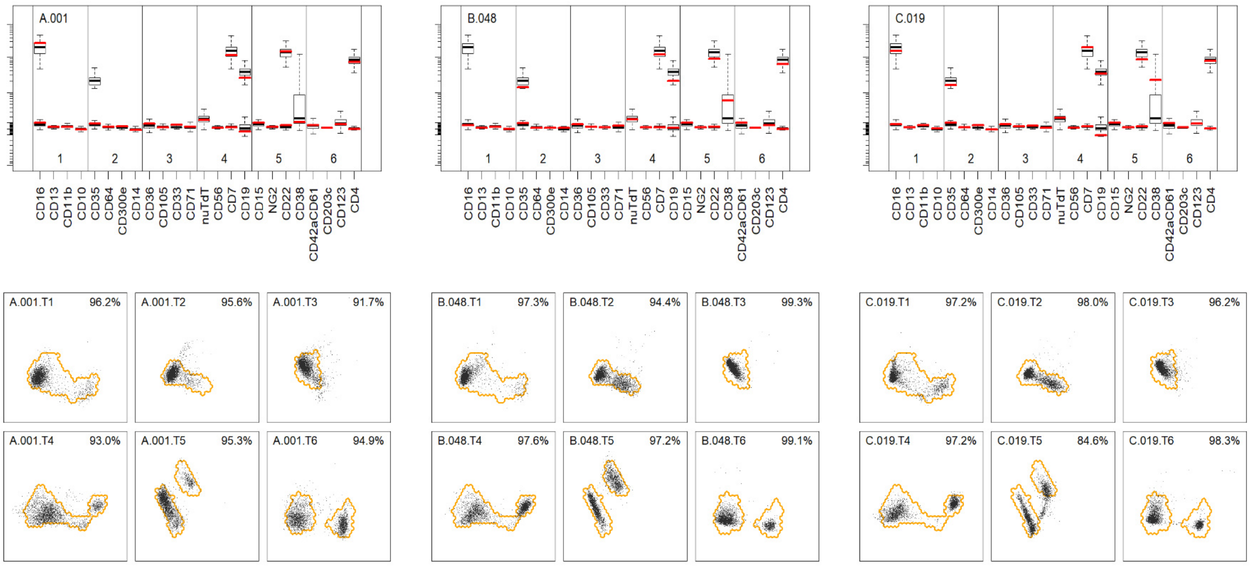

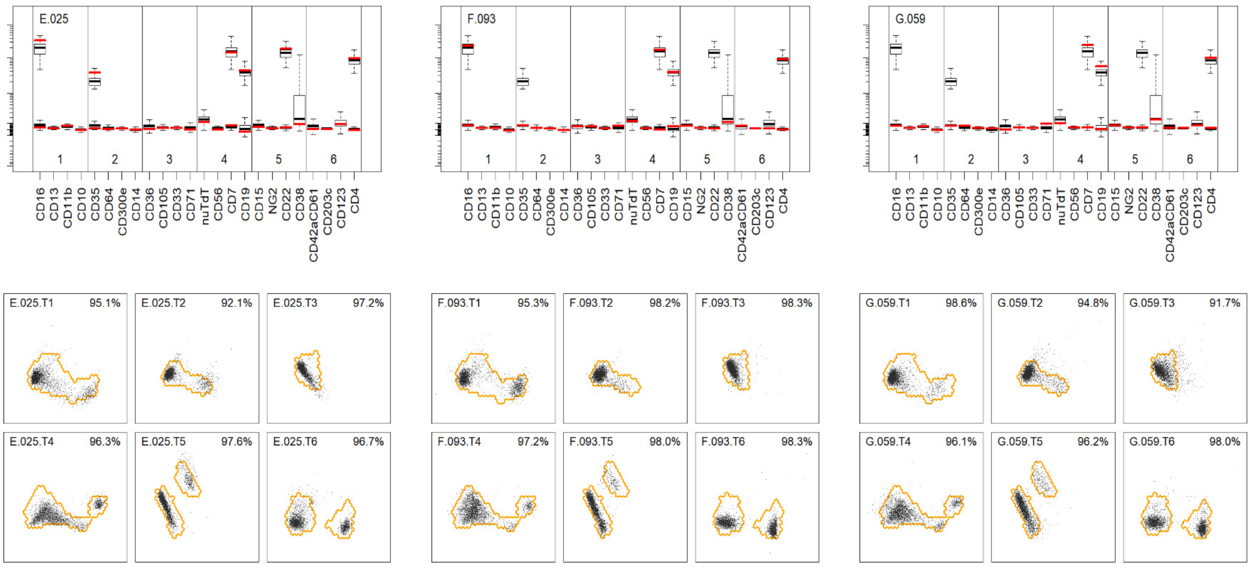

E.025
F.093
G.059
1 2 3 4 5 6
CD16 CD13 CD11b CD10 CD35 CD64 CD300e CD14 CD36 CD105 CD33 CD71 nuTdT CD56 CD7 CD19 CD15 NG2 CD22 CD38 CD42aCD61 CD203c CD123 CD4
E.025.T1 95.1%
E.025.T2 92.1%
E.025.T3 97.2%
E.025.T4 96.3%
E.025.T5 97.6%
E.025.T6 96.7%
F.093.T1 95.3%
F.093.T2 98.2%
F.093.T3 98.3%
F.093.T4 97.2%
F.093.T5 98.0%
F.093.T6 98.3%
G.059.T1 98.6%
G.059.T2 94.8%
G.059.T3 91.7%
G.059.T4 96.1%
G.059.T5 96.2%
G.059.T6 98.0%

Cases outside TS reference region

Based on the previously established exclusion criteria (one or more tubes with less than 80 percent of lymphocytes within reference, details in **Supplementary Data S10 & 11**), in total 146 out of 574 cases were excluded. These 146 cases could be categorized in various groups:

- One marker within one tube being out-of-reference (n=88);
- Within each tube, one marker at a certain channel being out-of-reference (e.g. the FITC channel) (n=21);
- Within multiple tubes, one marker at a certain channel being out-of-reference (n=10);

Within multiple tubes, one marker being out of reference (regardless of channel) (n=8);

Other (seemingly random) patterns, in terms of marks being out-of-reference (n=19).

Examples of each category are shown below:

- One marker within one tube being out-of-reference (n=88):

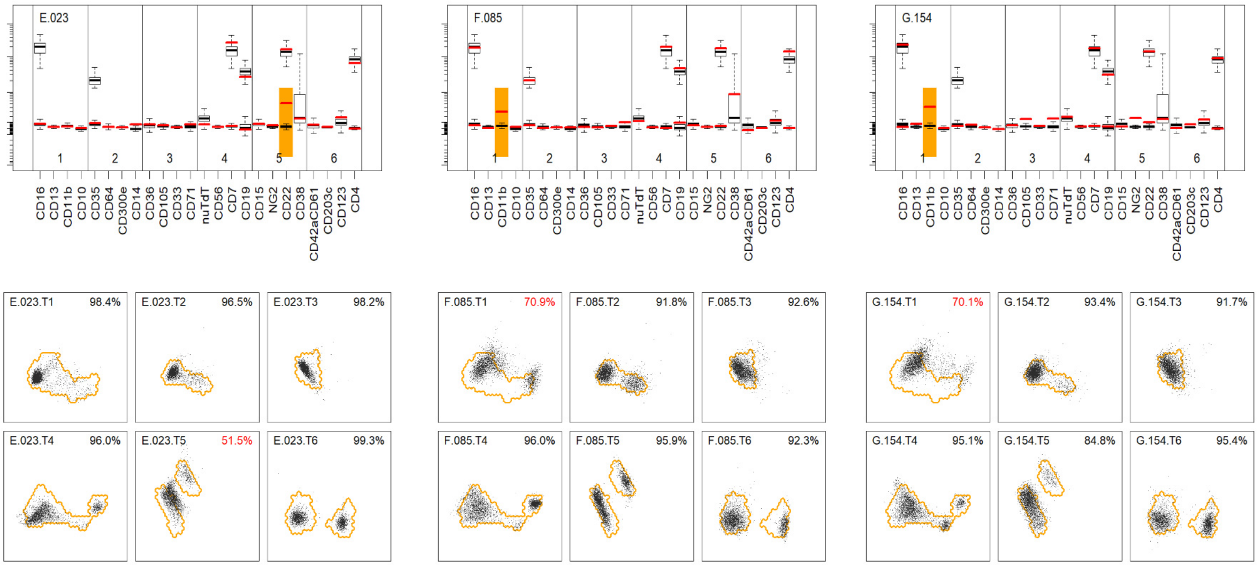

- Within each tube, one marker at a certain channel being out-of-reference (e.g. the FITC channel) (n=21):

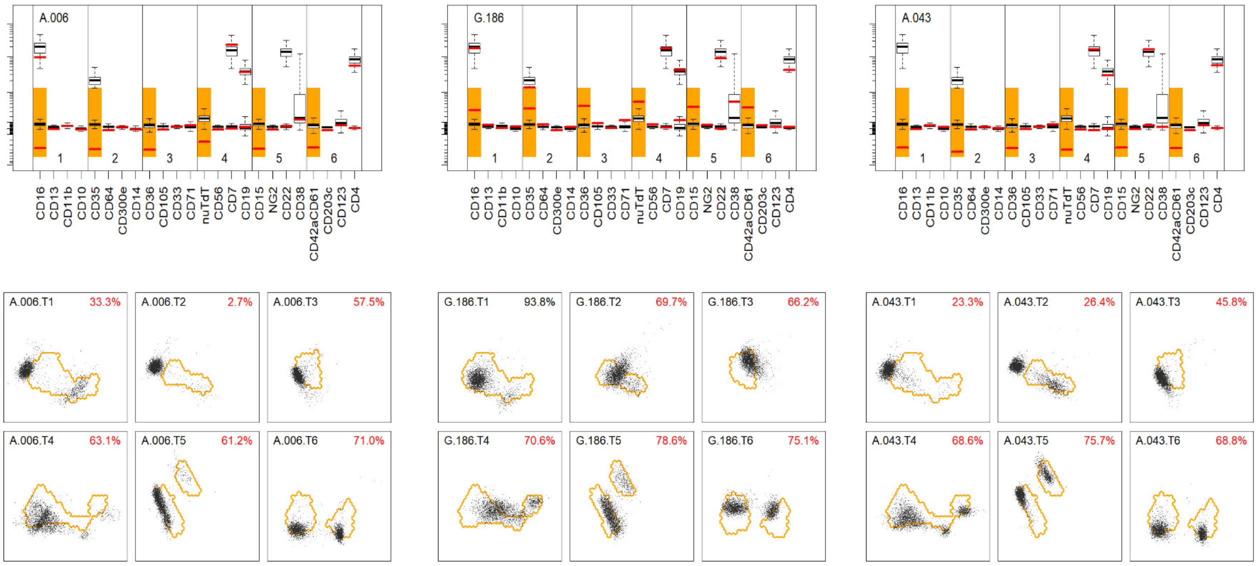

- Within multiple tubes, one marker at a certain channel being out-of-reference (n=10):

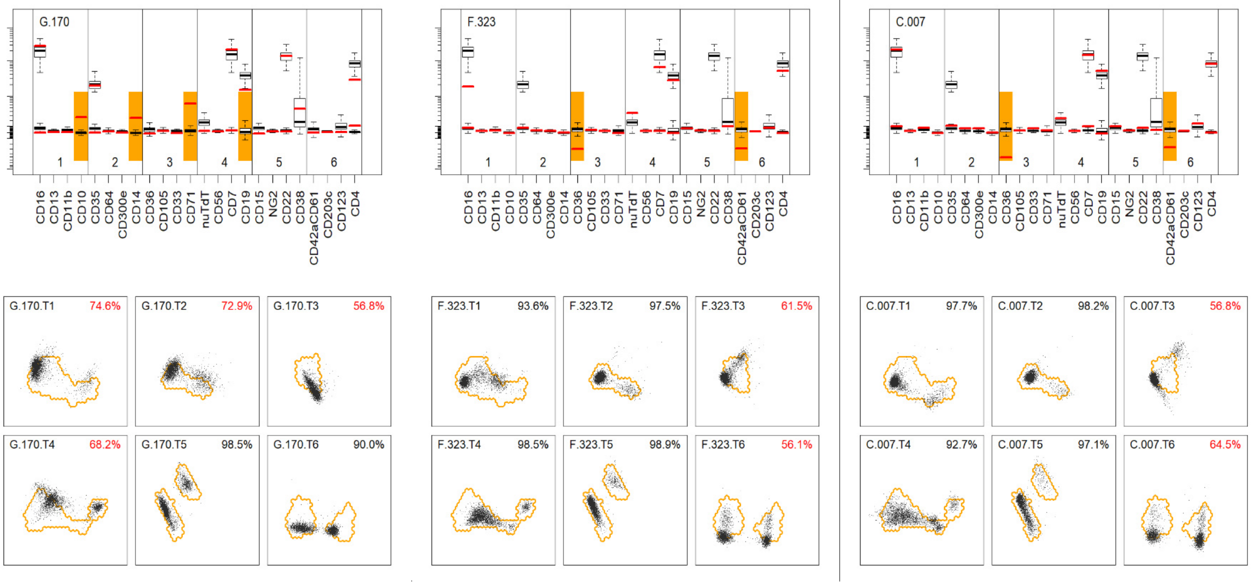

Within multiple tubes, one marker being out of reference (regardless of channel) (n=8):

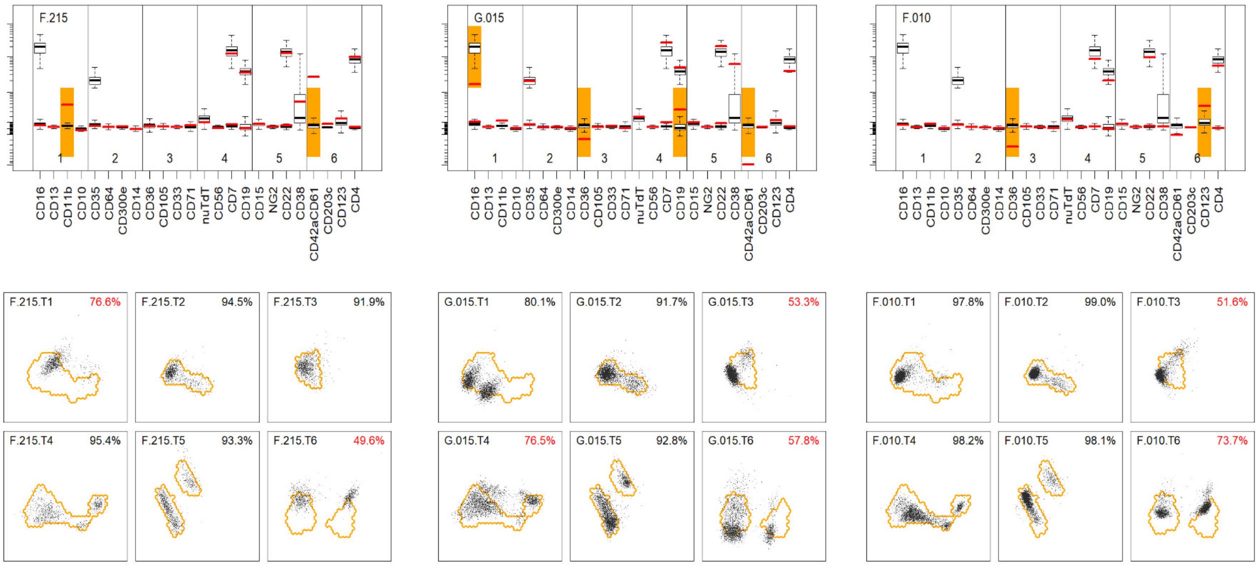

Other (seemingly random) patterns, in terms of marks being out-of-reference (n=19):

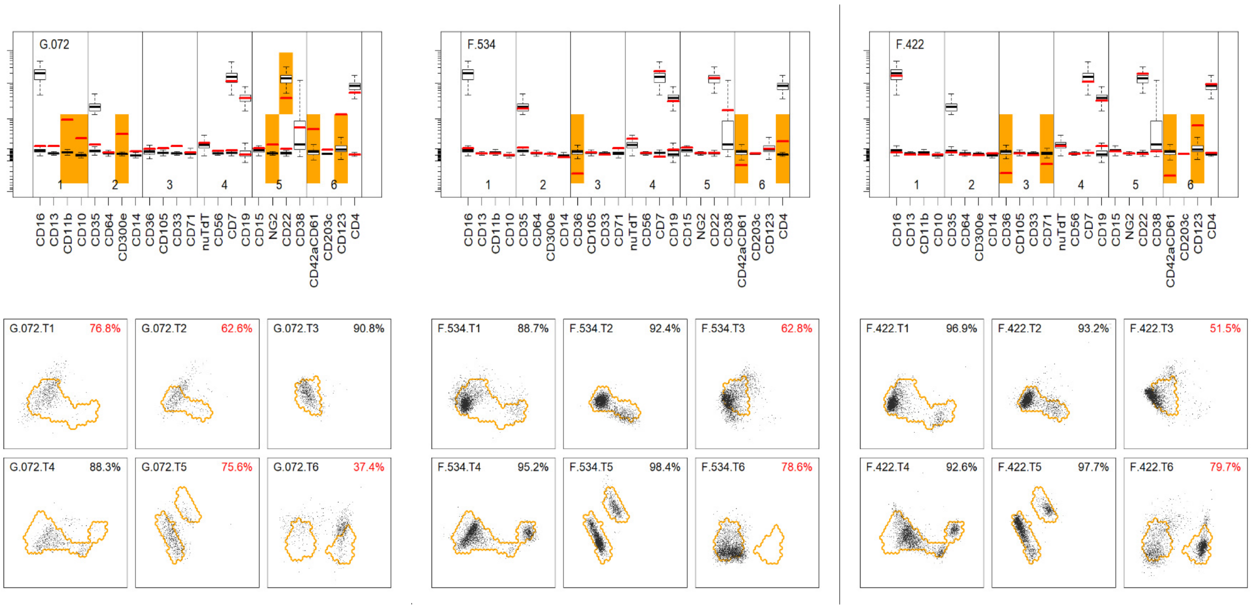

Some patterns were found across centers, while other patterns exclusively occurred within one center:

Same pattern (in terms of markers out-of-range) in cases from different centers:

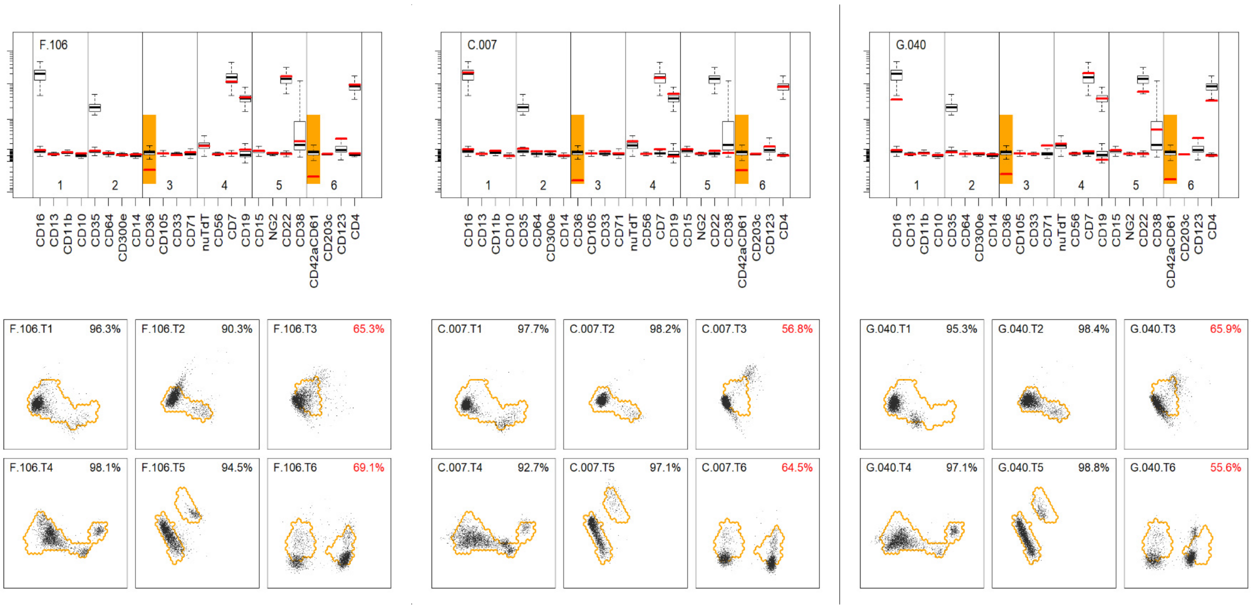

Same pattern (in terms of markers out-of-range) in cases from the same centers:

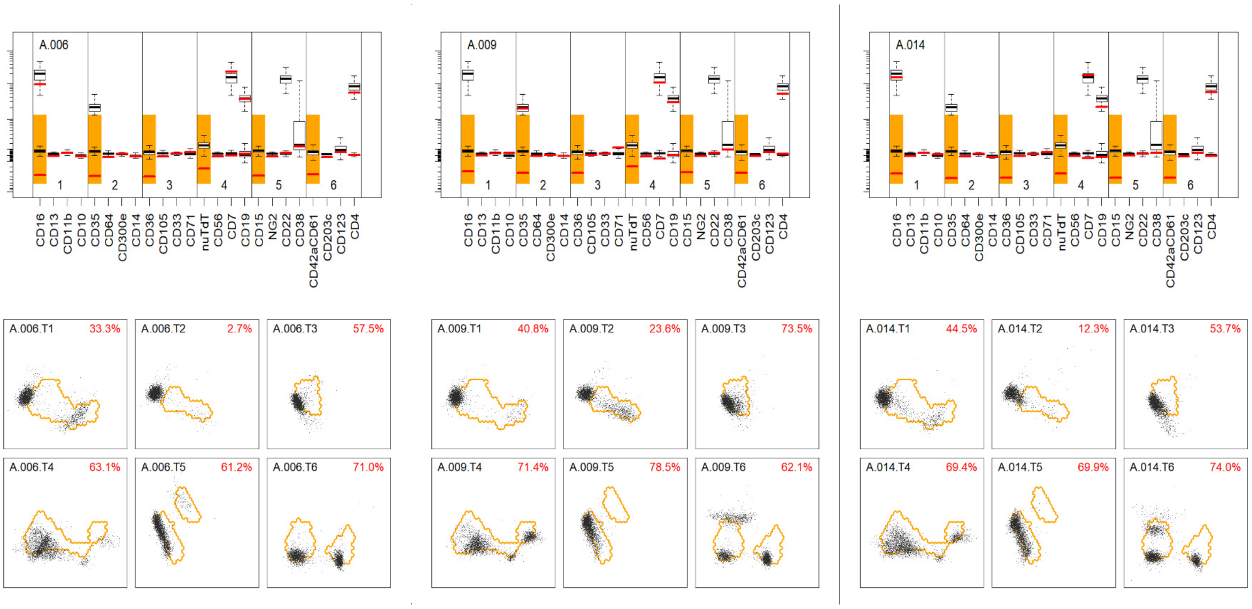

Notably, the PCA approach detected various abnormalities that were missed by the median/peak approach. For example, the tri-modal CD22 expression in case B.049 was not detected by the median/peak approach (i.e. only the extreme/outer peaks were taken into account, see top left panel), while it was detected by the PCA approach (i.e. the portion of lymphocytes with intermediate CD22 expression was out-of-reference, see bottom left panel). As another example, the increased standard deviation for CD35 in case G.202 was not detected by the median/peak approach (i.e. peak intensities was not affected, see top right panel), while it was detected by the PCA approach (i.e. as the extremities were out-of-reference).

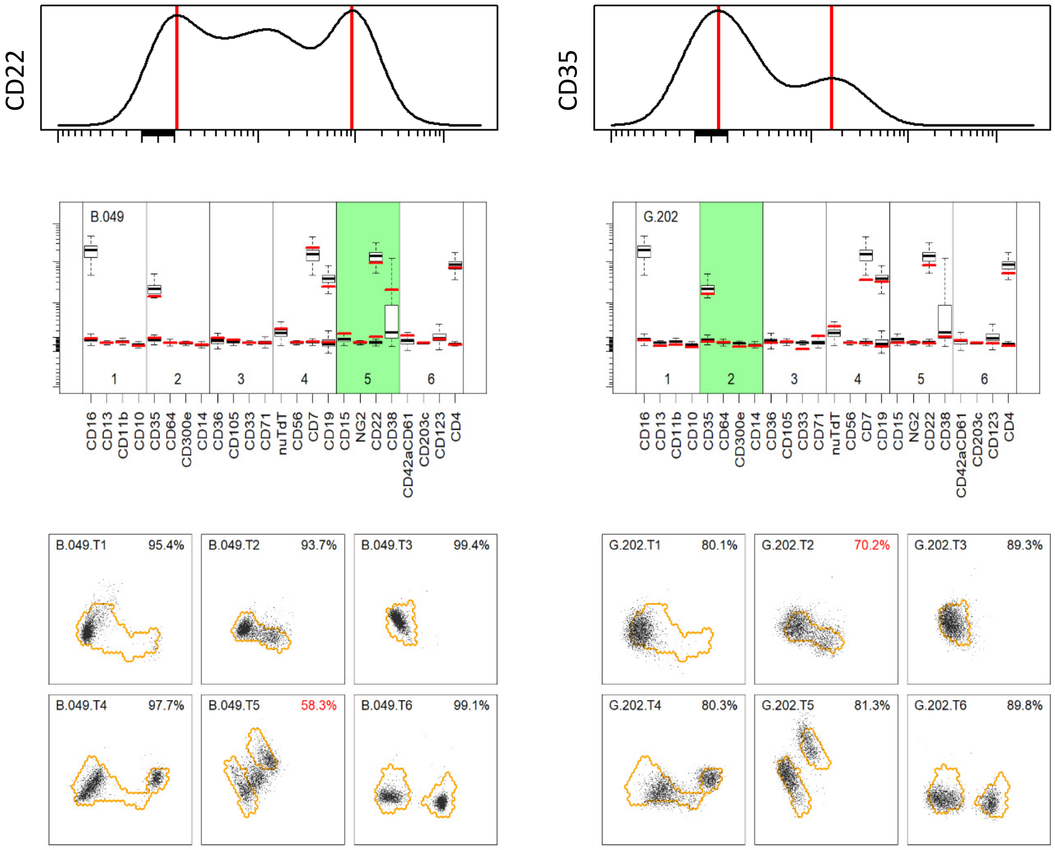

For clarity, the corresponding histograms, from cases within-reference:

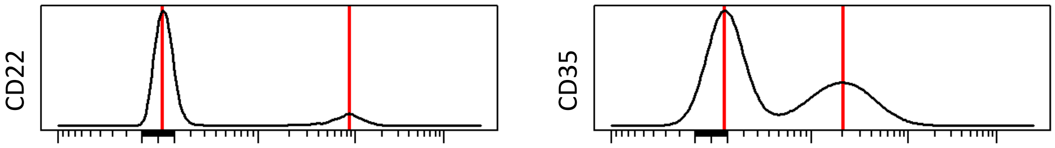

Consistency

Notably, the PCA approach and the median/peak approach showed very strong correlations, in terms of being able to detect the same abnormalities (i.e. certain markers/tubes being out-of-reference).

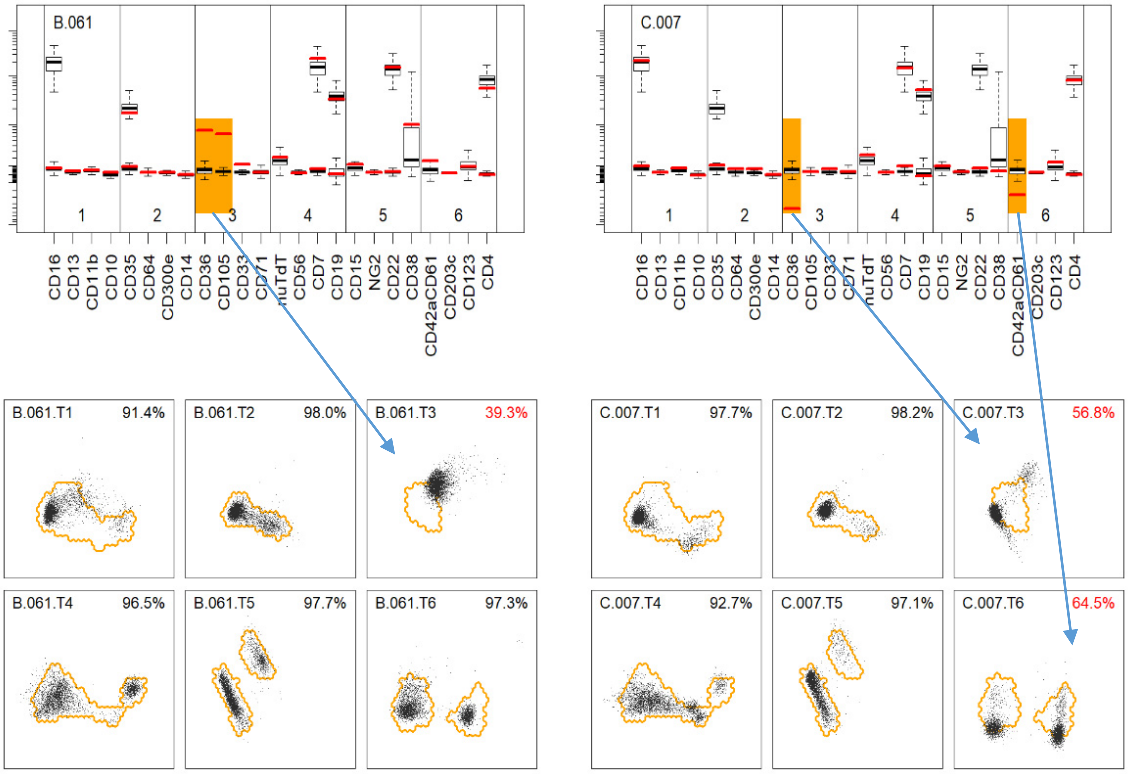

There were however a few situations in which there as a discrepancy between the PCA-based and median/peak-based approach. These could be grouped in three:

1. Cases where the HLADR negative fraction was "too-variable" according to the median/peak-based approach, but "within-reference" according to the PCA-based approach (n=4):

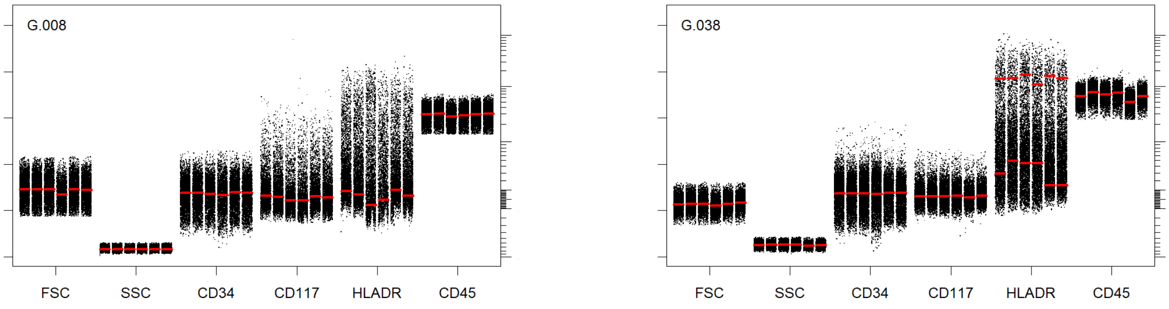

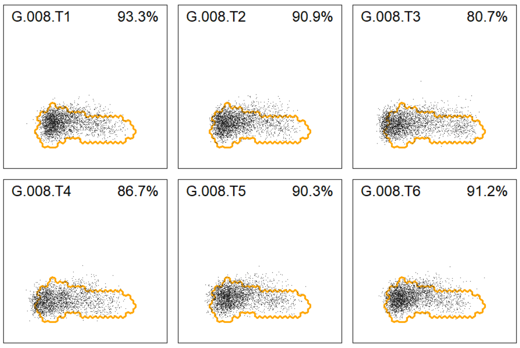

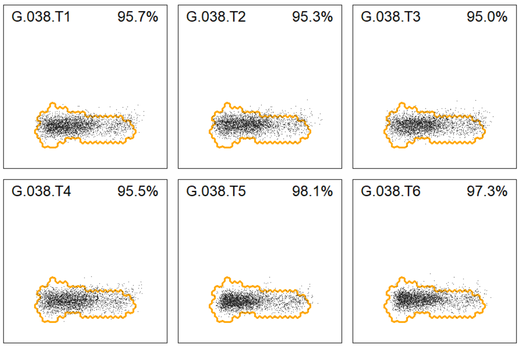

2. Cases where FSC was “too-variable” according to the median/peak-based approach, but “within-reference” according to the PCA-based approach (n=8):

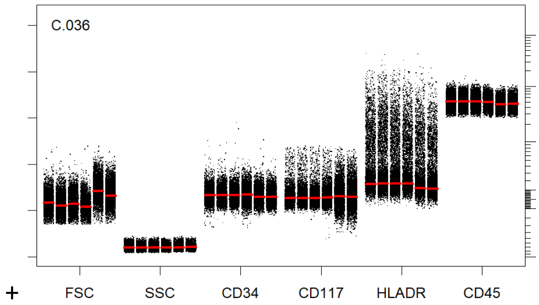

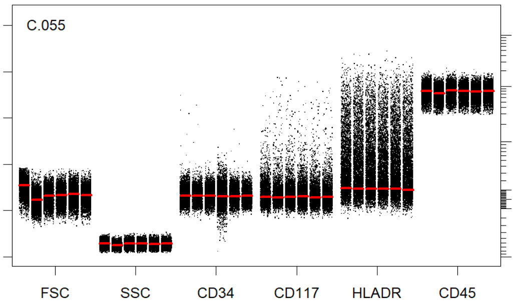

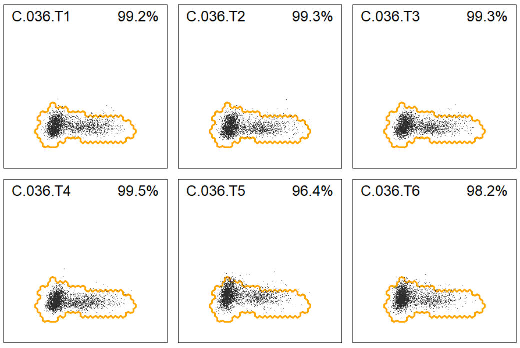

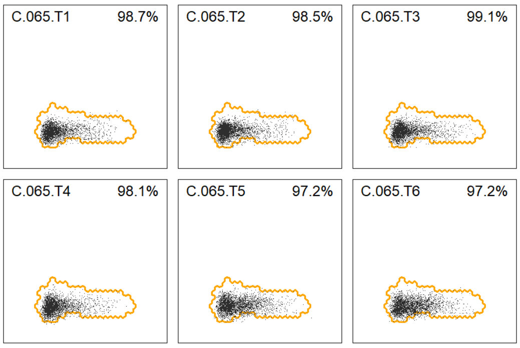

3. Cases where the median/peak-based approach showed significant differences between the tubes, but were “within-reference” according to the PCA-based approach (n=73). These can be explained by no-reliable median/peak values, due to too few HLA-DR positive events being present (very low peak in histogram)

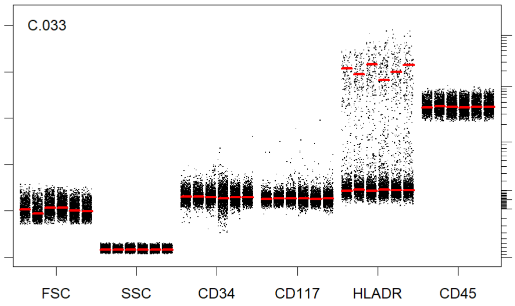

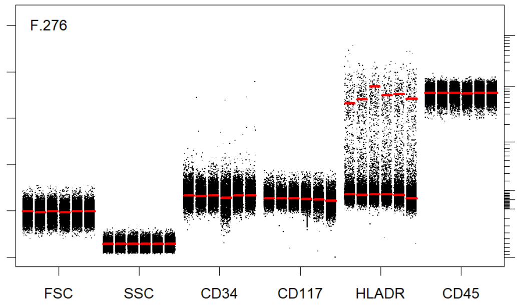

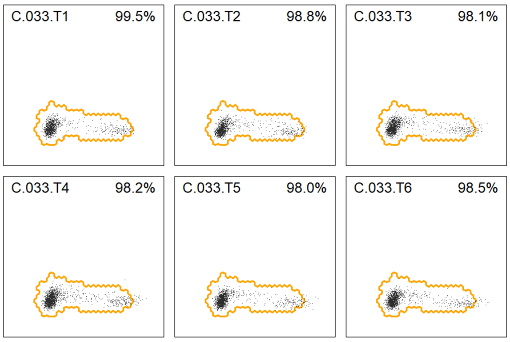

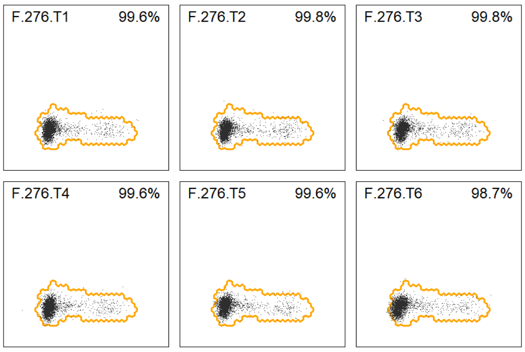

Explainability

Notably, by reviewing the PCA plots, one can easily identify positive deviations (e.g. CD11b as shown at top) or positive deviations (e.g. CD42aCD61 as shown at bottom). In other words, the PCA plots preserve directionality.

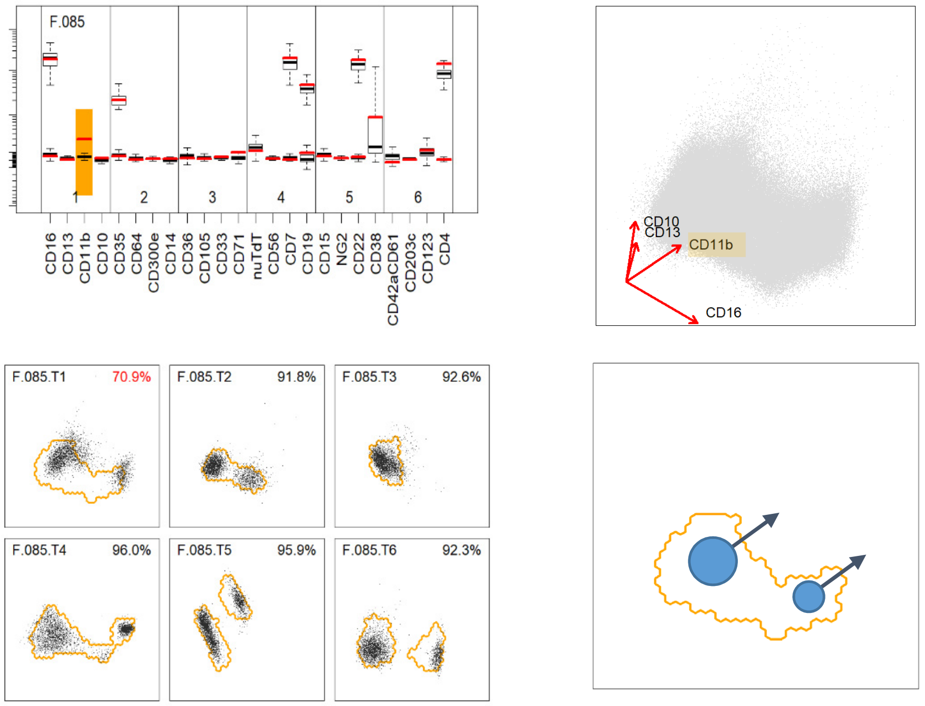

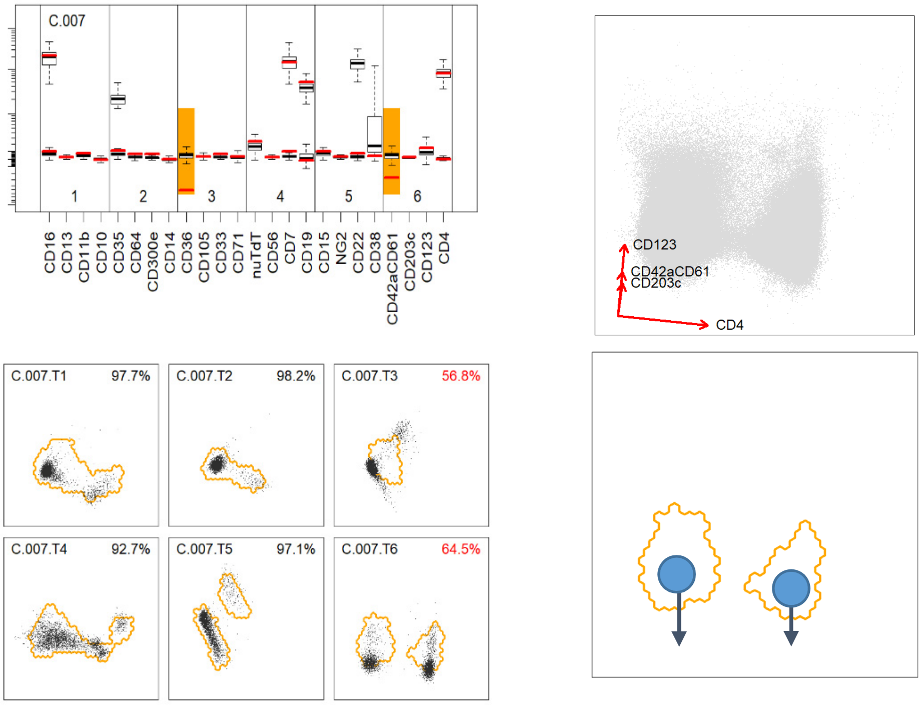
C.007
1
2
3
4
5
6
CD16
CD13
CD11b
CD10
CD35
CD64
CD300e
CD14
CD36
CD105
CD33
CD71
nuTdT
CD56
CD7
CD19
CD15
NG2
CD22
CD38
CD42aCD61
CD203c
CD123
CD4
C.007.T1
97.7%
C.007.T2
98.2%
C.007.T3
56.8%
C.007.T4
92.7%
C.007.T5
97.1%
C.007.T6
64.5%
CD123
CD42aCD61
CD203c
CD4

Supplementary Data S13 – Details of technically flagged cases

Despite standardized sample preparations and instrument settings, still important technical differences were noted in a considerable number of cases, which were flagged because of lymphocytes being out of the reference region for either the BB or TS markers. To better understand the reasons why such cases were flagged, we evaluated these cases in more detail.

Flagged cases vary per center

The percentage of cases flagged during the BB QA or TS QA were analyzed per center and showed clear differences:

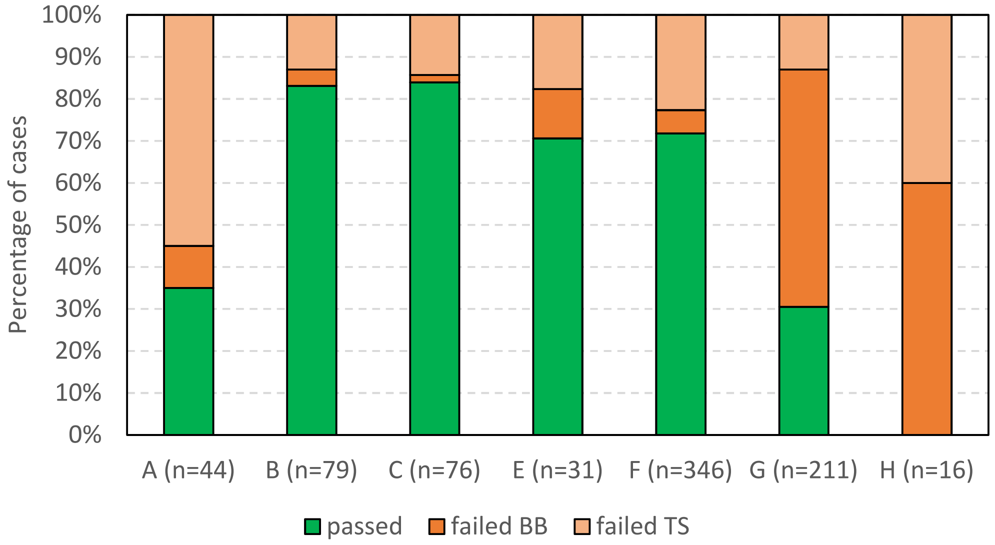

Cases flagged in the BB QA

For BB issues, it is expected that the deviations are seen in all 6 tubes. Indeed, analysis of the flagged showed that in the vast majority of flagged cases all 6 tubes showed deviations:

Percentage of tube flagged in total of flagged cases							
center	n	BB1	BB2	BB3	BB4	BB5	BB6
A	4	50	75	75	100	75	75
B	3	100	100	100	100	100	100
C	1	100	100	100	100	100	100
E	2	100	50	100	0	0	0
F	19	79	74	79	84	68	84
G	87	55	54	51	89	53	55
H	9	89	89	89	89	89	89

Center G and H show relatively high numbers of samples with BB issues, which appeared to be related to the use of reagents other than the reference reagents (especially CD45).

Cases flagged in the TS QA

For TS issues, it is expected that the deviations are seen in one tube only, unless the issue is not related to the antibody but to more general settings. Indeed, analysis of the flagged showed that in the vast majority of flagged cases all only one tube showed deviations, with the exception of center A:

Percentage of tube flagged in total of flagged cases							
center	n	TS1	TS2	TS3	TS4	TS5	TS6
A	22	95	95	95	95	95	100
B	10	0	0	10	80	10	0
C	8	0	0	13	63	0	38
E	3	0	0	0	33	33	33
F	77	19	0	16	34	3	49
G	20	35	35	40	40	25	50
H	6	0	0	0	100	0	0

Center A and H showed relatively high numbers of samples with TS issues. In Center A compensation issues with the FITC signal were observed (which could be fixed easily, see Supplementary Data S14), and this explains why tis deviation was observed in all 6 tubes. In contrast, center H had mainly issues with tube 4 (including the intracellular TdT staining). Also in the other centers deviations in tube 4 were the most common reason for flagging the sample. In addition deviations in tube 6 were relatively frequently observed whereas tube 1, 2, 3, and 5 generally performed well. Note that tube 4 and 6 contained typical lymphocyte markers (CD19, CD7, CD56, CD4), and therefore deviations of the lymphocytes may become visible more easily.

Supplementary Data S14 – Fixes

In total 375 out of 803 cases were excluded, however, 60 out of 375 exclusions could be fixed with minimal effort:

Various cases from center A were excluded due to one specific compensation mistake (causing the FITC channel in each tube being out of reference). After correcting this specific compensation value (same value for each tube), in total 17 out of 23 additional cases passed every check. Three examples are shown below:

Before adjustment of compensation:

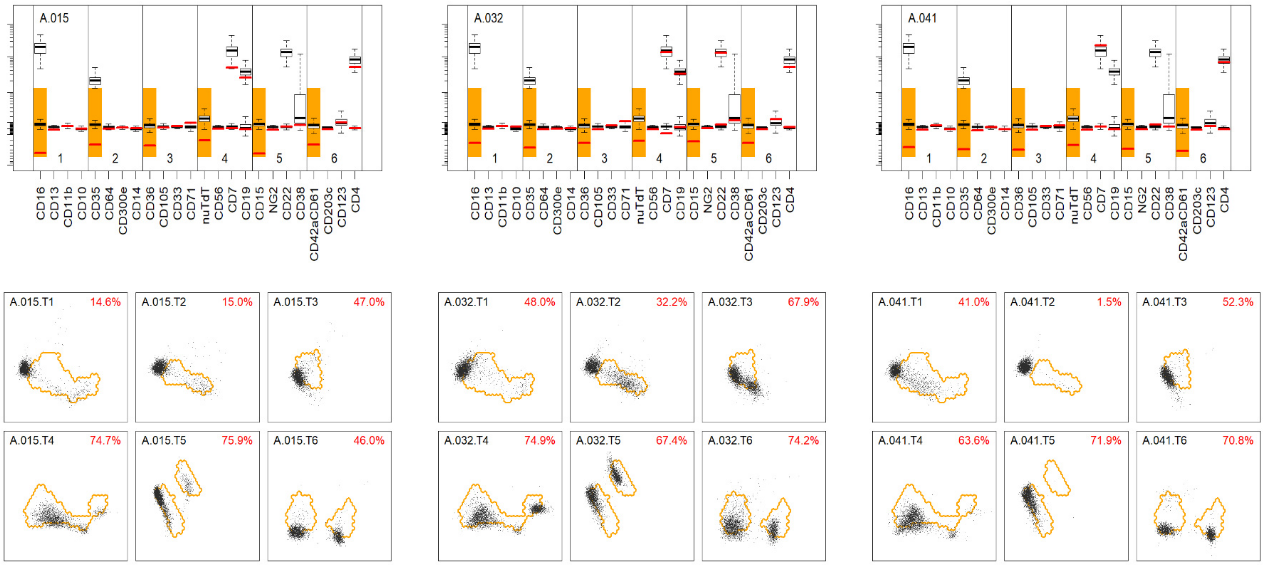

After adjustment of compensation:

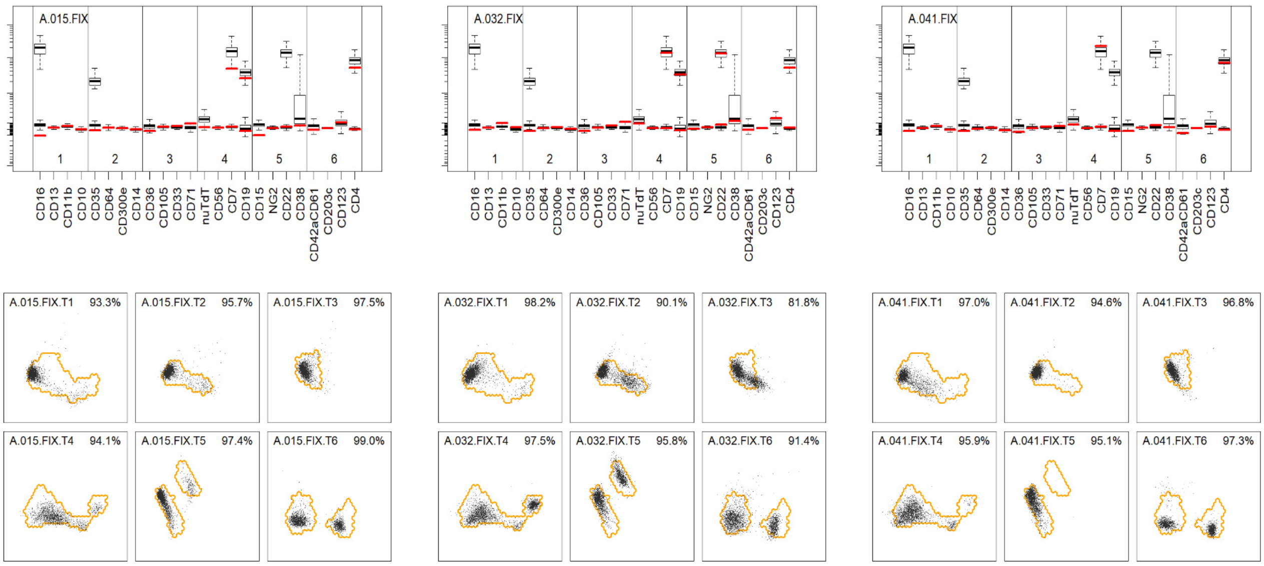

Likewise, various cases from center F were excluded, due to two specific compensation mistakes (CD19:APCH7 and CD42+CD61:FITC). After correcting these compensation values (same value for each case), in total 17 out of 17 additional cases passed every check.

Before adjustment of compensation (CD19:APCH7):

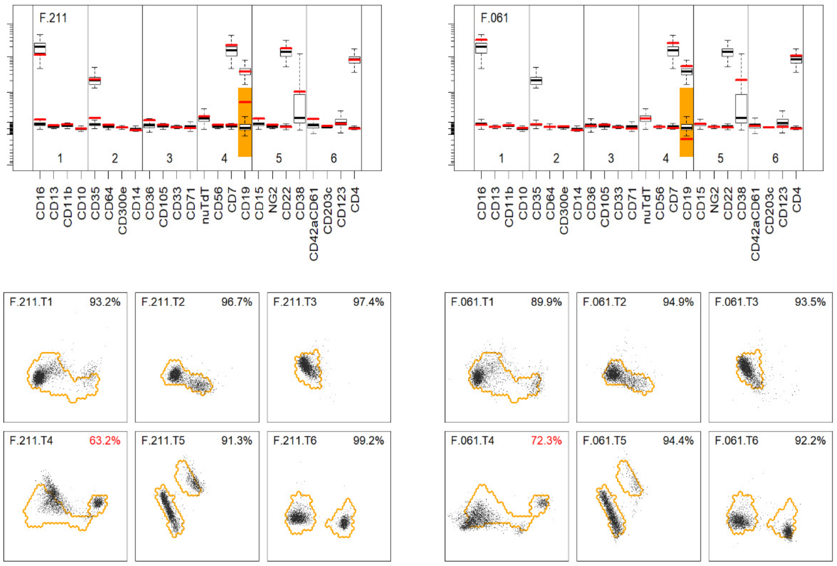

After adjustment of compensation (CD19:APCH7):

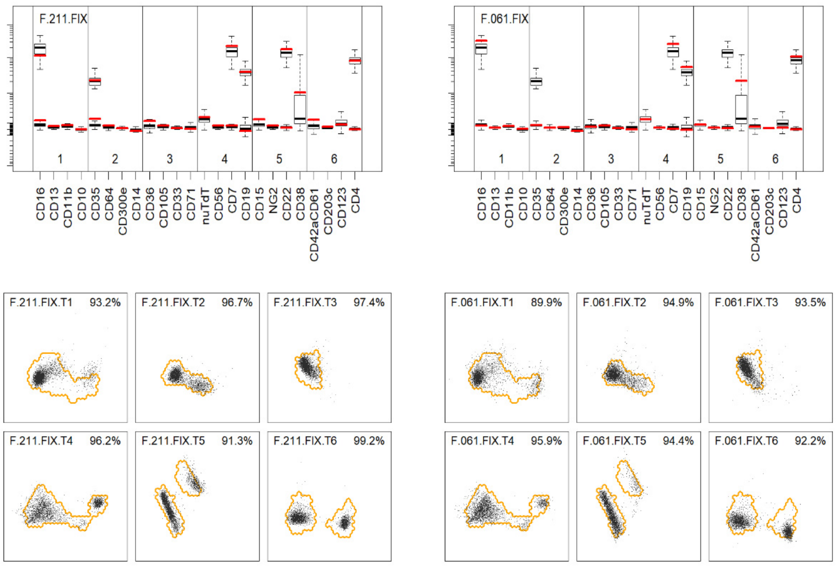

Before adjustment of compensation (CD42+CD61:FITC):

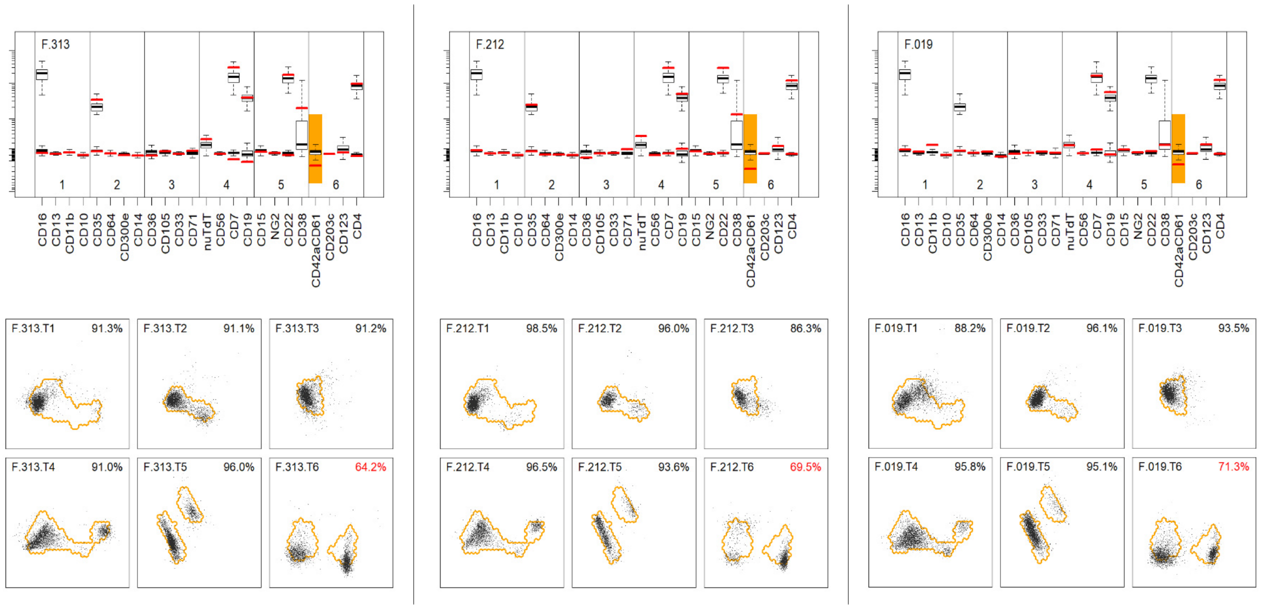

After adjustment of compensation (CD42+CD61:FITC):

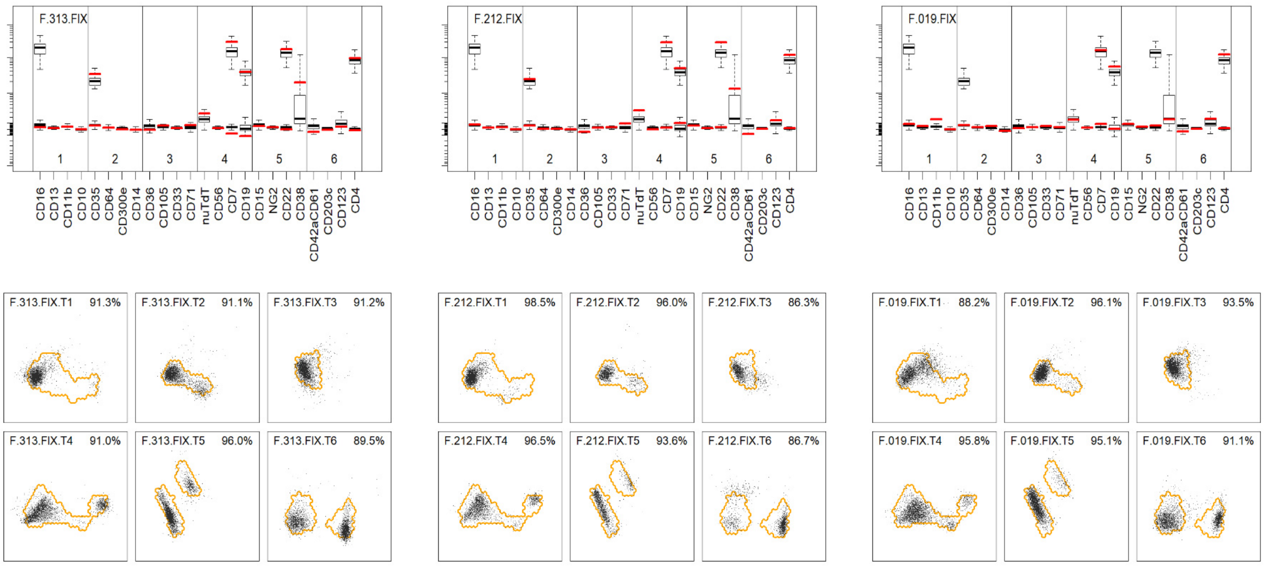

Finally, for ten cases for which initially no CYT file was uploaded and seven cases with an incomplete CYT file, a new CYT file was created. 7/17 cases passed each check after re-analysis.

Supplementary Data S15 – Final Results

By checking against the BB reference, 125 out of 699 cases were excluded (left before exclusion, right final cohort (n=472)):

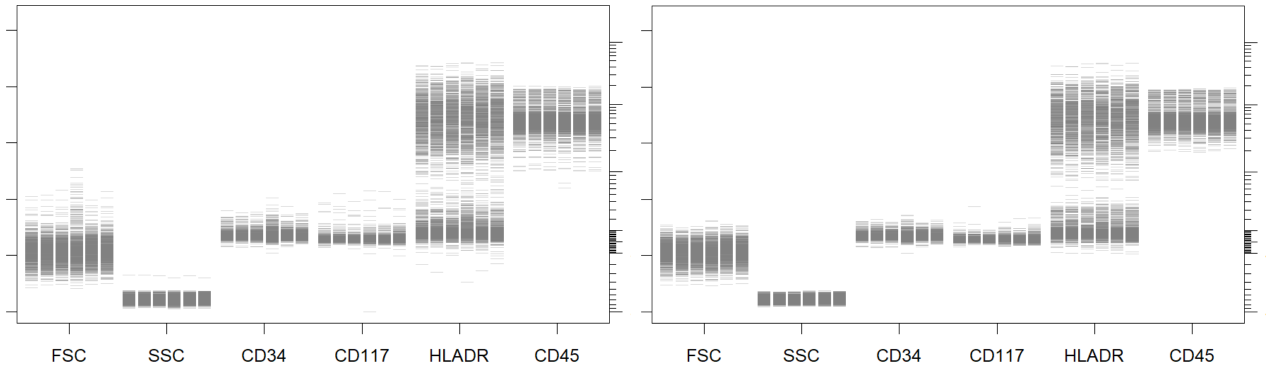

By checking against the TS reference, 146 out of 574 cases were excluded (left before exclusion, right final cohort (n=472)):

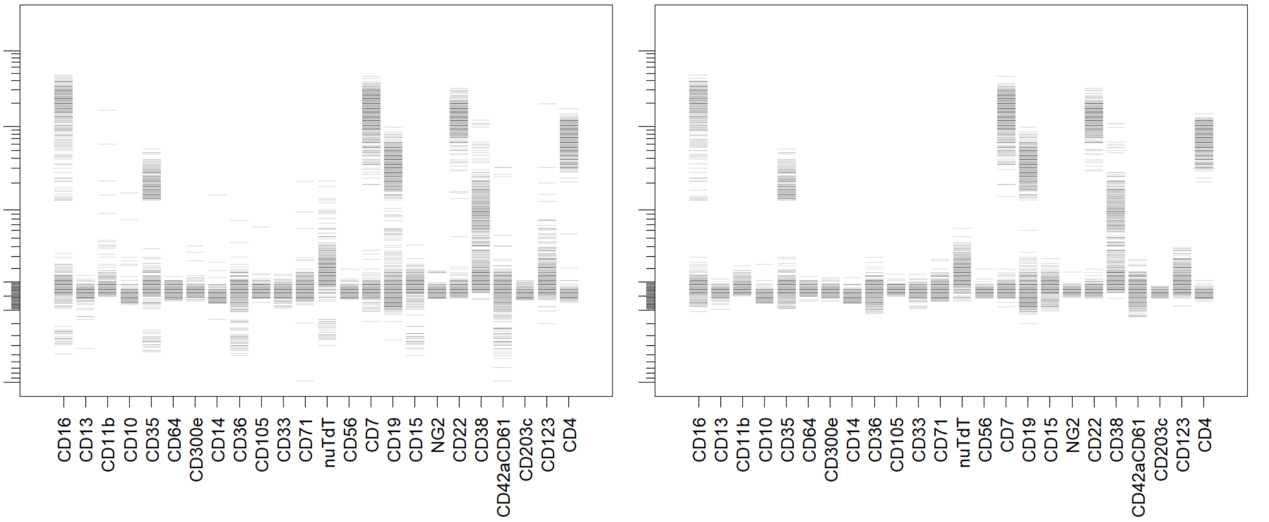

The overall data (n=472), per center and per marker, are shown in the histograms below:

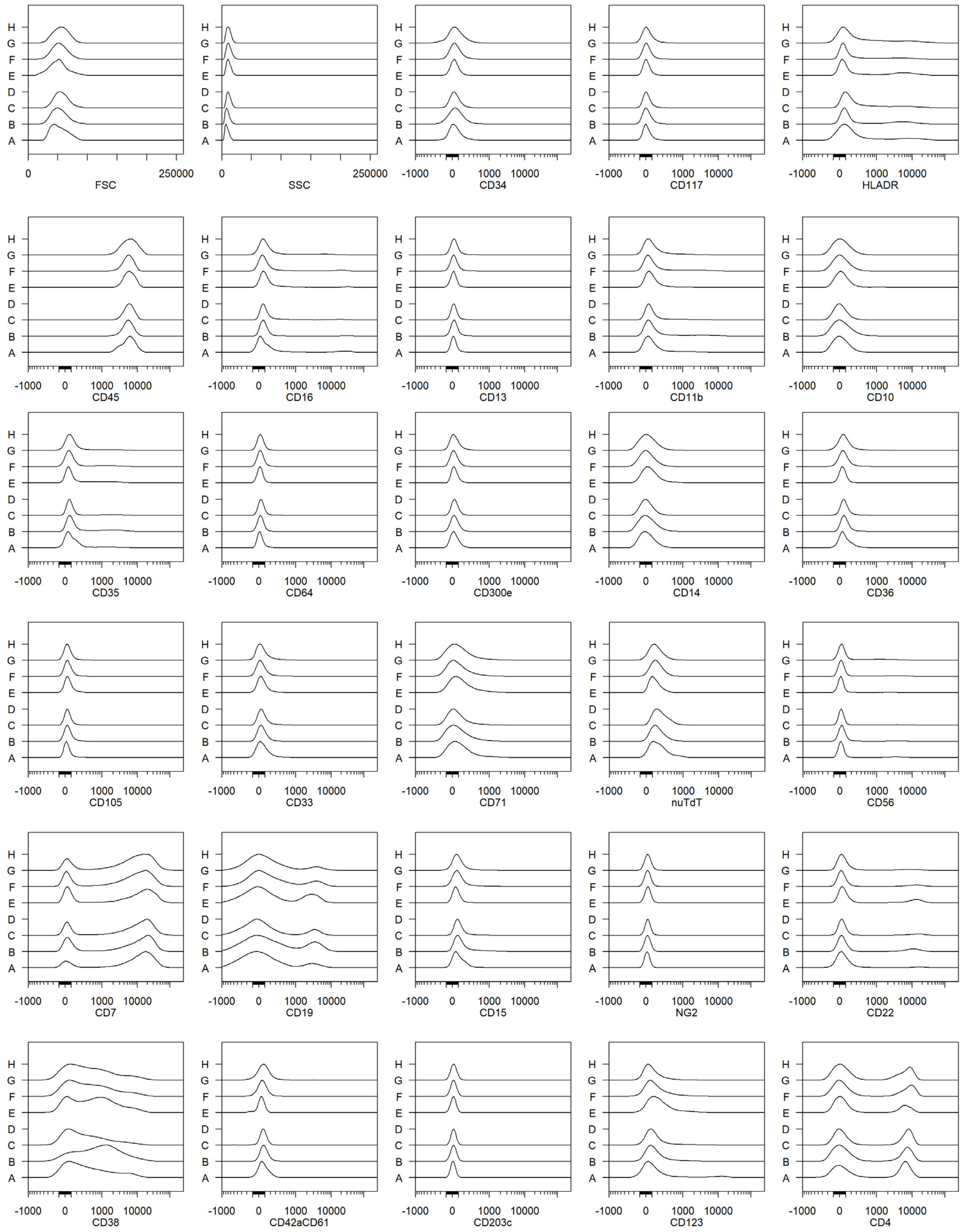

All cases:

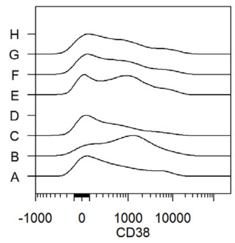

Pediatric cases:

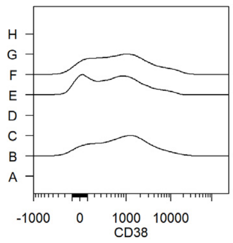

Adult casus:

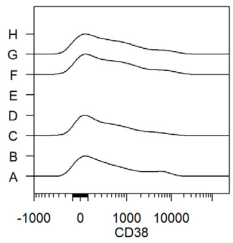

Supplementary Data S16 – Pipeline Validation

Theoretically, the existing cohort can be extend, by checking new cases against the established reference:

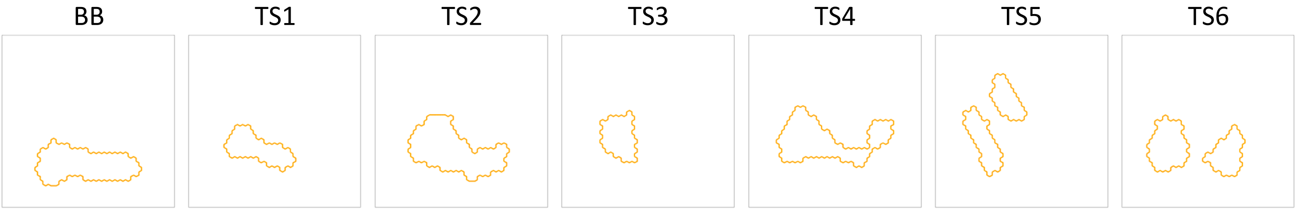

Validation

For validation purposes, in total 339 random cases from the initial cohort were re-analyzed from scratch (i.e. taking the raw FCS files as starting point). In other words, these gates were re-gated (according to the guideline in **Supplementary Data S2**), and subsequently passed through the pipe-line (checks from **Supplementary Data S3**, sanitization from **Supplementary Data S4**, check against BB reference from **Supplementary Data S7**, and check against TS reference from **Supplementary Data S9**). Thus, in the end, these 339 cases passed twice through the pipeline, with the only difference being the manual analysis (i.e. the lymphocyte gates).

Random example, initial analysis:

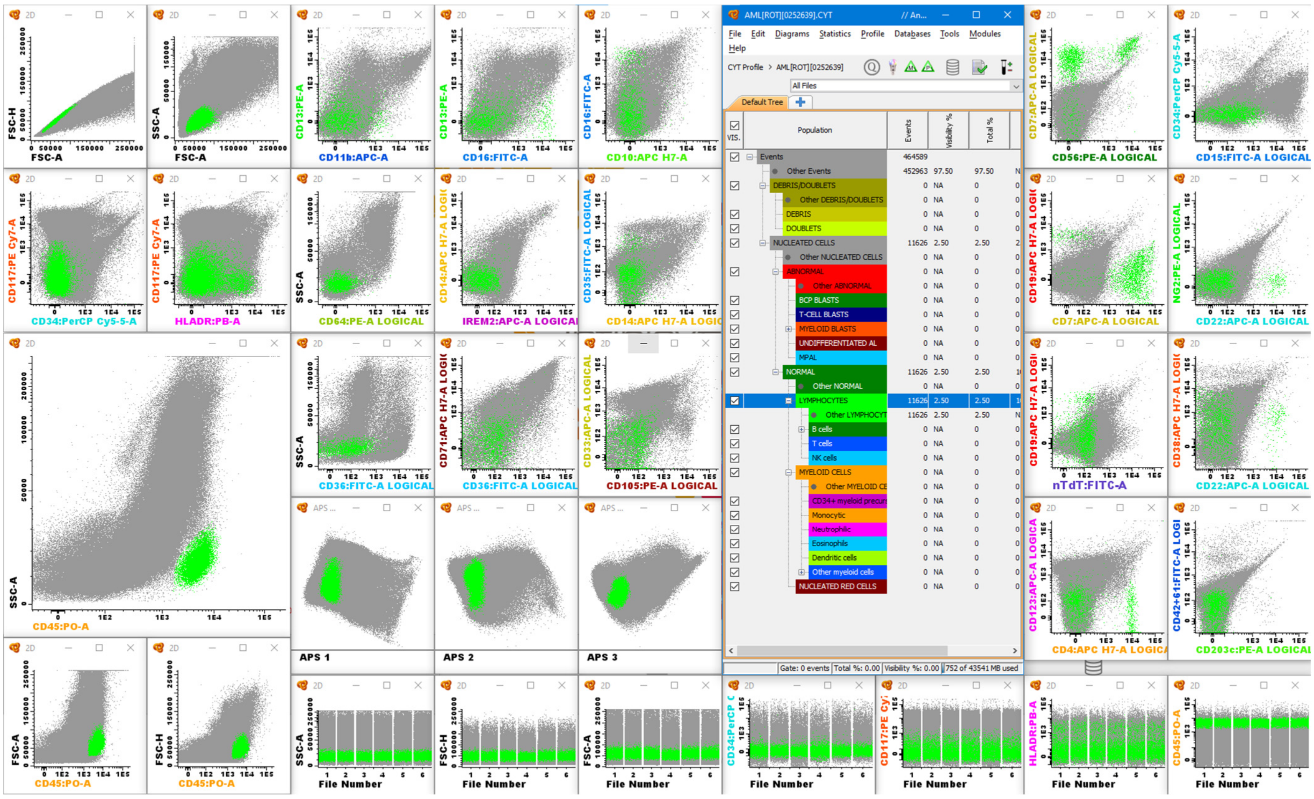

Random example (cont'ed), second analysis:

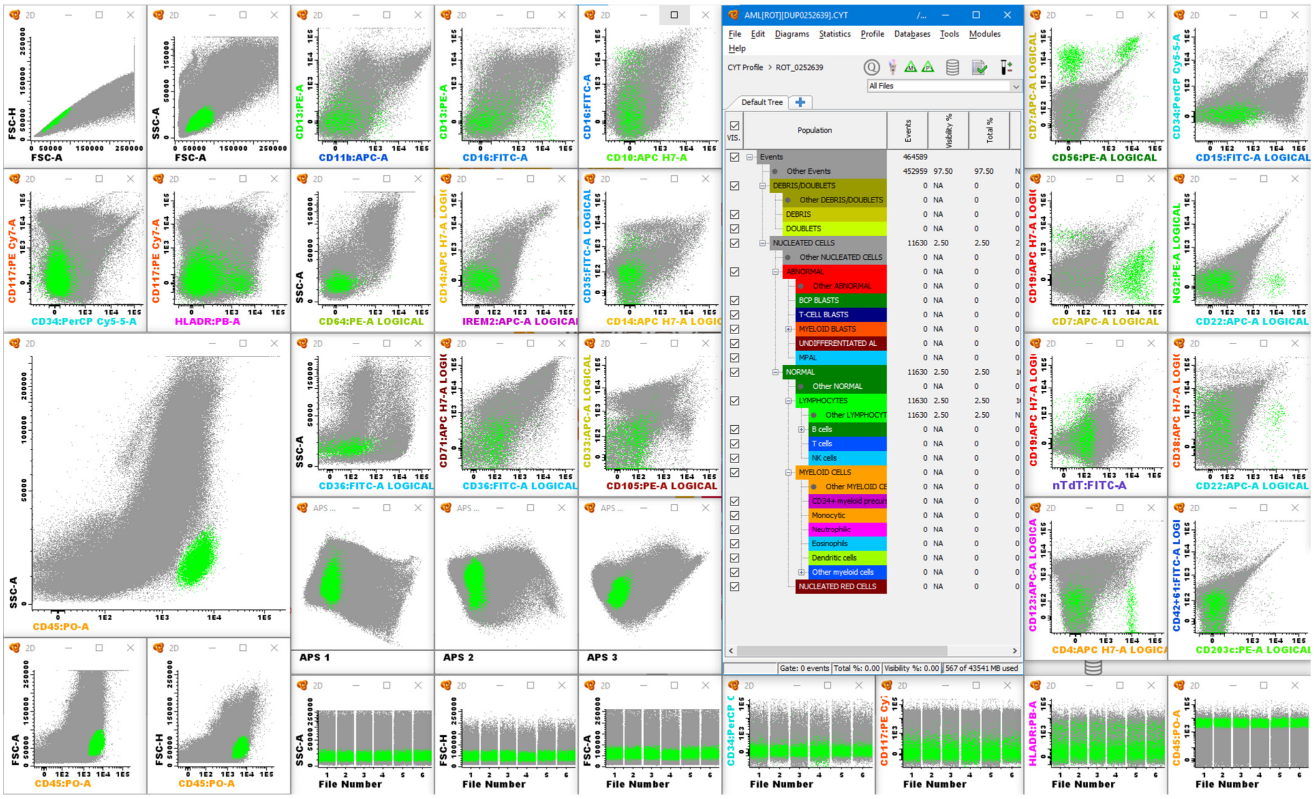

Validation – Results

The conclusions were identical for both passes. Each time, in total 80 cases were marked as non-optimal/incomplete, of which 19 cases were marked due to the BB marker being out-of-reference, and 61 cases due to the TS markers being out-of-reference. Essentially, the only differences between both passes were minor differences in the percentage of lymphocytes being considered out-of-reference (caused by slight differences in manual gating).

Comparing every out-of-range percentage from the initial pass against the corresponding out-of-range percentage from the validation pass, in less than five percent of cases, the difference was more than one percent (left panel of Figure below). As shown in the Figure below (right panel, details in **Supplementary Data S7**), the vast majority of cases featured out-of-range percentages that where either significantly below or above the cutoff of choice. In other words, the minor impact of manual gating on the out-of-range percentage is very unlikely to affect the conclusion (i.e. being included or excluded).

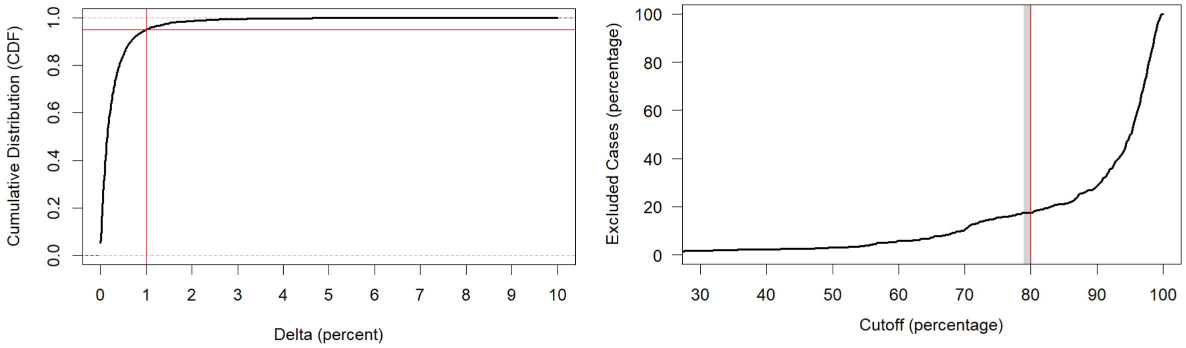

The red lines in the left figure represent the 5% cutoff (horizontal line) for the cumulative percentage of cases and the 1% cut-off (vertical line) for the difference in percentage out-of-reference lymphocytes between the initial and repeated analysis.

For example, for various cases, the BB percentages from the initial pass:

	BB.AML.1	BB.AML.2	BB.AML.3	BB.AML.4	BB.AML.5	BB.AML.6
**F.078***	95.1	93.9	95.7	93.8	93.3	95.7
**F.089**	88.5	87.9	63.1	76.1	88.7	93.7
**F.104***	97.8	97.9	97.8	96.7	97.8	70.6

In addition, the percentages from the validation pass, for the same cases:

	BB.AML.1	BB.AML.2	BB.AML.3	BB.AML.4	BB.AML.5	BB.AML.6
**F.078***	95.6	94.1	96.0	94.4	93.8	95.9
**F.089**	88.0	87.7	62.7	75.3	88.4	93.6
**F.104***	97.4	97.9	97.3	96.7	97.3	69.6

The BB results, for two cases with asterisk (left and right), for the initial and validation pass (top and bottom) are shown below:

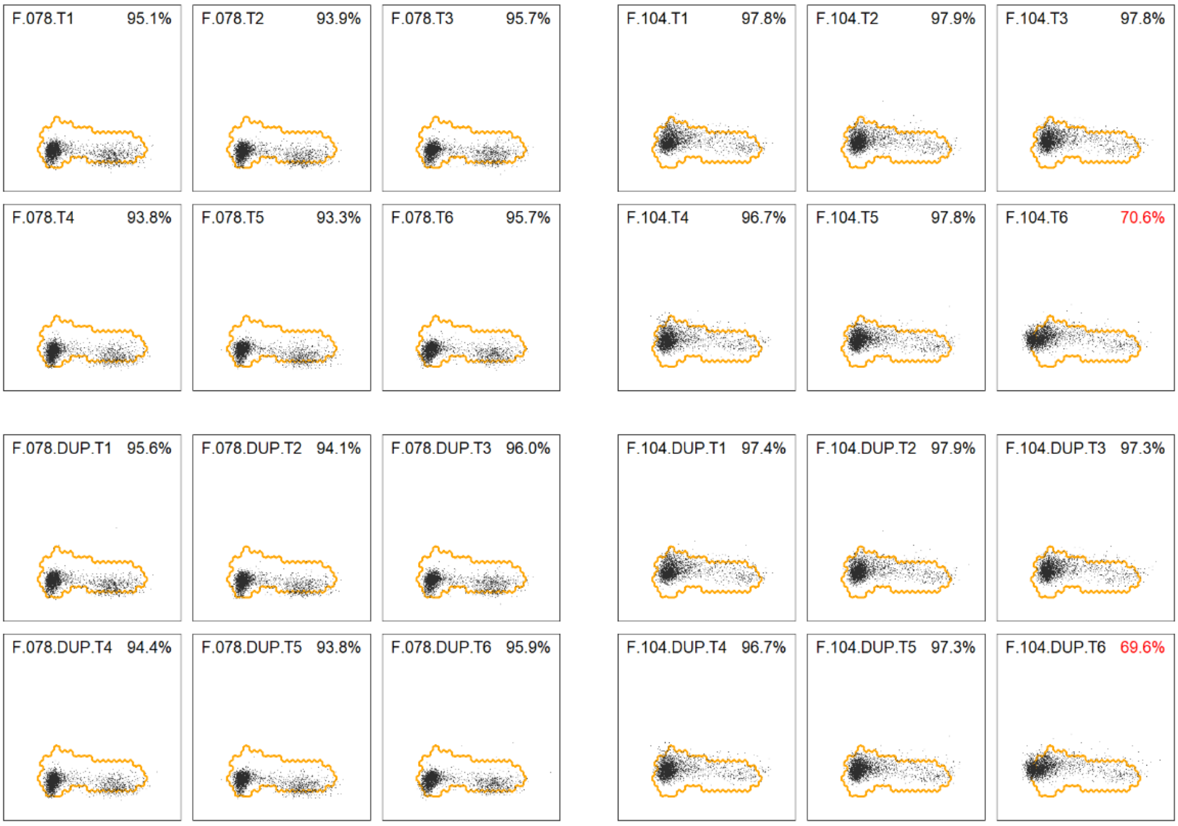

For example, cases that passed the BB checks, the TS percentages from the initial pass:

	TS.AML.1	TS.AML.2	TS.AML.3	TS.AML.4	TS.AML.5	TS.AML.6
**F.075**	97.5	98.0	96.5	94.1	92.7	94.2
**F.078**	94.6	96.1	96.3	96.3	95.6	97.1

In addition, the percentages from the validation pass, for the same cases:

	TS.AML.1	TS.AML.2	TS.AML.3	TS.AML.4	TS.AML.5	TS.AML.6
**F.075**	97.9	98.4	96.6	94.3	93.6	93.8
**F.078**	94.7	96.2	96.2	96.3	95.8	97.1

The TS results, for two cases (left and right), for the initial and validation pass (top and bottom):

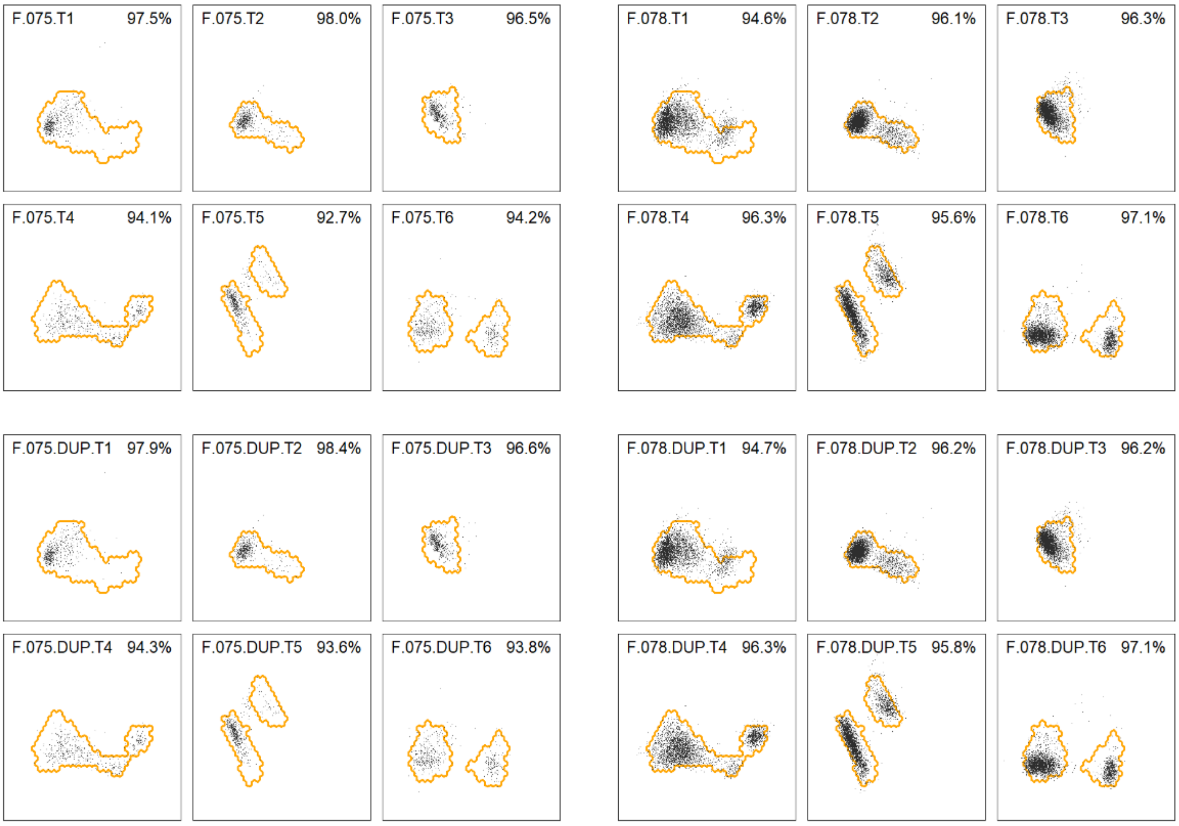

Supplementary Data S17 – Normal BM samples

42 normal BM samples were uploaded to the EuroFlow server. Similar QA was performed as for the AML cases.

Table S17.1. Overview of exclusions after initial checks

Process	Number of cases
Intake	48
Missing Markers	-1
Flagged in BB QA	-7
Flagged in TS QA	-12
Fixed (compensation issue)	+6
**Total after QA**	**28**

Details per center:

Center	Included	Passed QA	Fixed	Total in data set	% included in data set
A	0	0	0	0	NA
B	3	1	0	1	33%
C	1	1	0	1	100%
D	13	9	2	11	85%
E	0	0	0	0	NA
F	11	5	0	5	45%
G	20	6	4	10	50%
H	0	0	0	0	NA

Example of normal BM samples that passed the QA for the BB markers:

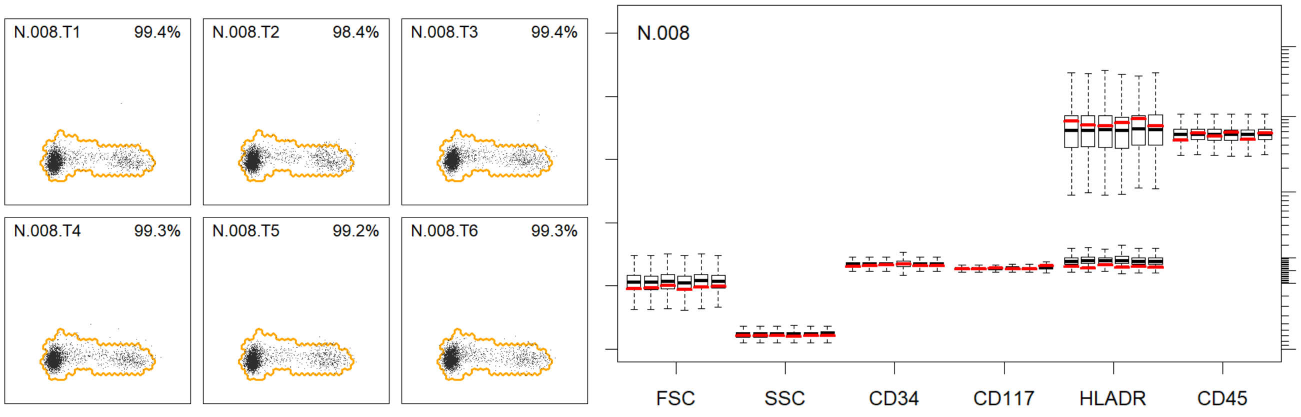

Example of normal BM samples that passed the QA for the TSB markers:

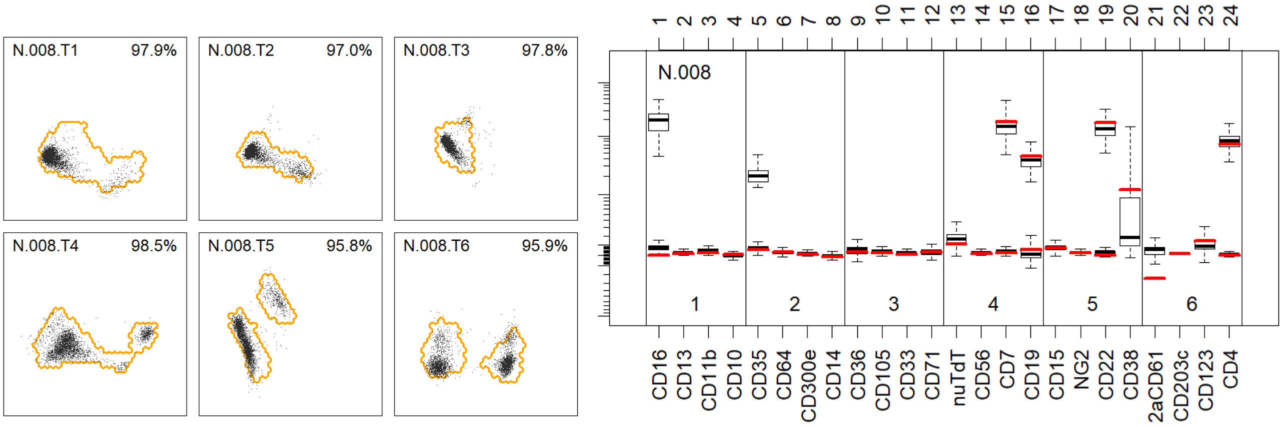

Example of normal BM samples flagged for BB markers:

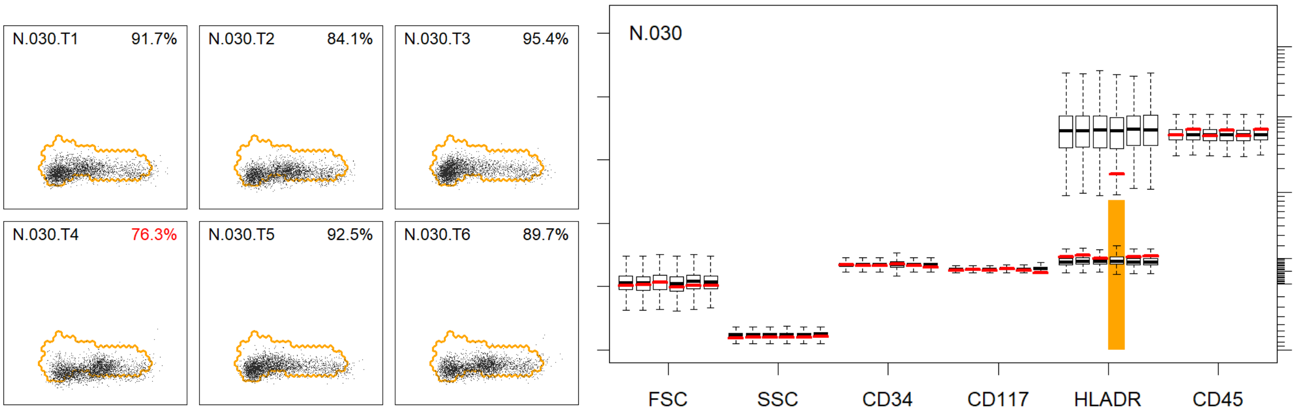

Example of normal BM samples flagged for TS markers:

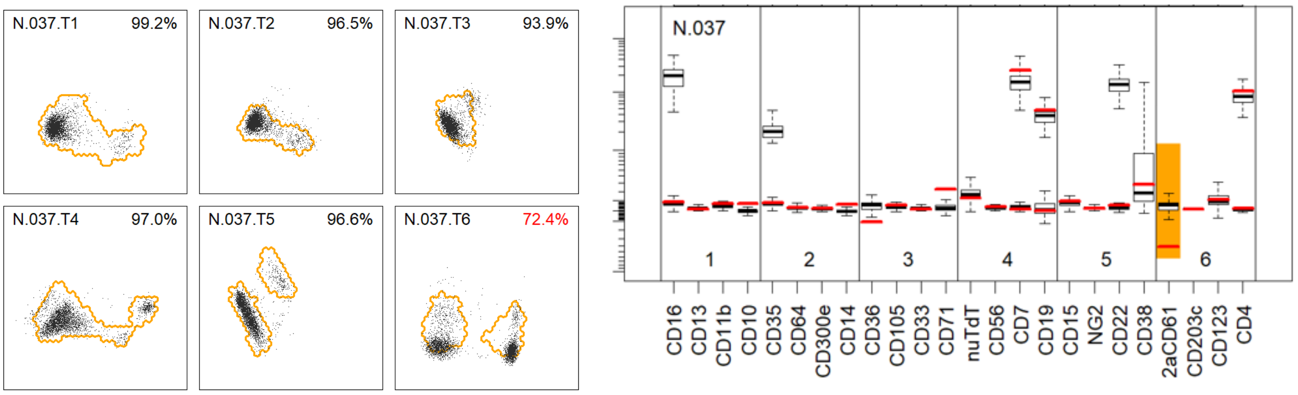

Percentage of tube flagged in total of flagged cases							
	n	BB1	BB2	BB3	BB4	BB5	BB6
normal BM	7	0	71	0	100	14	43
		TS1	TS2	TS3	TS4	TS5	TS6
normal BM	12	25	25	8	0	25	100

Flagging for backbone markers particularly occurred for tube 4, mainly due to HLADR being out of reference. All these flagged samples were from one center (G). For the TS QA, most flagged cases considered tube 6 with CD42a.CD61 and/or CD123 out of reference. These results are in line with the findings in the QA of the AML samples. Six of 12 flagged cases in the TS QA could be fixed by adjusting a single compensation value.

Table S17.1. Characteristics of the 28 normal BM samples

	**Normal bone marrow samples**
n	28
Age (median, range)	23 (5–70) [a]
Gender (M/F)	11/14 [b]
Center	B 1; C 1; D 11; F 5; G 10

[a] No data available for 6 subjects; [b] no data available for 3 subjects.
